# Supplementary material for: Synthesis, Antifungal Activity, 3D-QSAR, and Molecular Docking Study of Novel Menthol-Derived 1,2,4-Triazole-thioether Compounds
Source: Molecules. 2021 Nov 17;26(22):6948. doi: 10.3390/molecules26226948 (PMC8618492; doi:10.3390/molecules26226948)
Supplement: Supplementary file 1 [file molecules-26-06948-s001.zip › molecules-1454738-supplementary.pdf]

Supplementary Materials

Synthesis, Antifungal Activity, 3D-QSAR and Molecular Docking Study of Novel Menthol-Derived 1,2,4-Triazole-Thioether Compounds

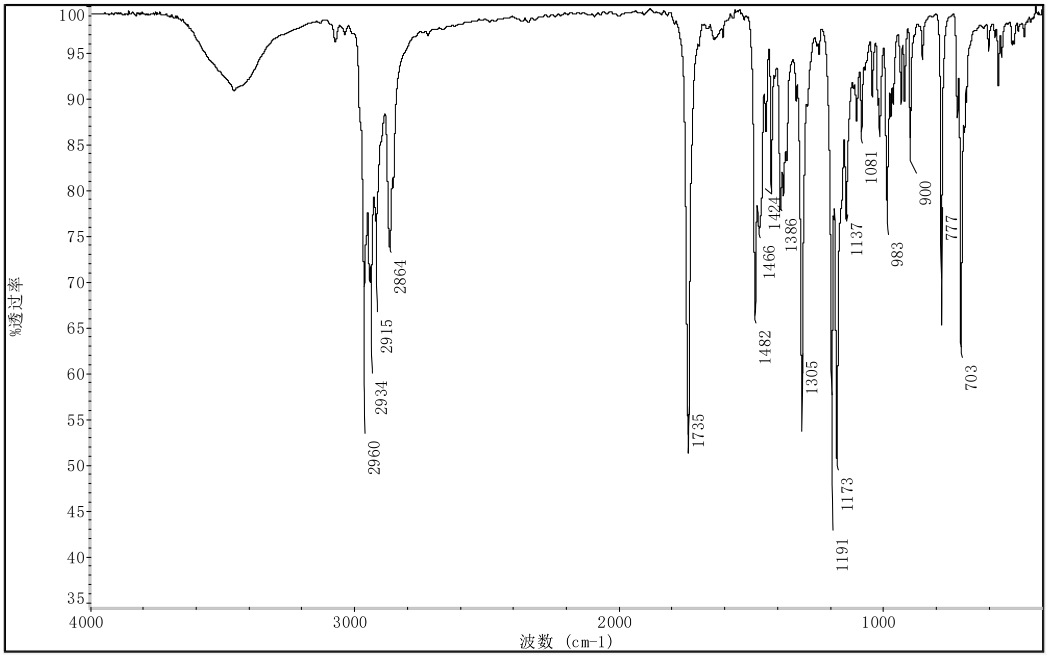

Figure S1. FT-IR spectrum of Compound **5a**(R=Ph)

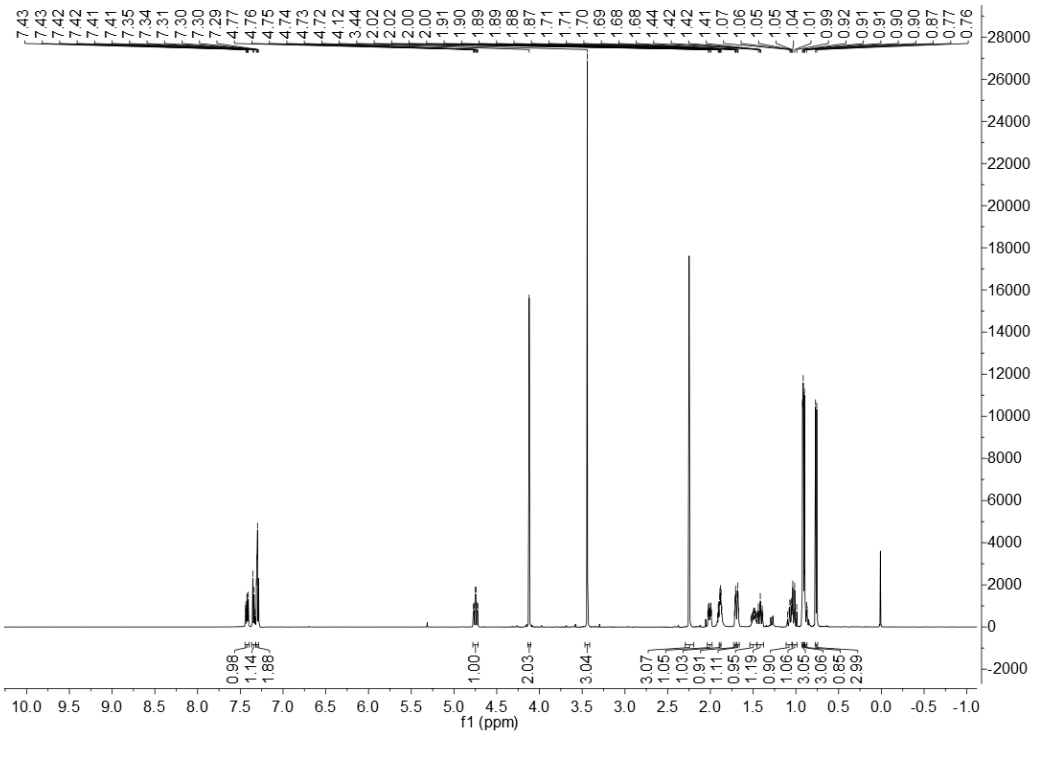

Figure S2. ^{1}H-NMR spectrum of Compound **5a**(R=Ph) in $CDCl_3$

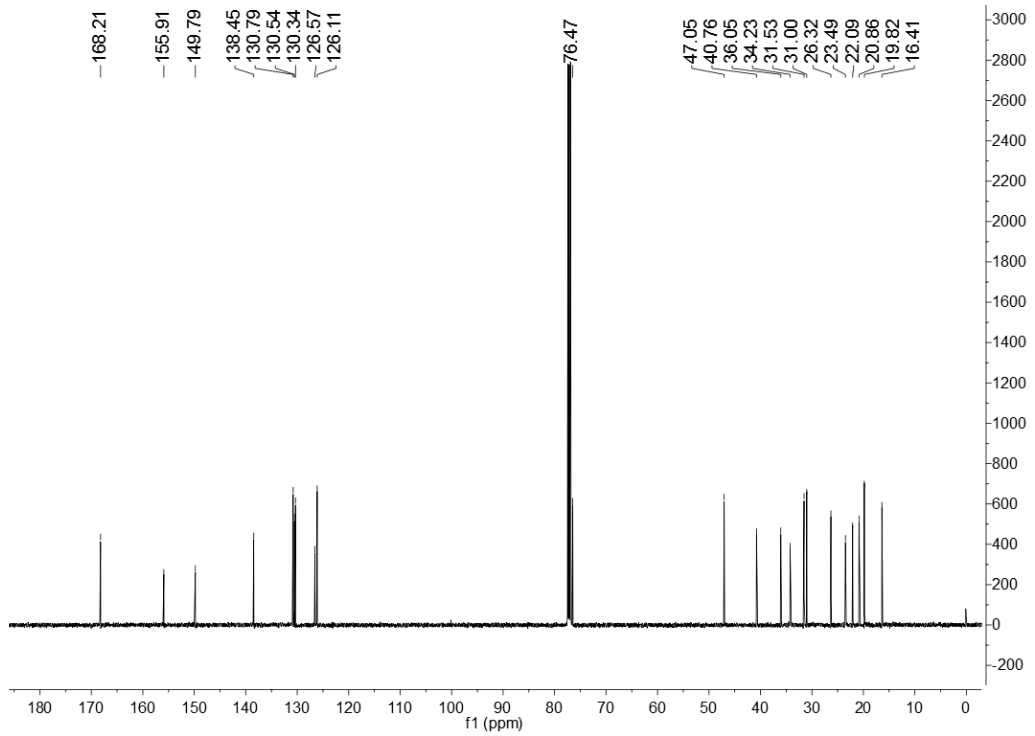

Figure S3. ^{13}C-NMR spectrum of Compound **5a**(R=Ph) in $CDCl_3$.

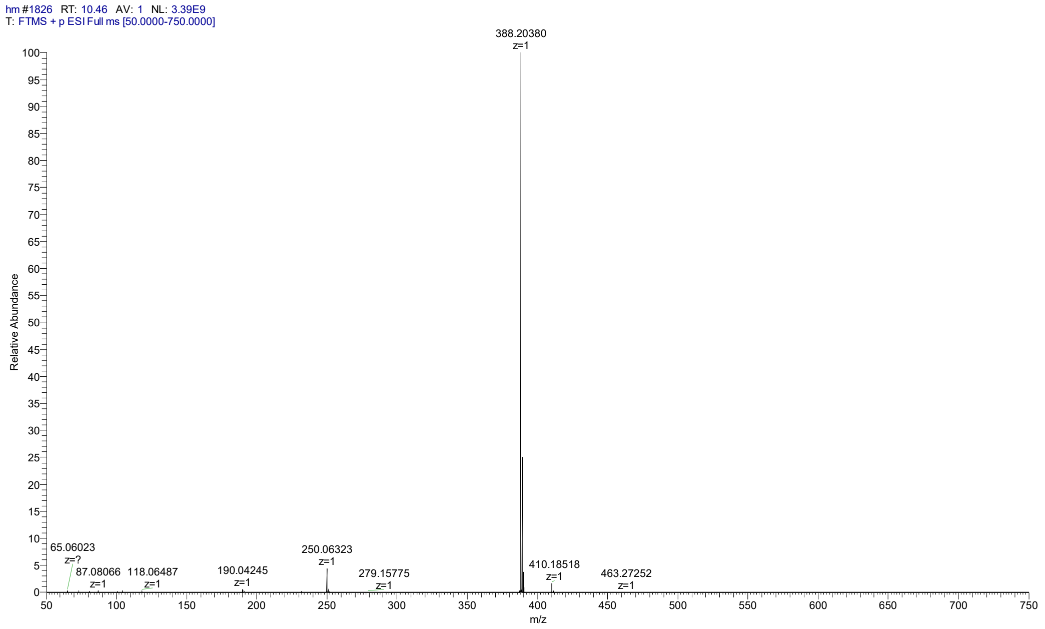

Figure S4. ESI-MS spectrum of Compound **5a**(R=Ph).

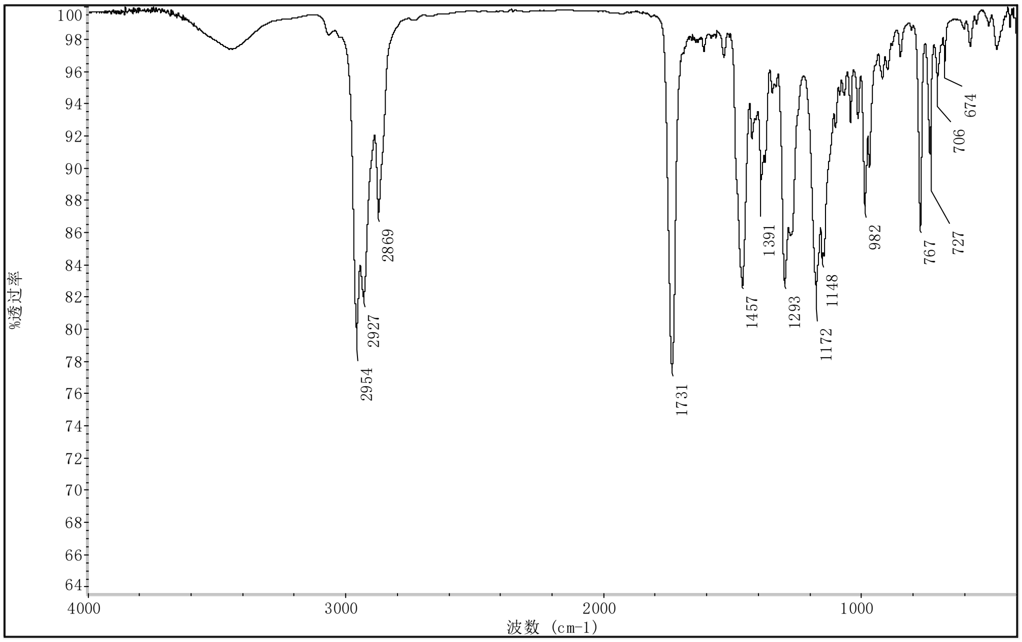

Figure S5. FT-IR spectrum of Compound **5b**(R=*o*-CH_3 Ph)

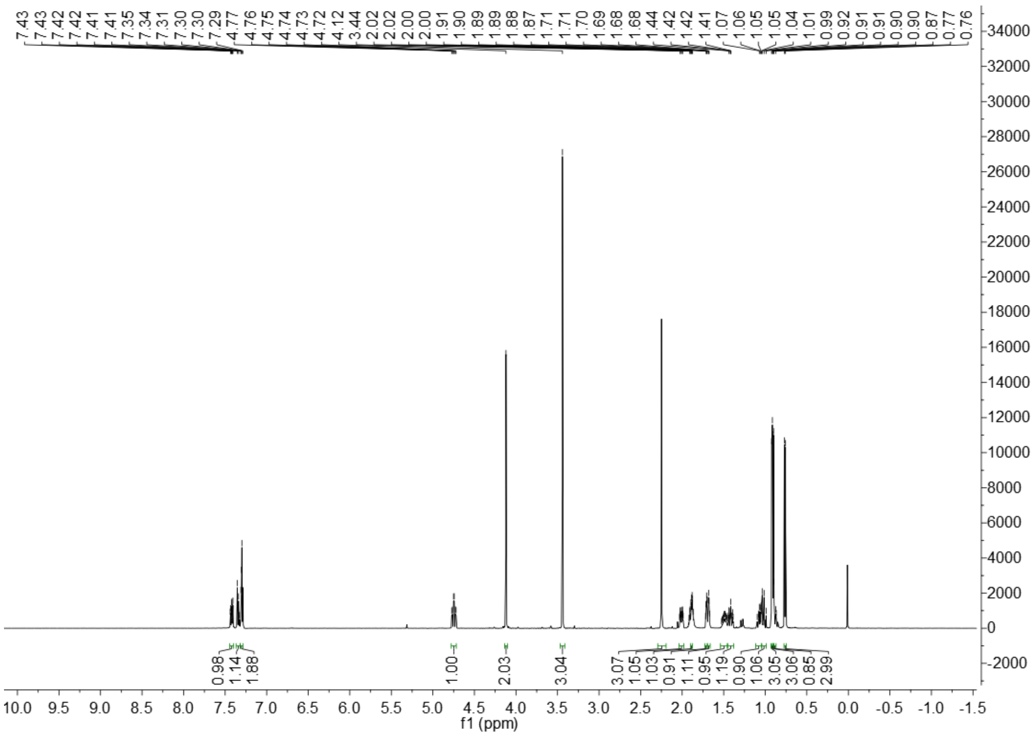

Figure S6. ^{1}H-NMR spectrum of Compound **5b**(R=*o*-CH_3 Ph) in $CDCl_3$

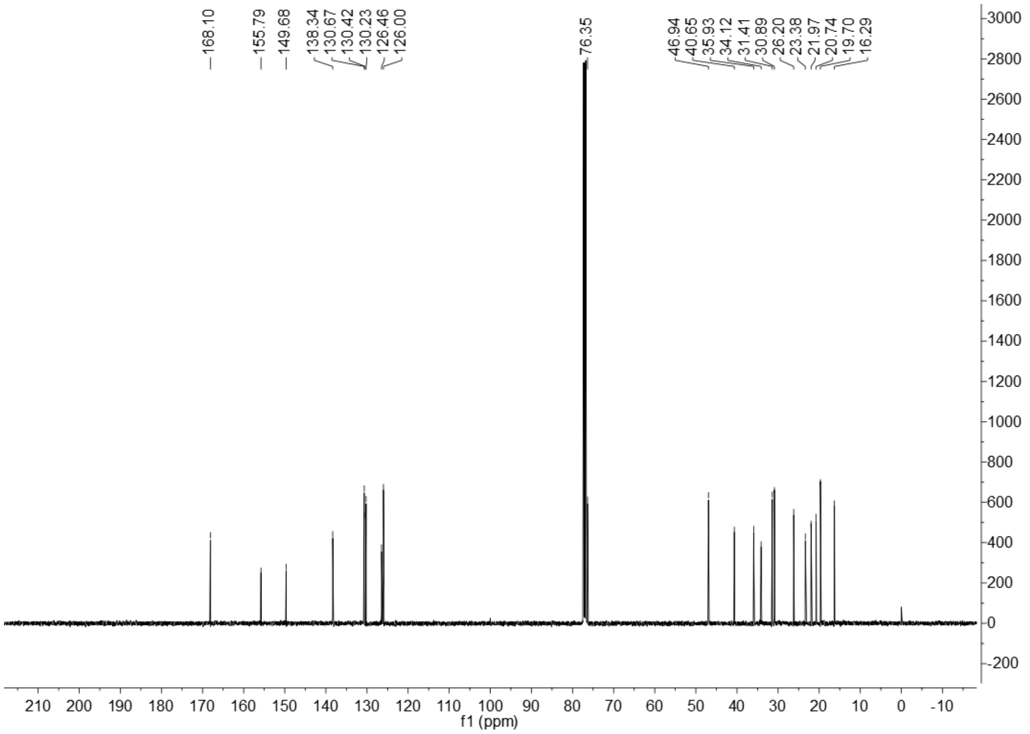

Figure S7. ^{13}C-NMR spectrum of Compound **5b**(R=*o*-CH_3 Ph) in $CDCl_3$.

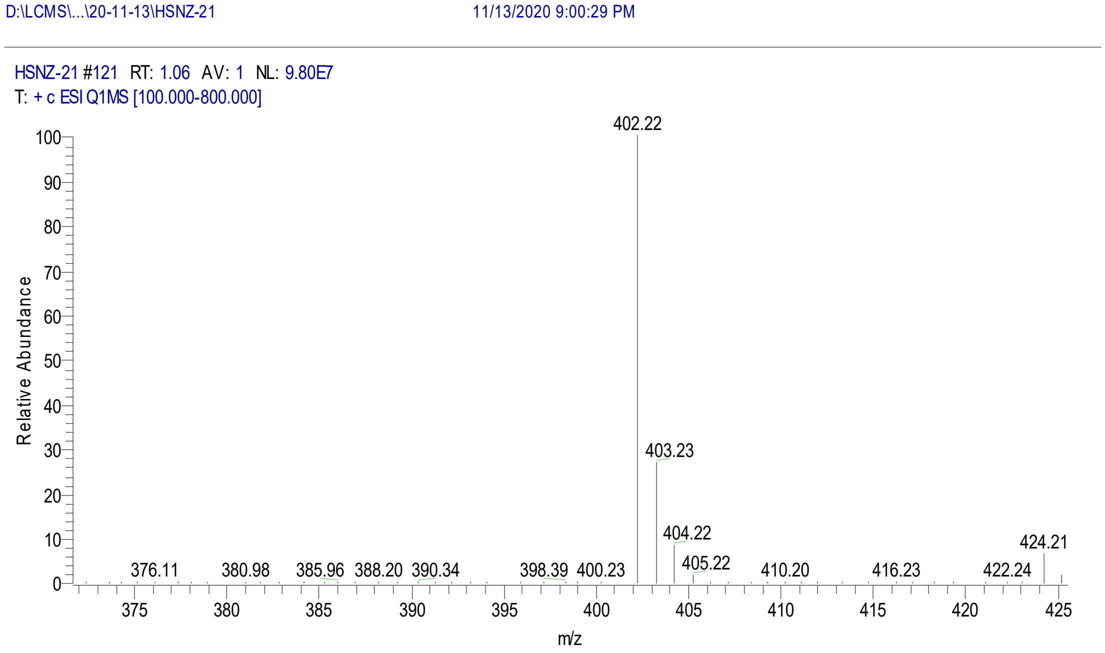

Figure S8. ESI-MS spectrum of Compound **5b**(R=*o*-CH_3 Ph).

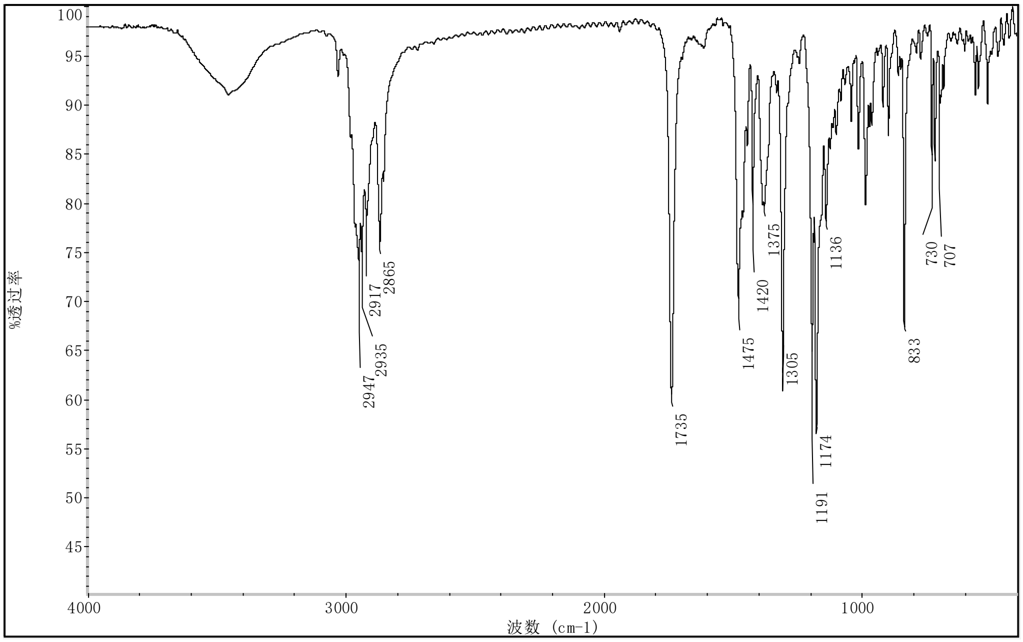

Figure S9. FT-IR spectrum of Compound **5c**(R=*p*-CH_3 Ph)

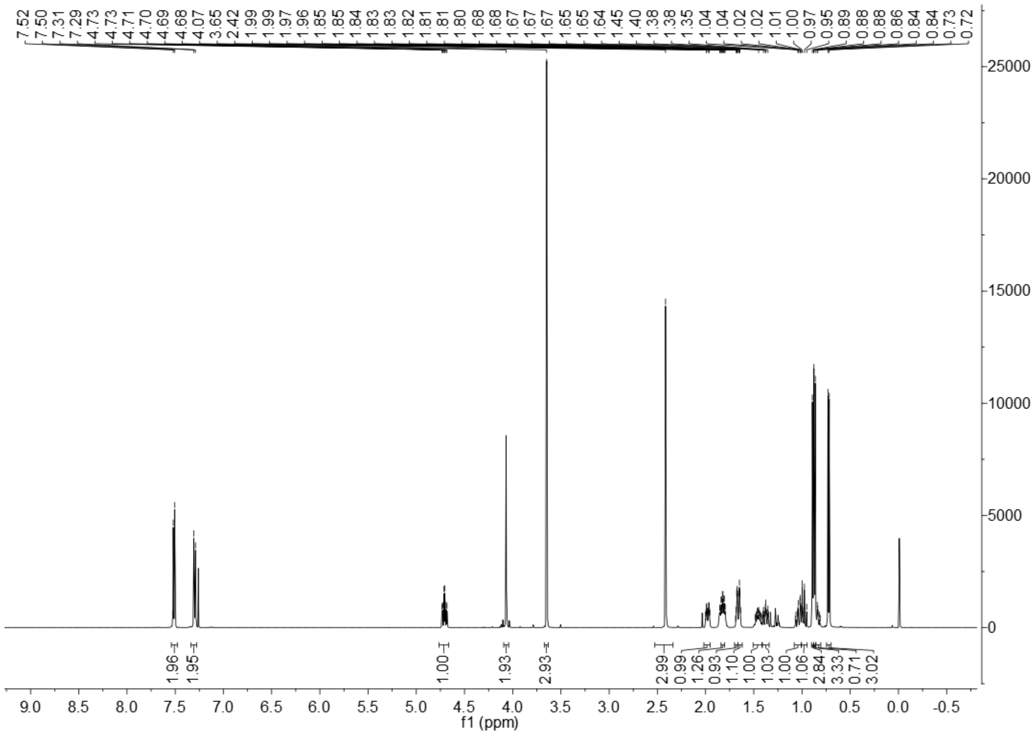

Figure S10. ^{1}H-NMR spectrum of Compound **5c**(R=*p*-CH_3 Ph)

in $CDCl_3$

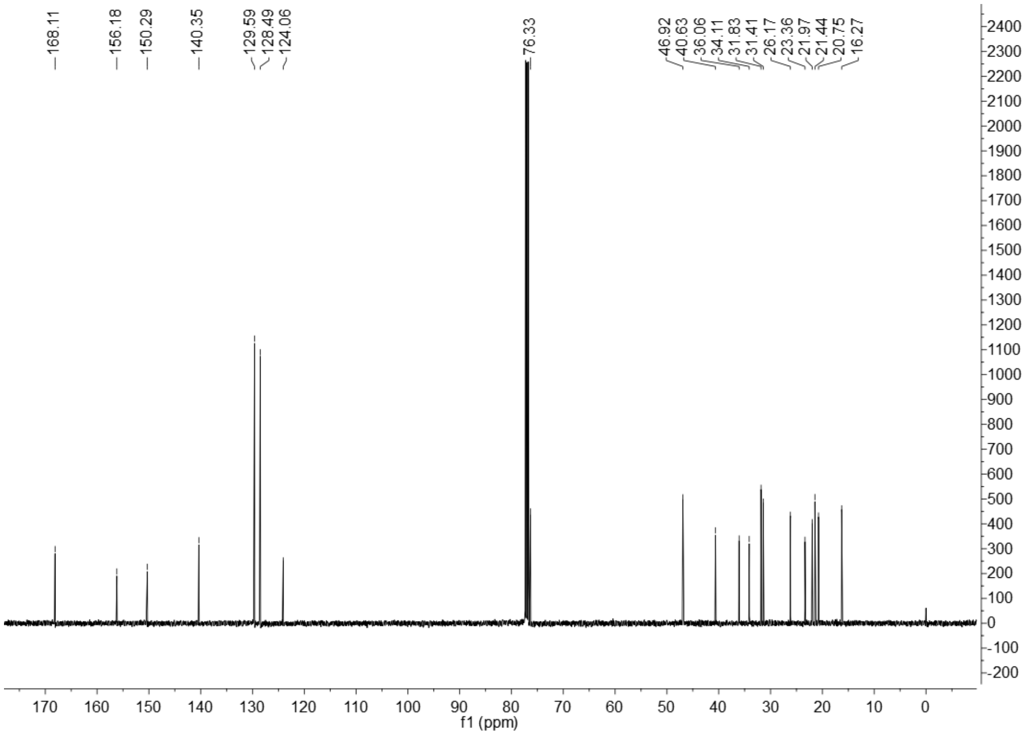

Figure S11. ^{13}C-NMR spectrum of Compound **5c**(R=*p*-CH3 Ph) in CDCl3.

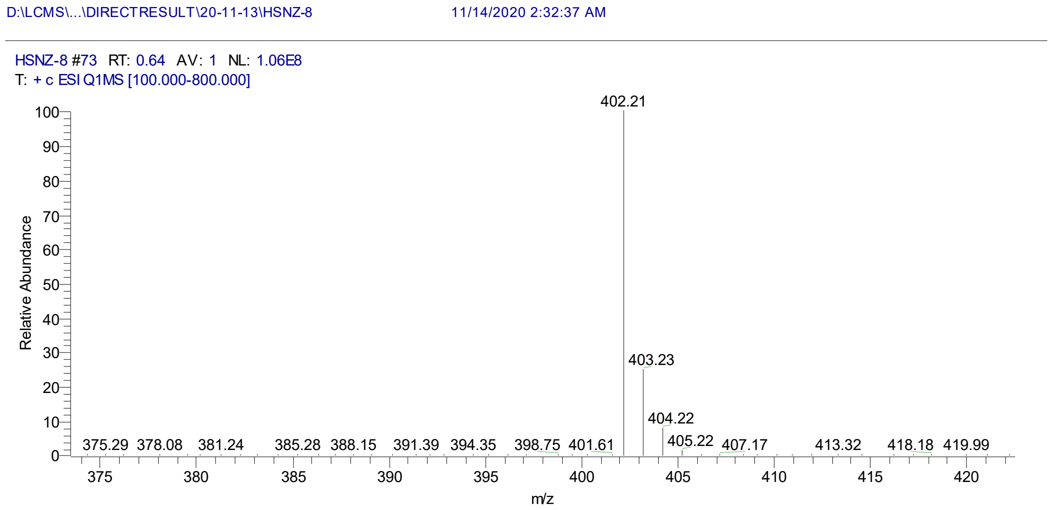

Figure S12. ESI-MS spectrum of Compound **5c**(R=*p*-CH3 Ph).

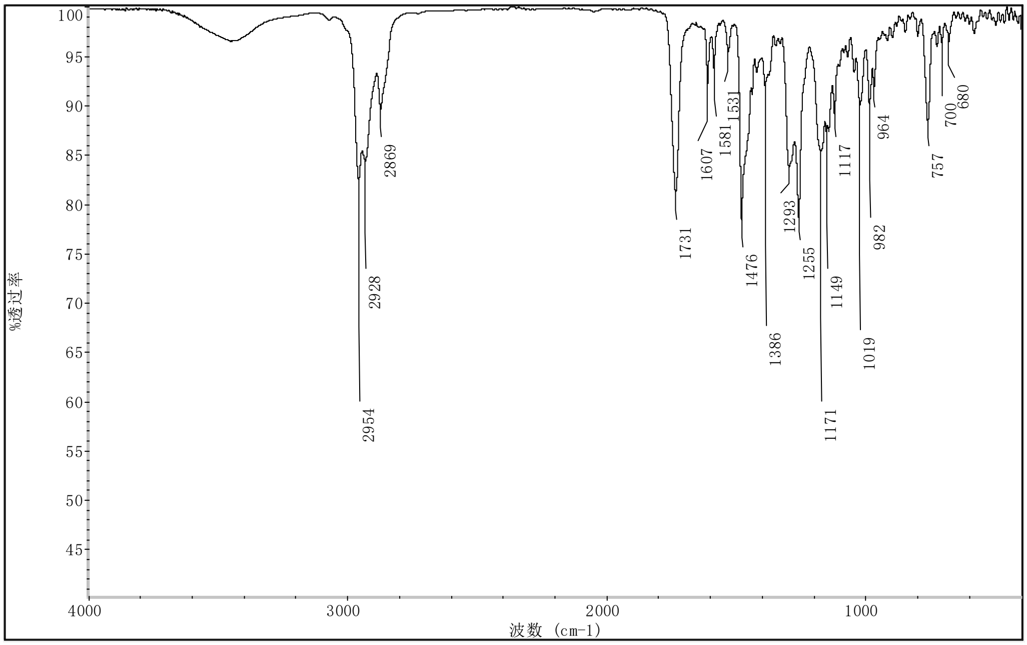

Figure S13. FT-IR spectrum of Compound **5d**(R=*o*-OCH_3 Ph)

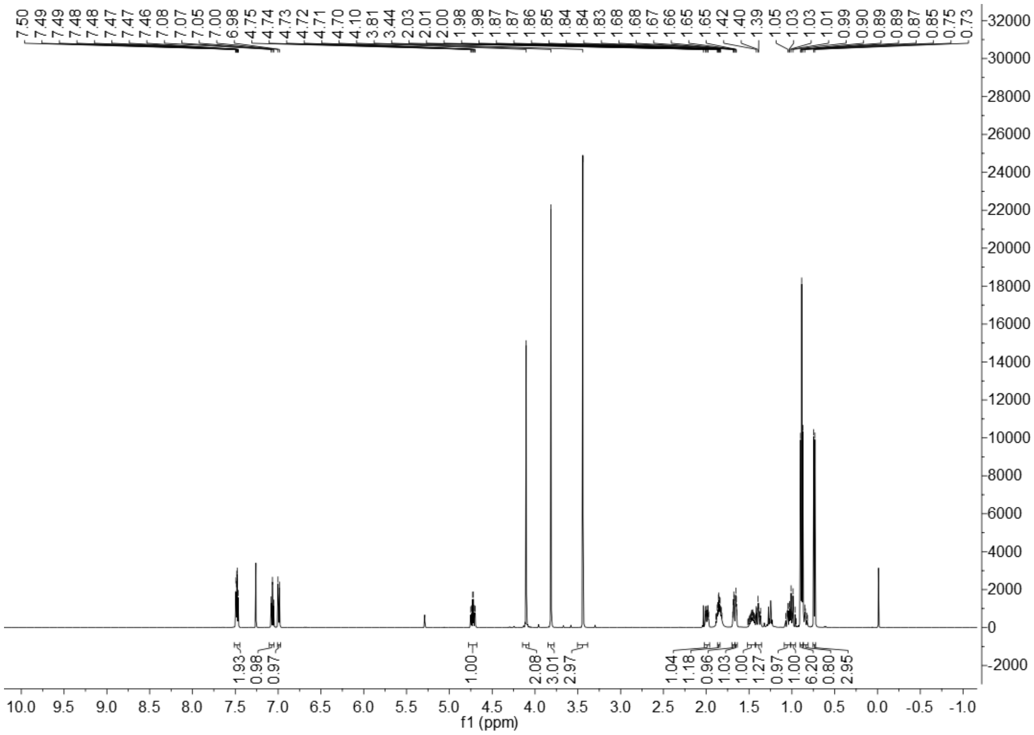

Figure S14. ^{1}H-NMR spectrum of Compound **5d**(R=*o*-OCH_3 Ph)

in $CDCl_3$

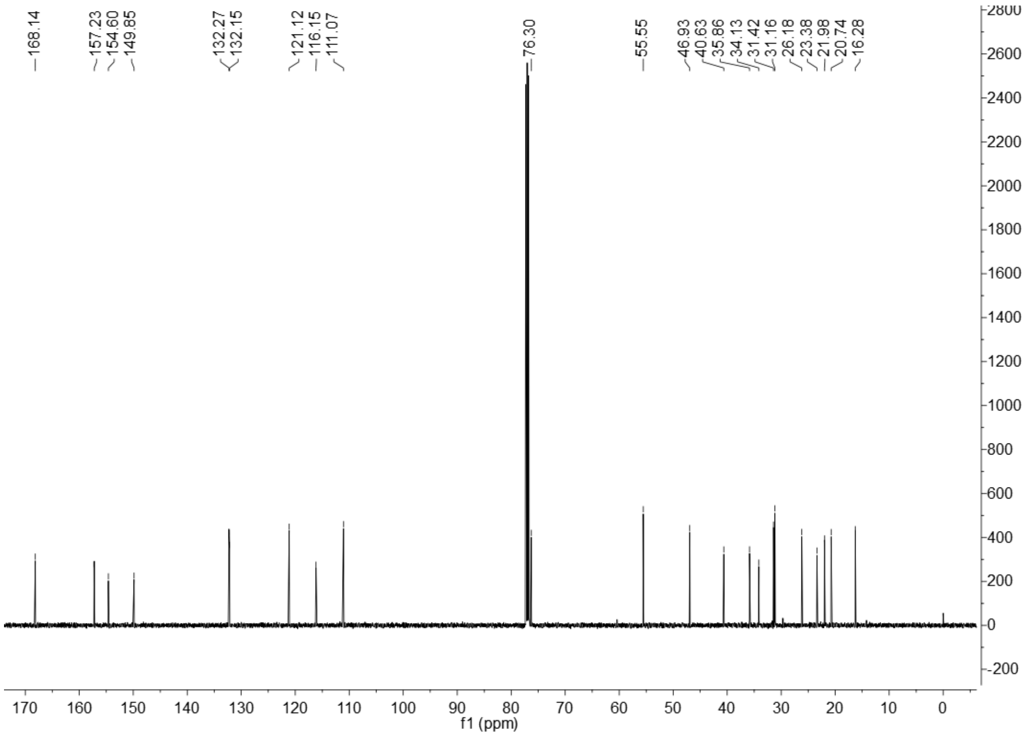

Figure S15. ^{13}C-NMR spectrum of Compound **5d**(R=*o*-OCH_3 Ph) in $CDCl_3$.

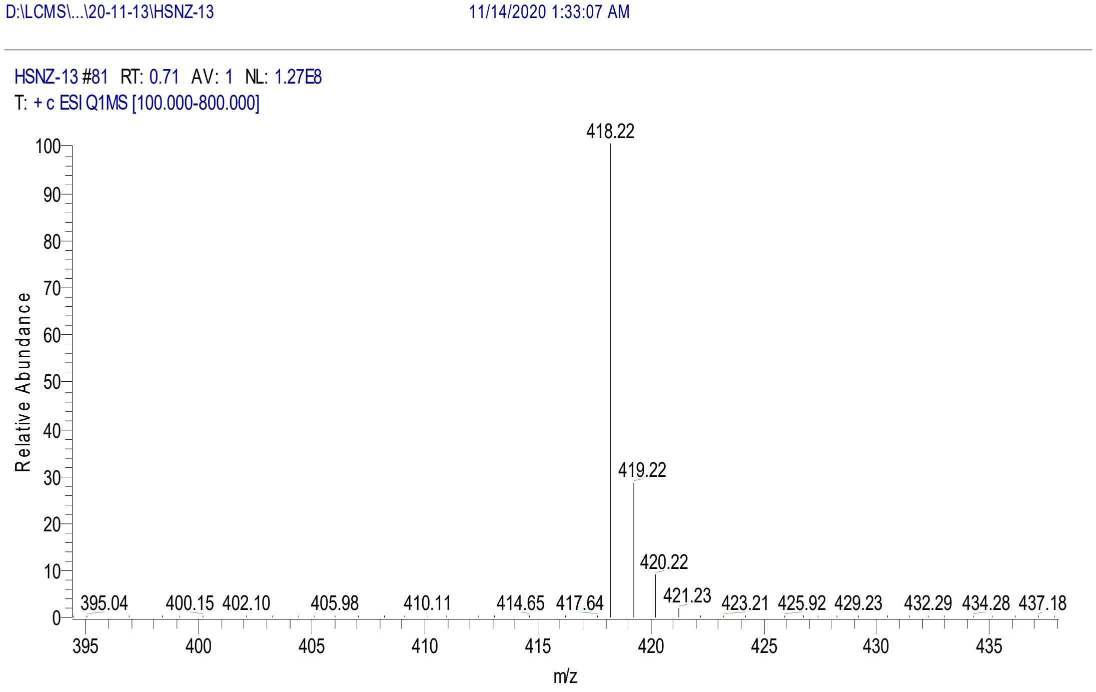

Figure S16. ESI-MS spectrum of Compound **5d**(R=*o*-OCH_3 Ph).

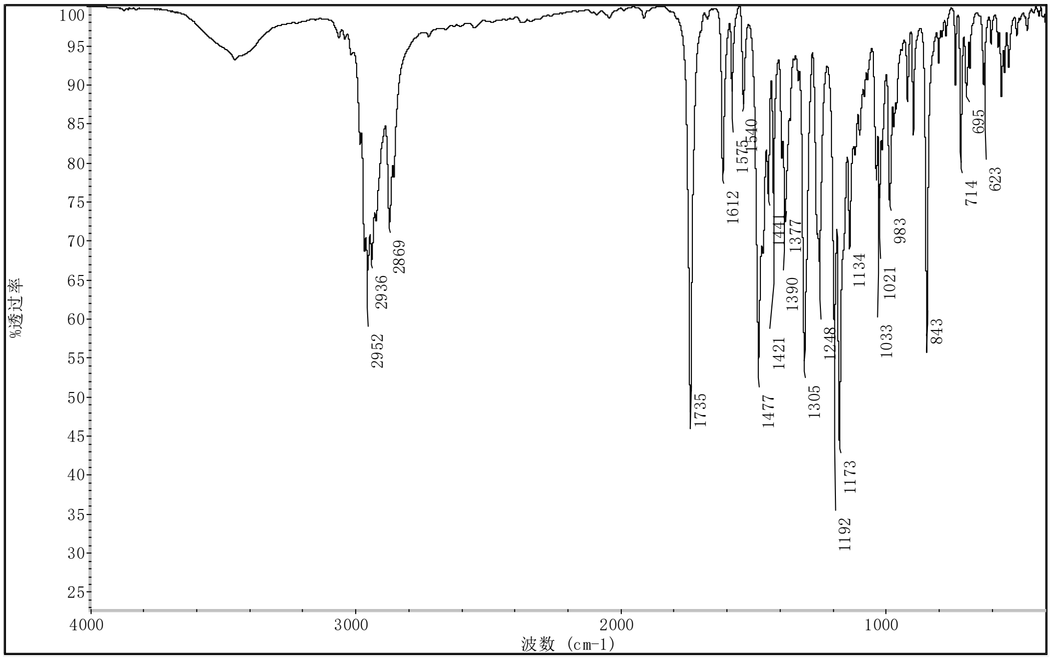

Figure S17. FT-IR spectrum of Compound **5e**(R=*p*-OCH$_3$ Ph)

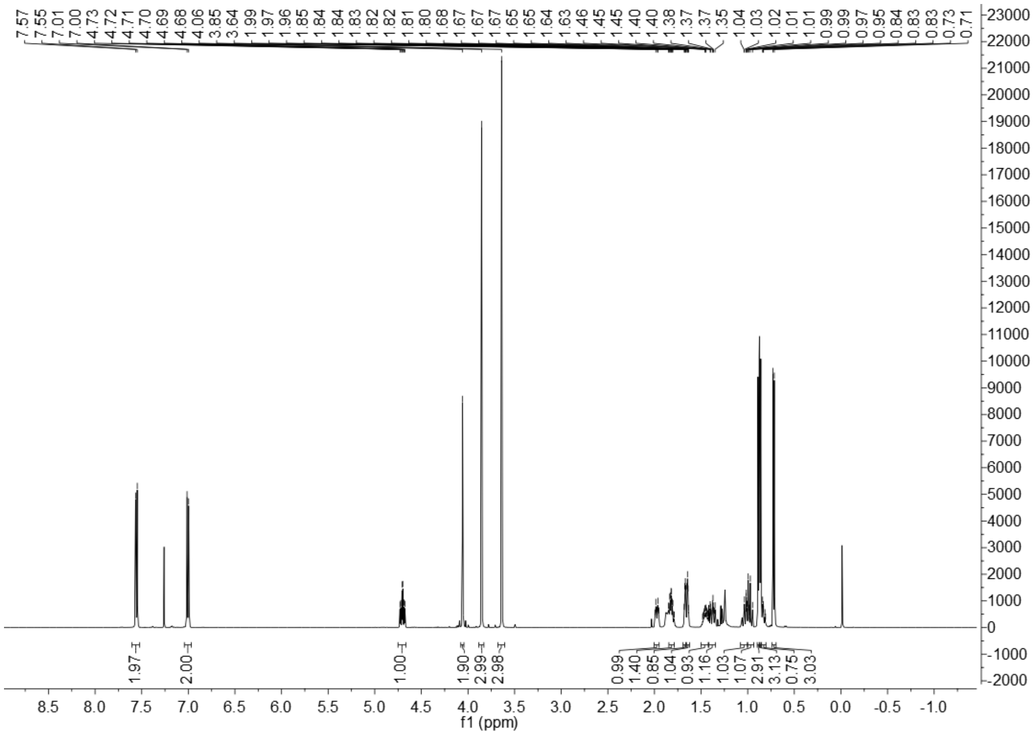

Figure S18. ^{1}H-NMR spectrum of Compound **5e**(R=*p*-OCH$_3$ Ph)

in CDCl$_3$

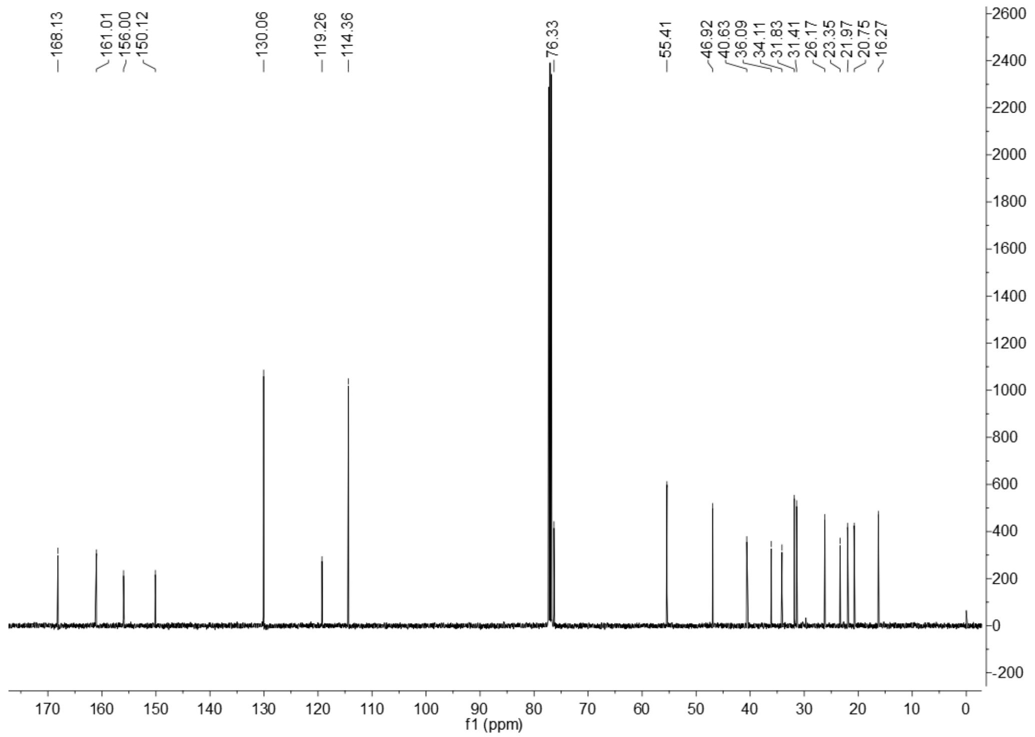

Figure S19. ^{13}C-NMR spectrum of Compound **5e**(R=*p*-OCH_3 Ph) in $CDCl_3$.

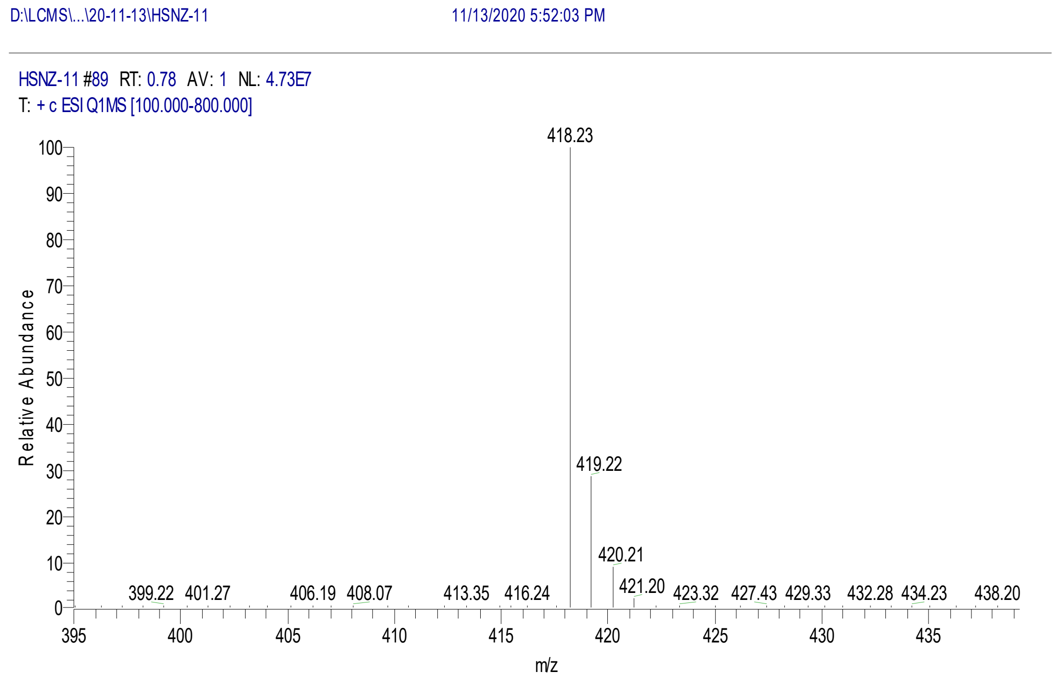

Figure S20. ESI-MS spectrum of Compound **5e**(R=*p*-OCH_3 Ph).

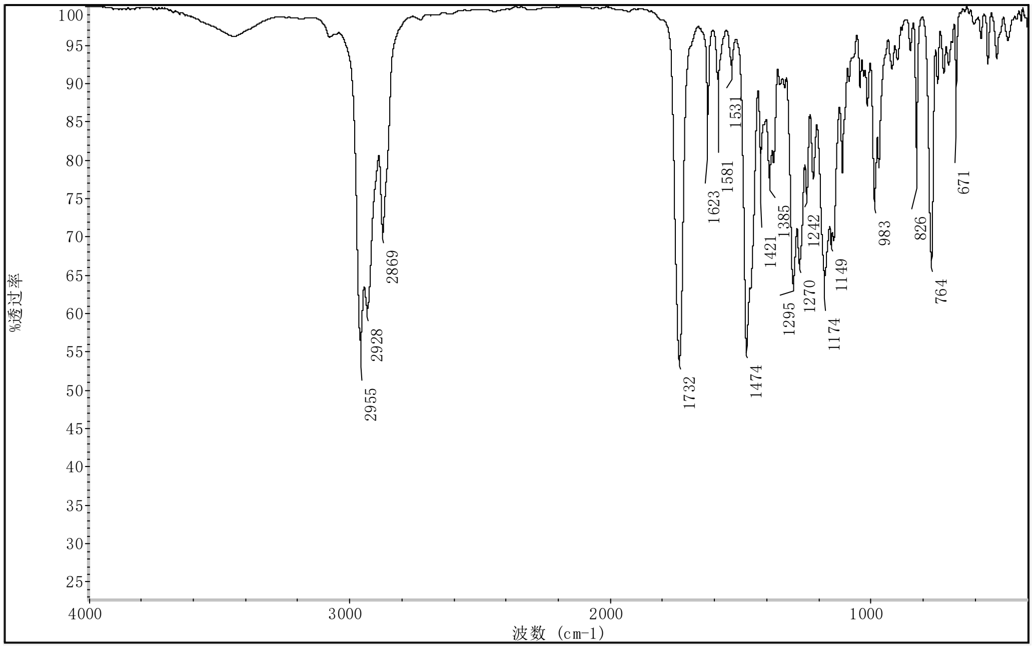

Figure S21. FT-IR spectrum of Compound **5f**(R=*o*-F Ph)

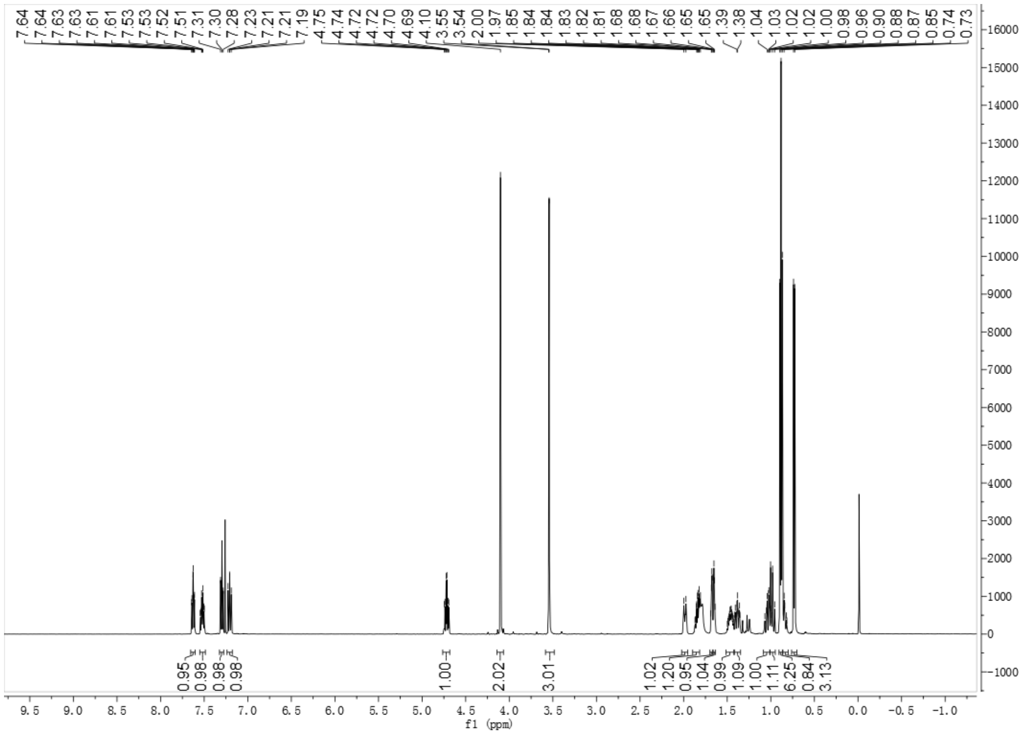

Figure S22. ^{1}H-NMR spectrum of Compound **5f**(R=*o*-F Ph) in $CDCl_3$

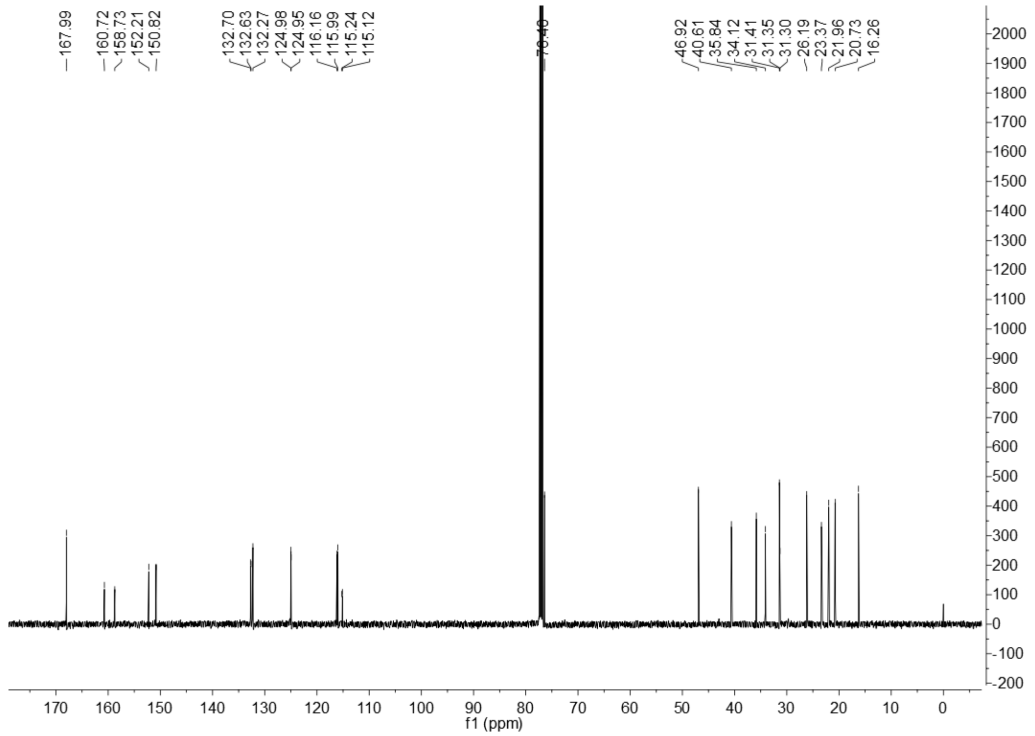

Figure S23. ^{13}C-NMR spectrum of Compound **5f**(R=*o*-F Ph) in $CDCl_3$.

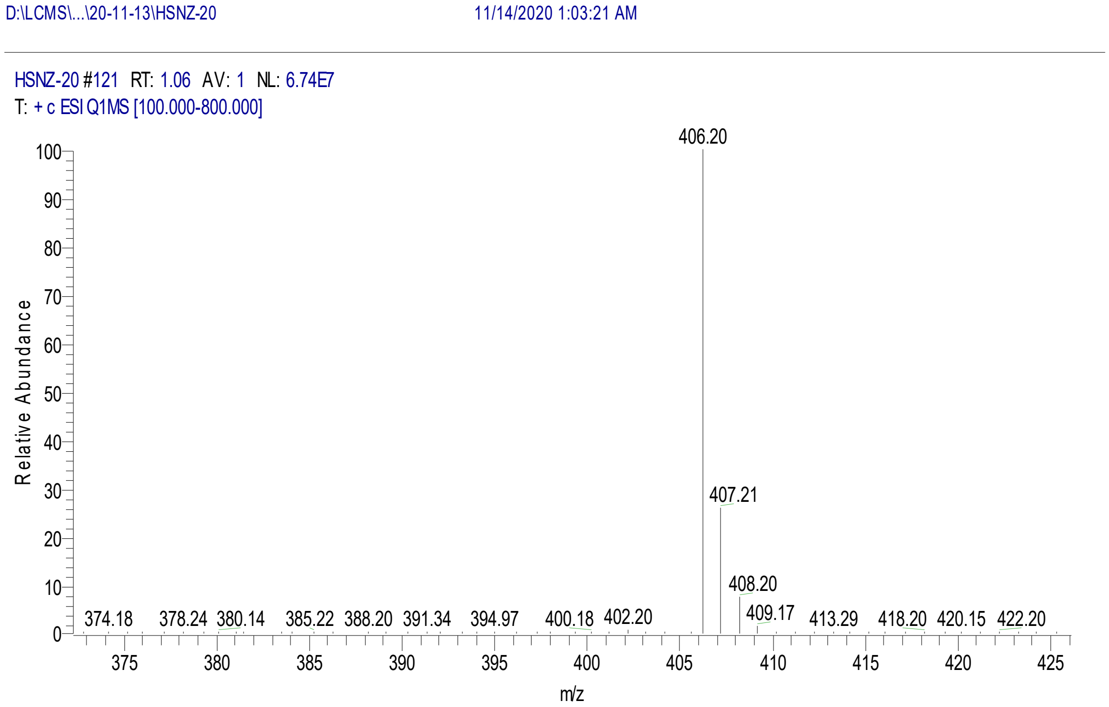

Figure S24. ESI-MS spectrum of Compound **5f**(R=*o*-F Ph).

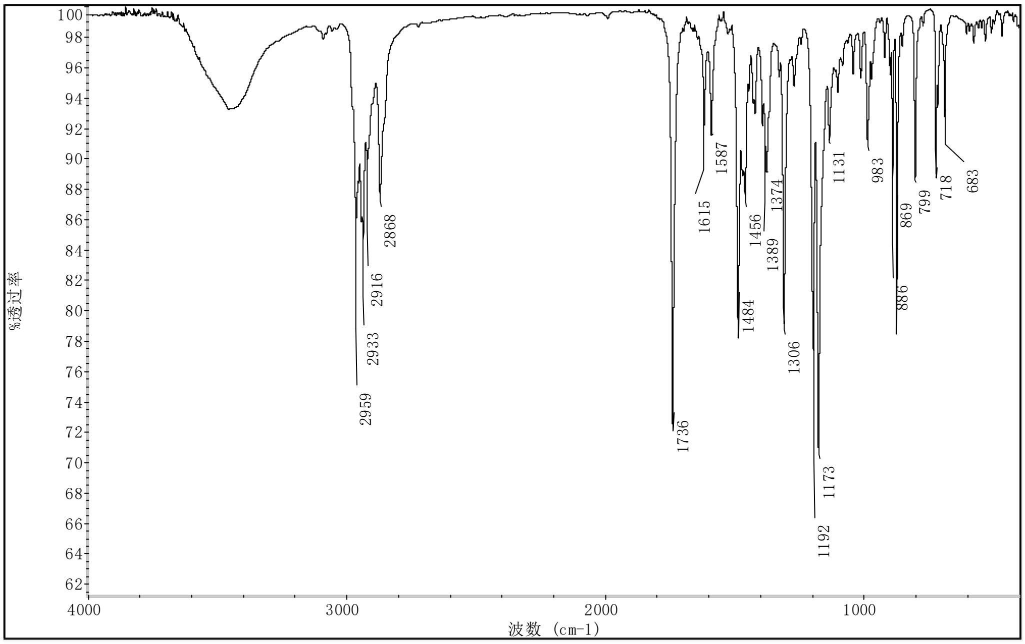

Figure S25. FT-IR spectrum of Compound **5g**(R=*m*-F Ph)

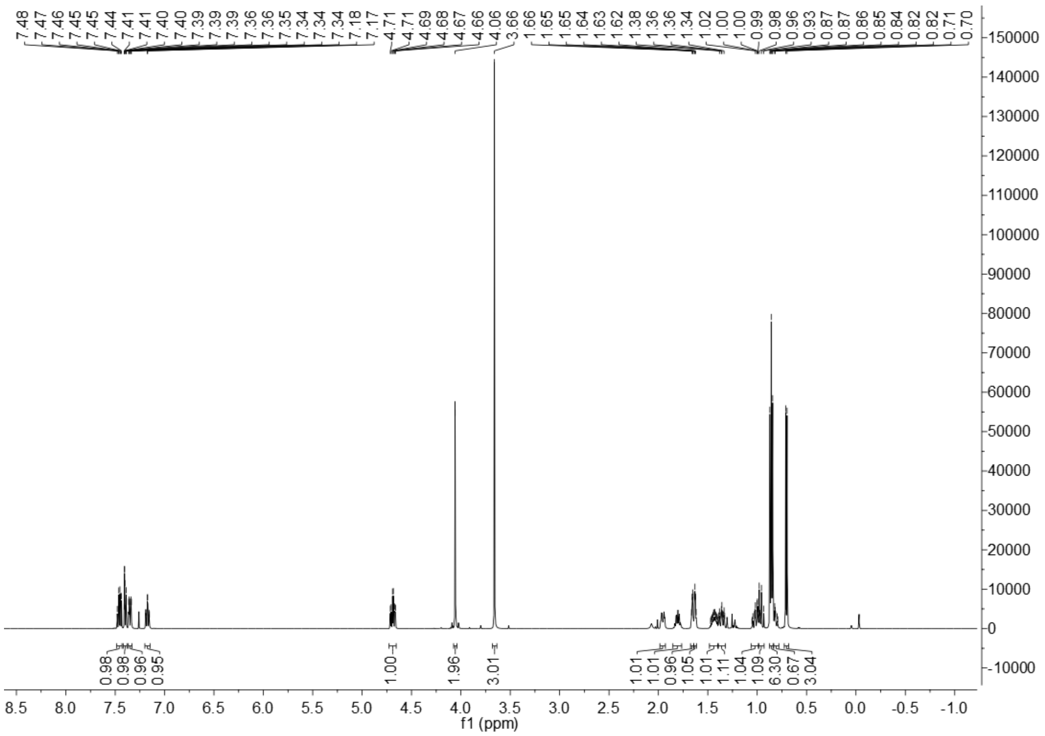

Figure S26. ^{1}H-NMR spectrum of Compound **5g**(R=*m*-F Ph)

in $CDCl_3$

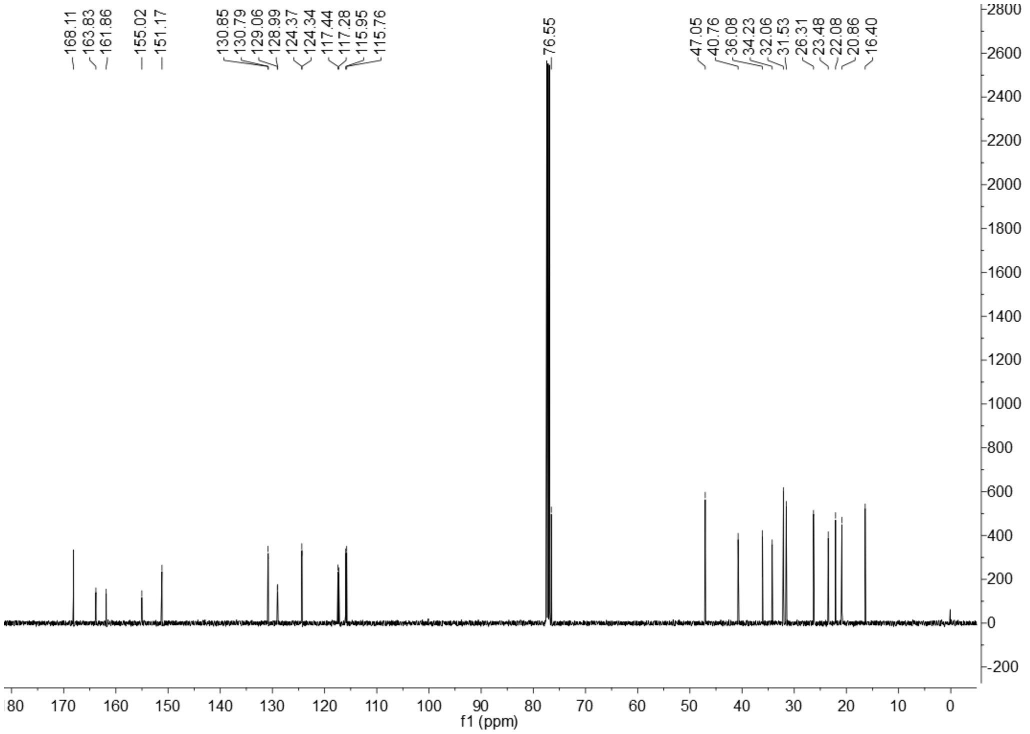

Figure S27. ^{13}C-NMR spectrum of Compound **5g**(R=*m*-F Ph) in $CDCl_3$.

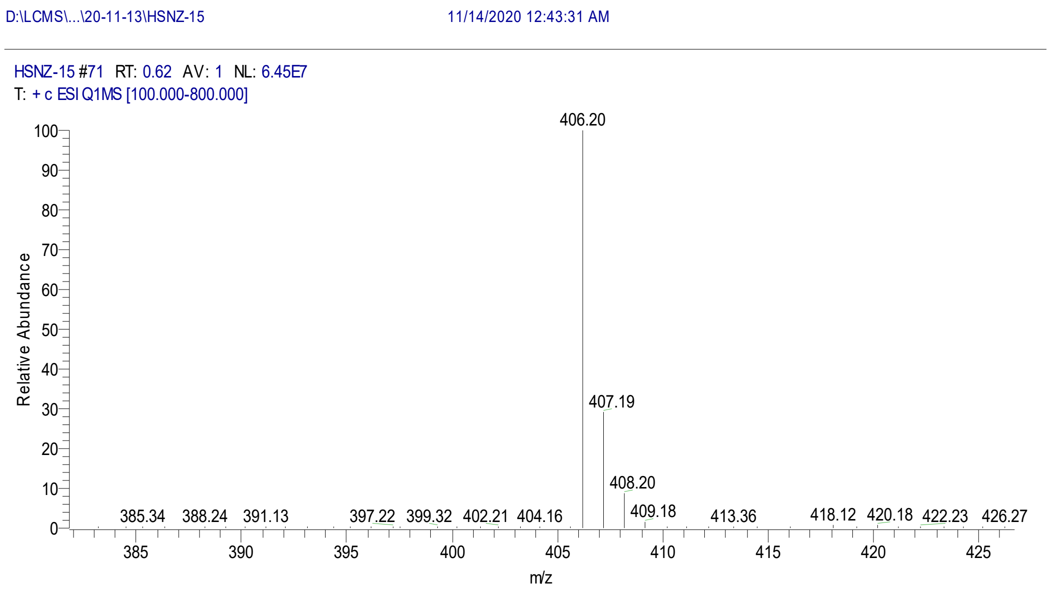

Figure S28. ESI-MS spectrum of Compound **5g**(R=*m*-F Ph).

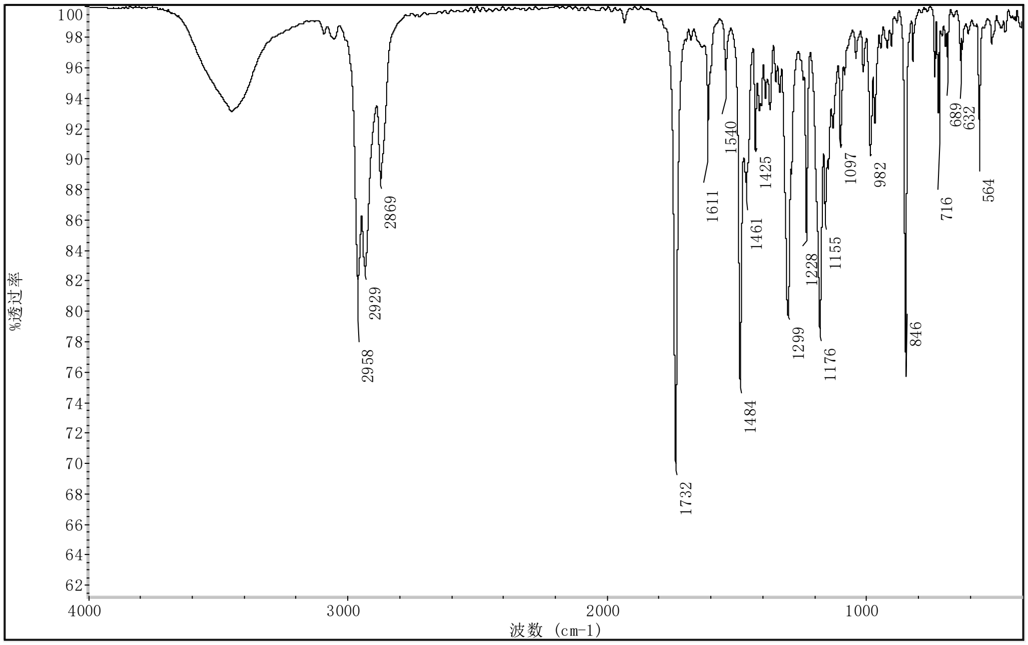

Figure S29. FT-IR spectrum of Compound **5h**(R=*p*-F Ph)

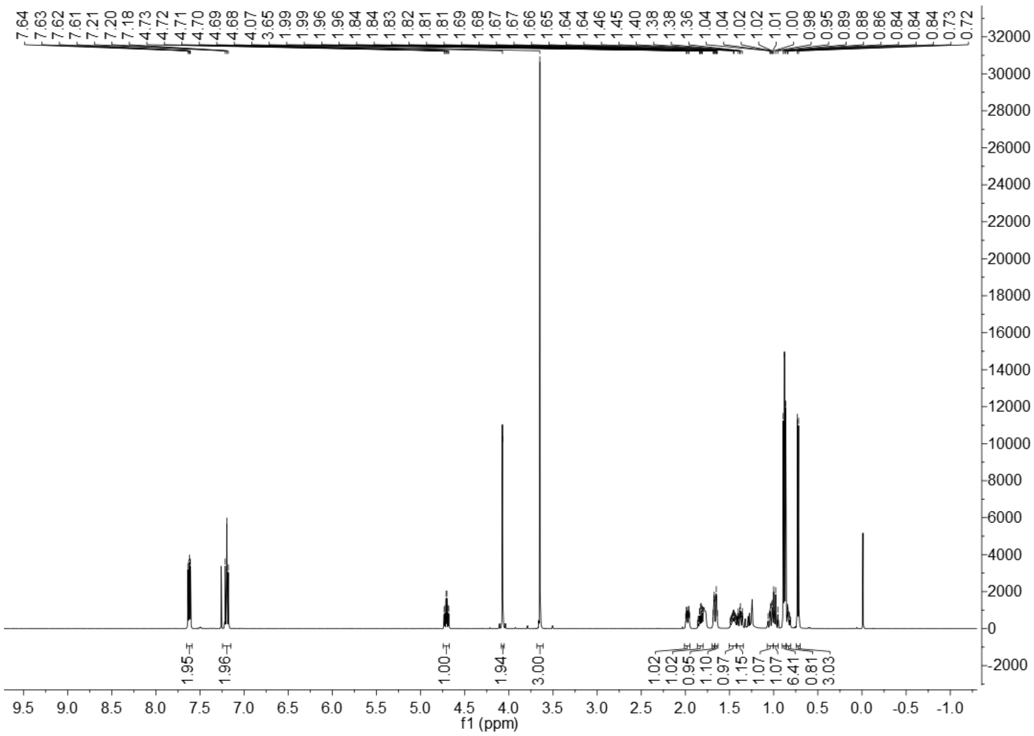

Figure S30. ^{1}H-NMR spectrum of Compound **5h**(R=*p*-F Ph)

in $CDCl_3$

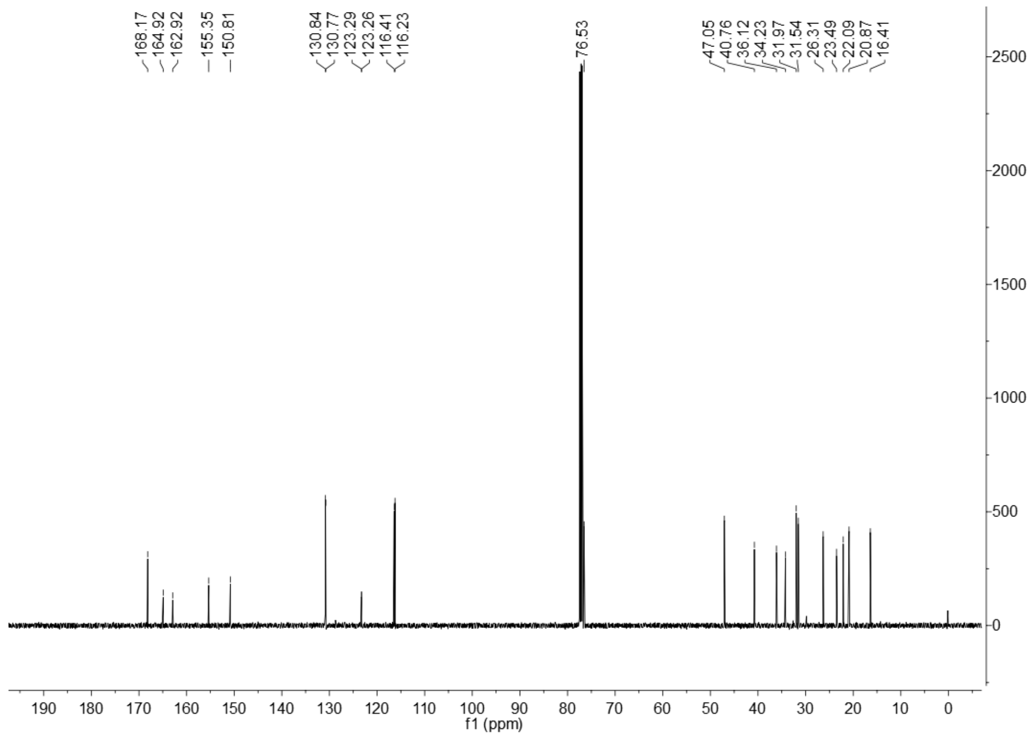

Figure S31. ^{13}C-NMR spectrum of Compound **5h**(R=*p*-F Ph) in $CDCl_3$.

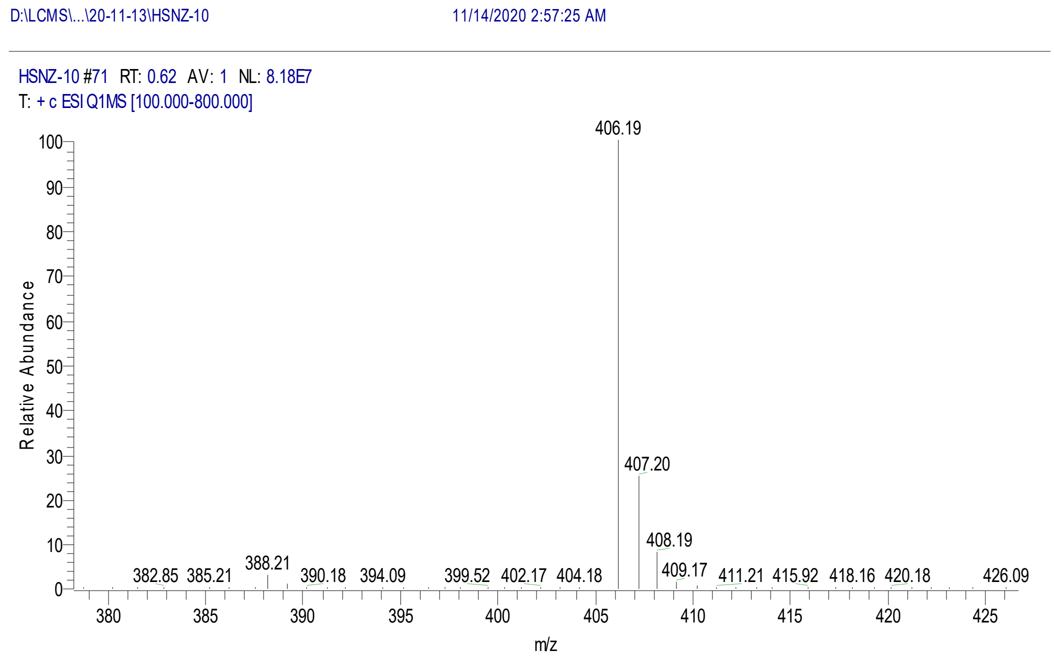

Figure S32. ESI-MS spectrum of Compound **5h**(R=*p*-F Ph).

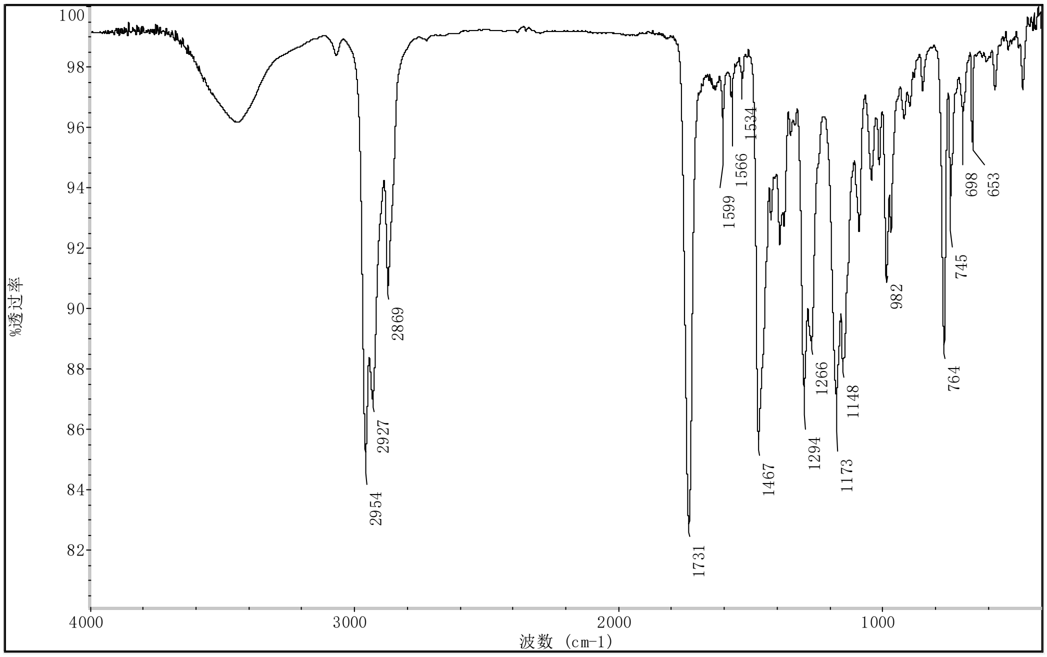

Figure S33. FT-IR spectrum of Compound **5i**(R=*o*-Cl Ph)

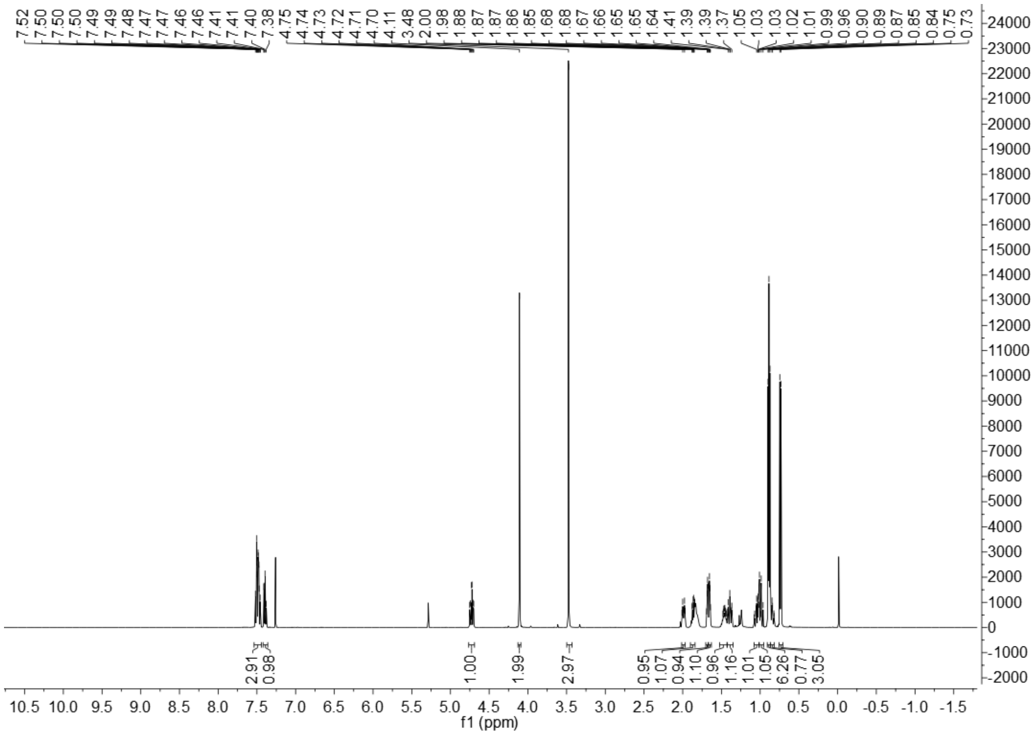

Figure S34. ^{1}H-NMR spectrum of Compound **5i**(R=*o*-Cl Ph) in $CDCl_3$

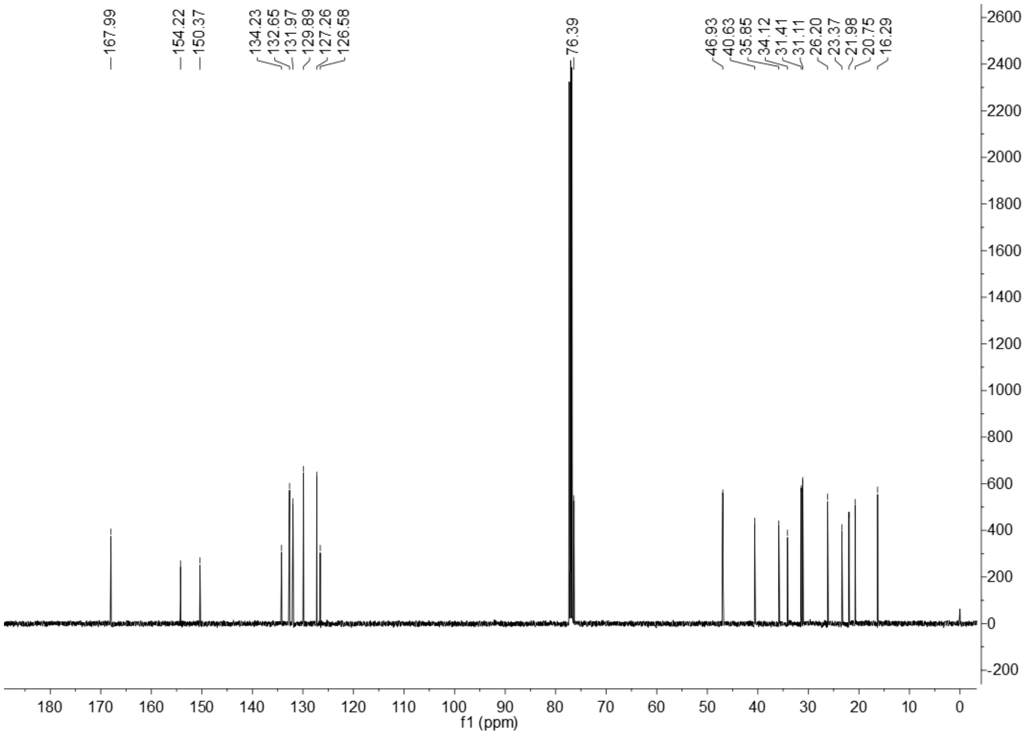

Figure S35 ^{13}C-NMR spectrum of Compound **5i**(R=*o*-Cl Ph) in $CDCl_3$.

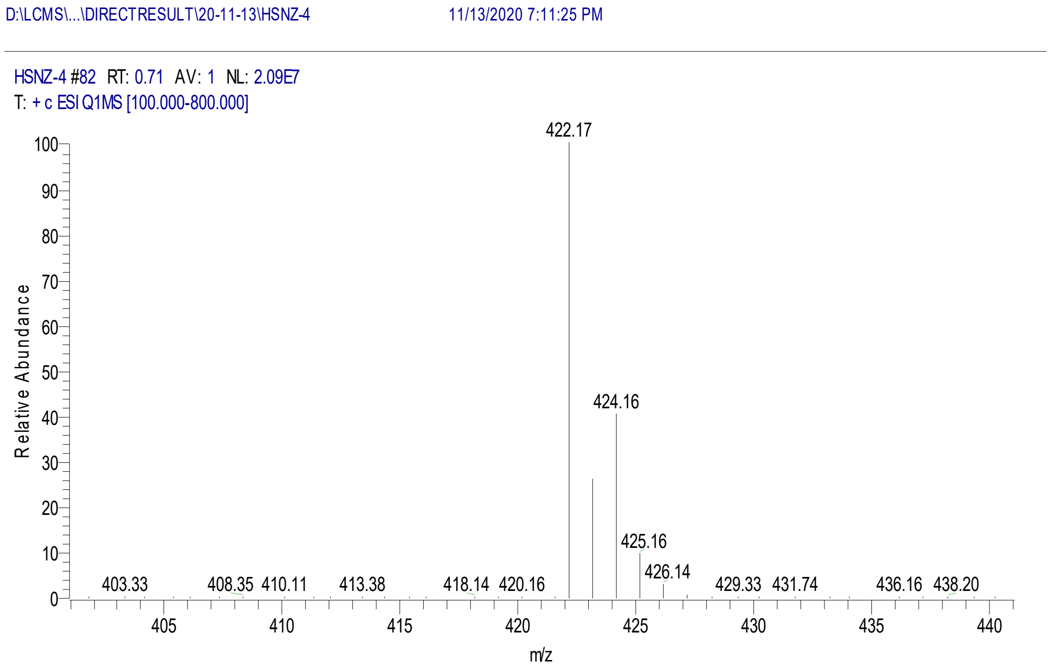

Figure S36. ESI-MS spectrum of Compound **5i**(R=*o*-Cl Ph).

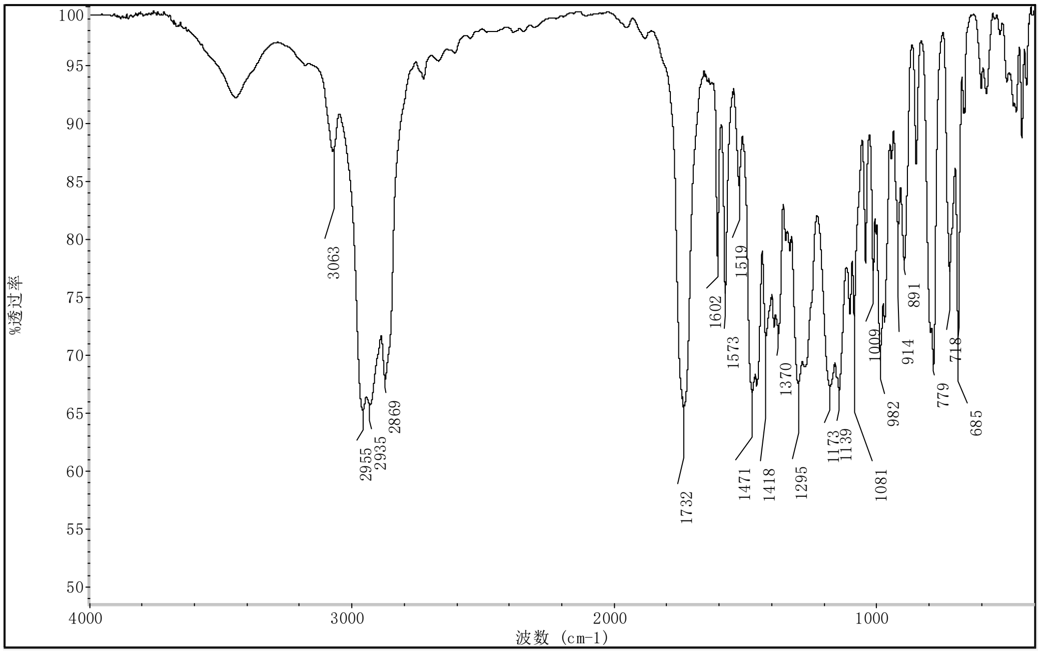

Figure S37. FT-IR spectrum of Compound **5j**(R=*m*-Cl Ph)

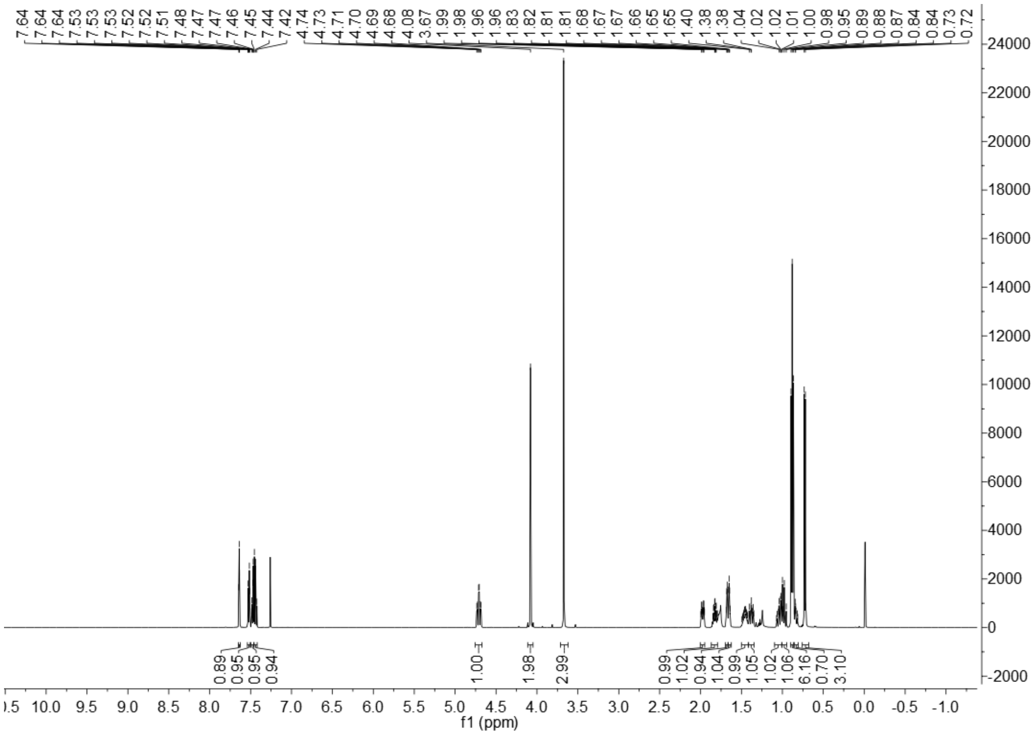

Figure S38. ^{1}H-NMR spectrum of Compound **5j**(R=*m*-Cl Ph)

in $CDCl_3$

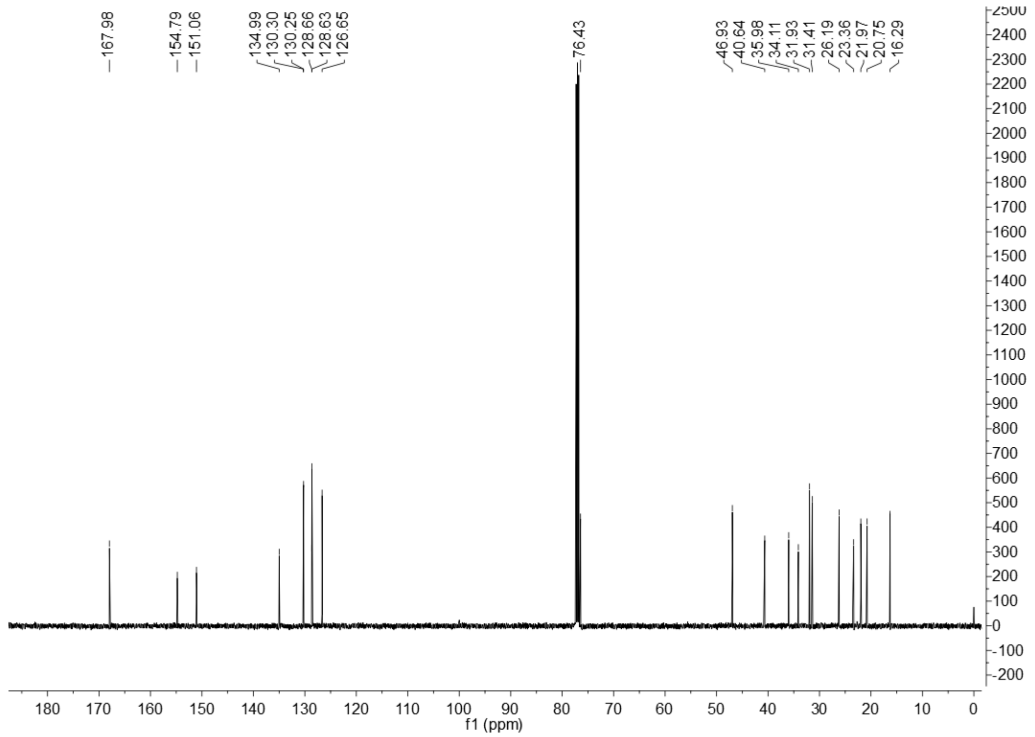

Figure S39 ^{13}C-NMR spectrum of Compound **5j**(R=*m*-Cl Ph) in $CDCl_3$.

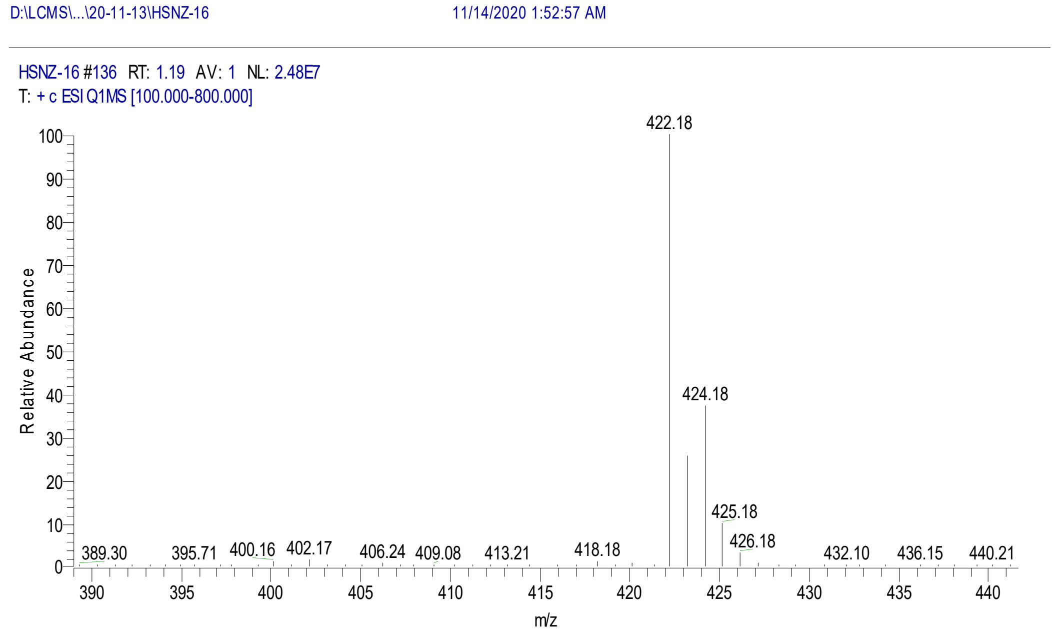

Figure S40. ESI-MS spectrum of Compound **5j**(R=*m*-Cl Ph).

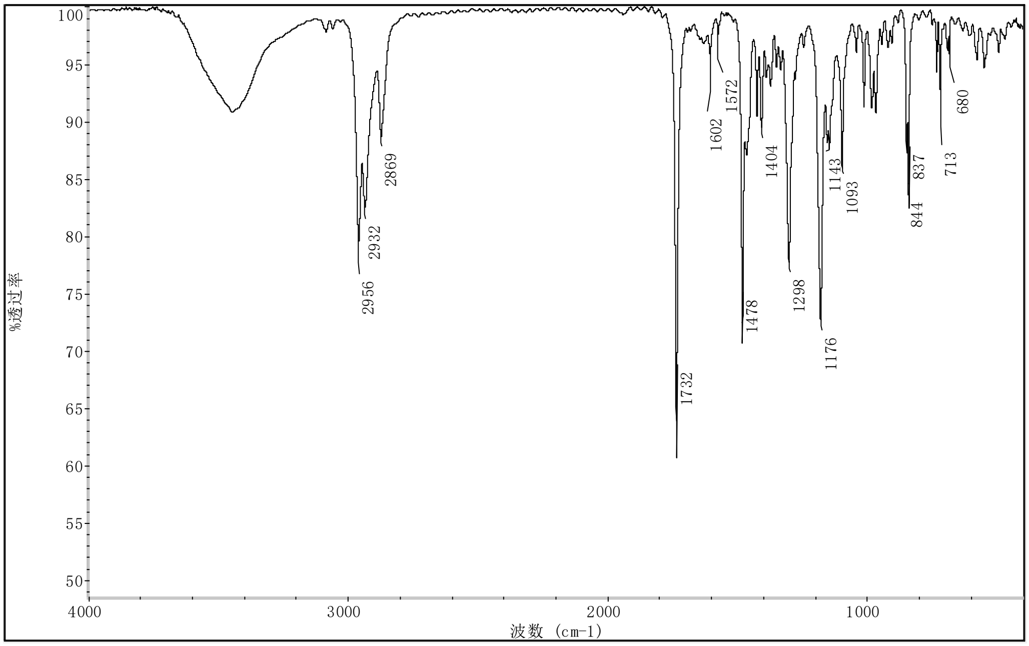

Figure S41. FT-IR spectrum of Compound **5k**(R=*p*-Cl Ph)

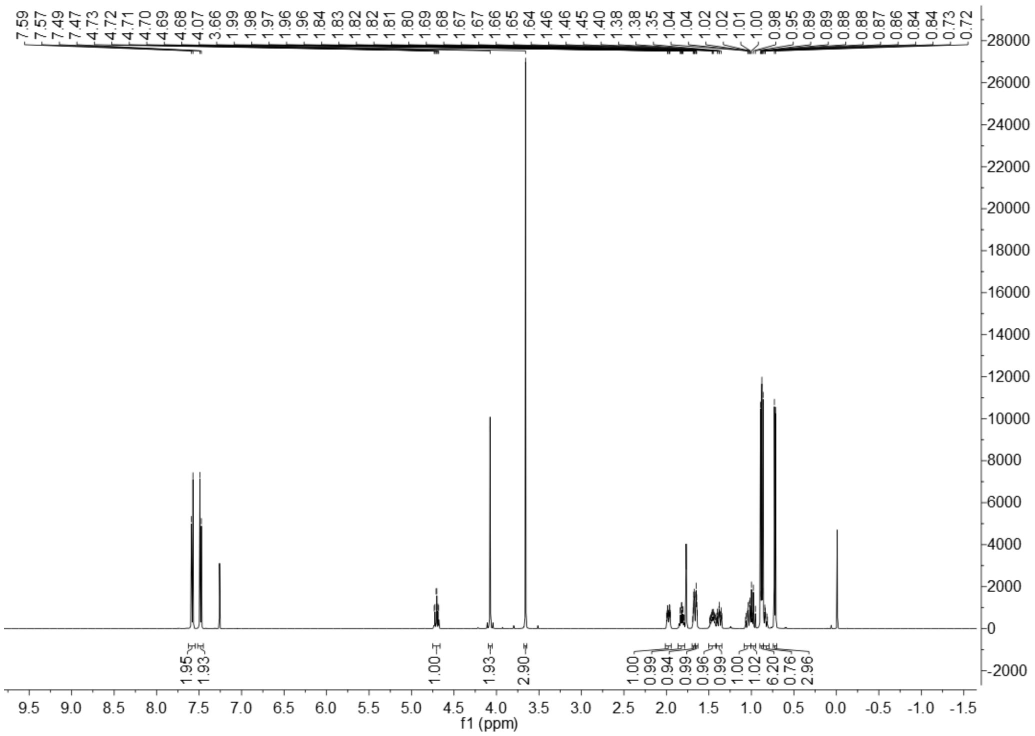

Figure S42. ^{1}H-NMR spectrum of Compound **5k**(R=*p*-Cl Ph) in $CDCl_3$

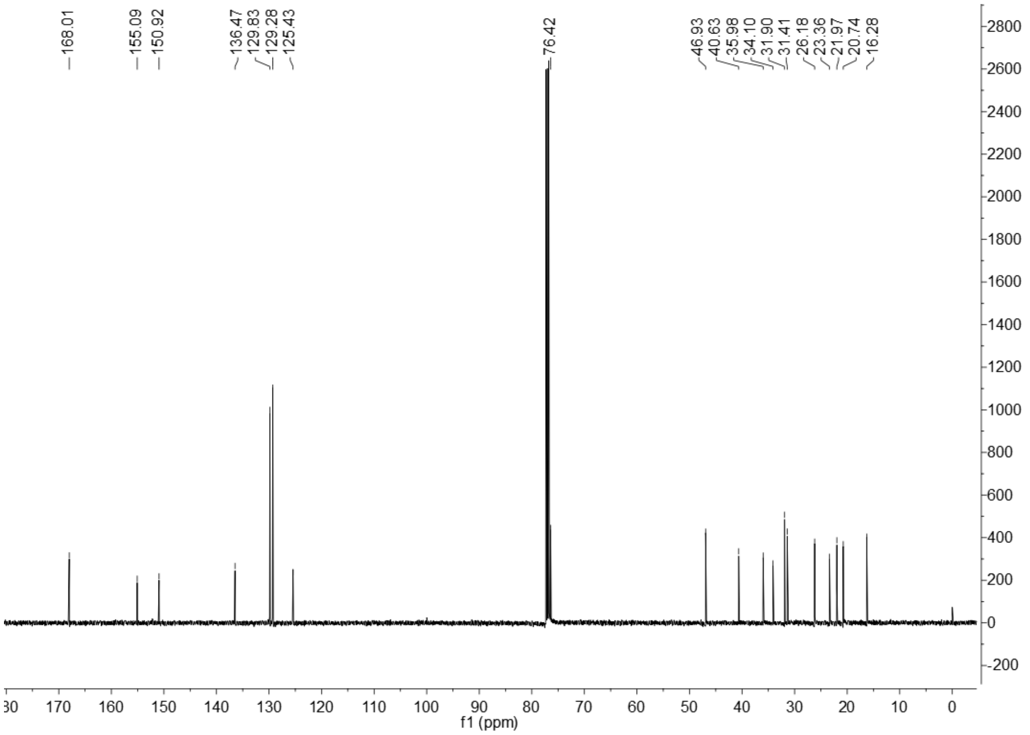

Figure S43 ^{13}C-NMR spectrum of Compound **5k**(R=*p*-Cl Ph) in $CDCl_3$.

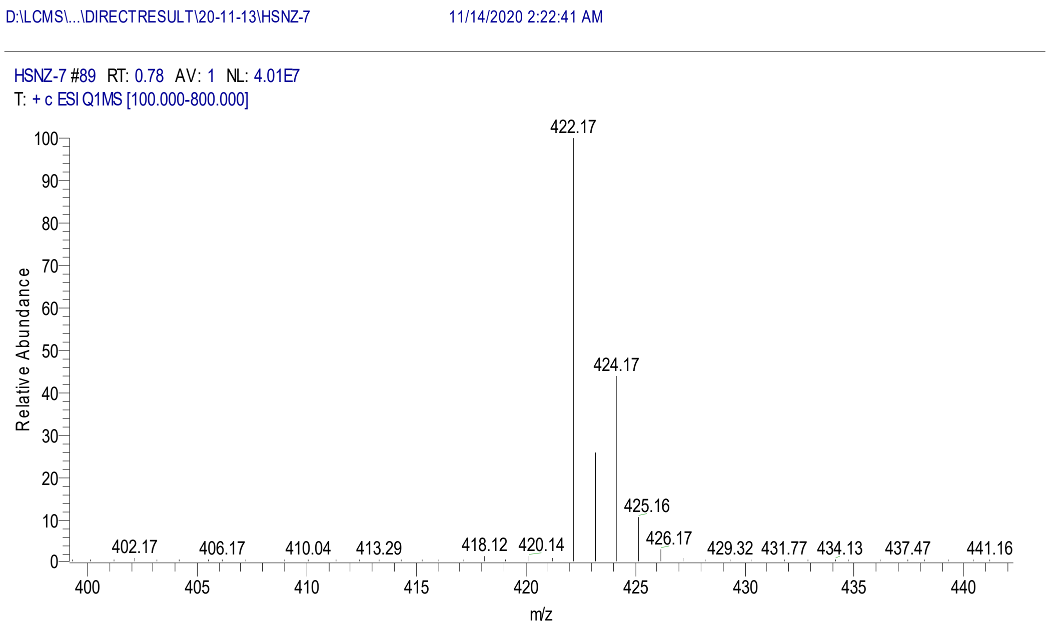

Figure S44. ESI-MS spectrum of Compound **5k**(R=*p*-Cl Ph).

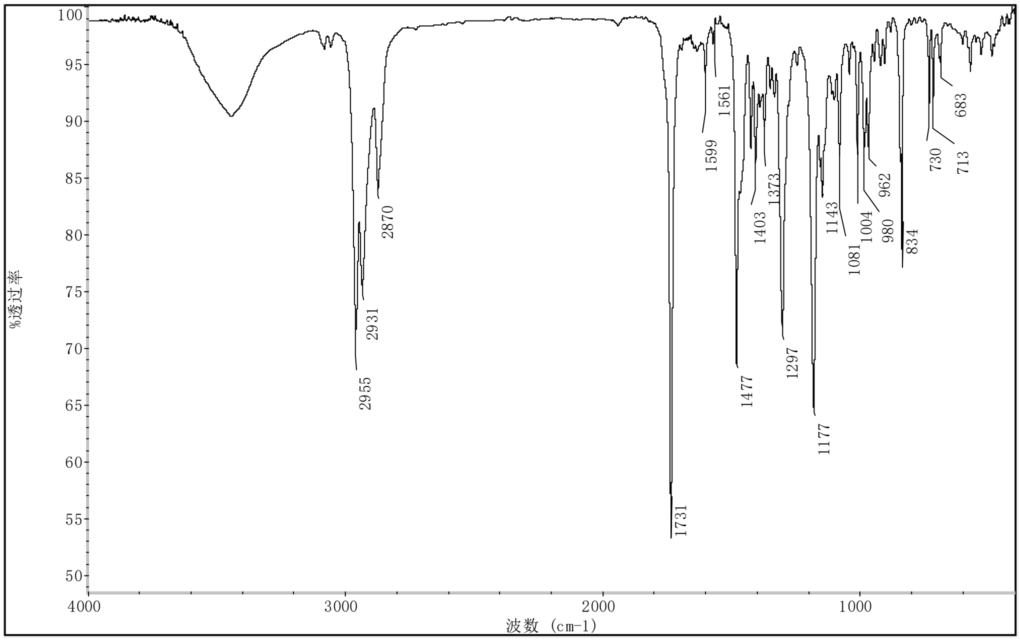

Figure S45. FT-IR spectrum of Compound **5l**(R=*p*-Br Ph)

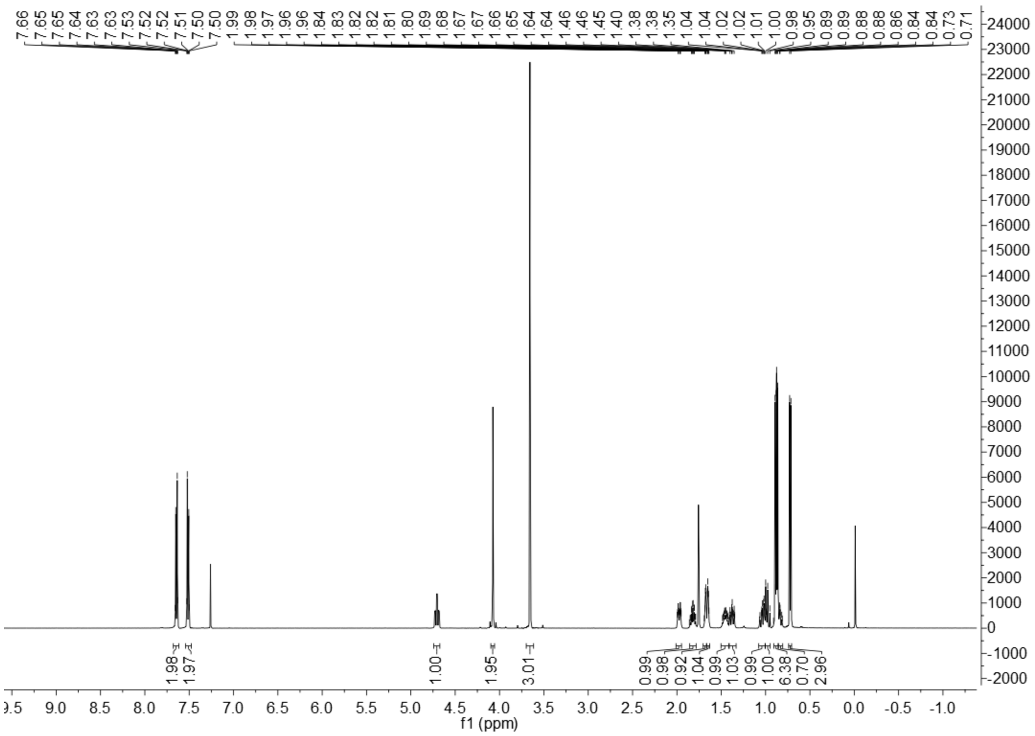

Figure S46. ^{1}H-NMR spectrum of Compound **5l**(R=*p*-Br Ph) in $CDCl_3$

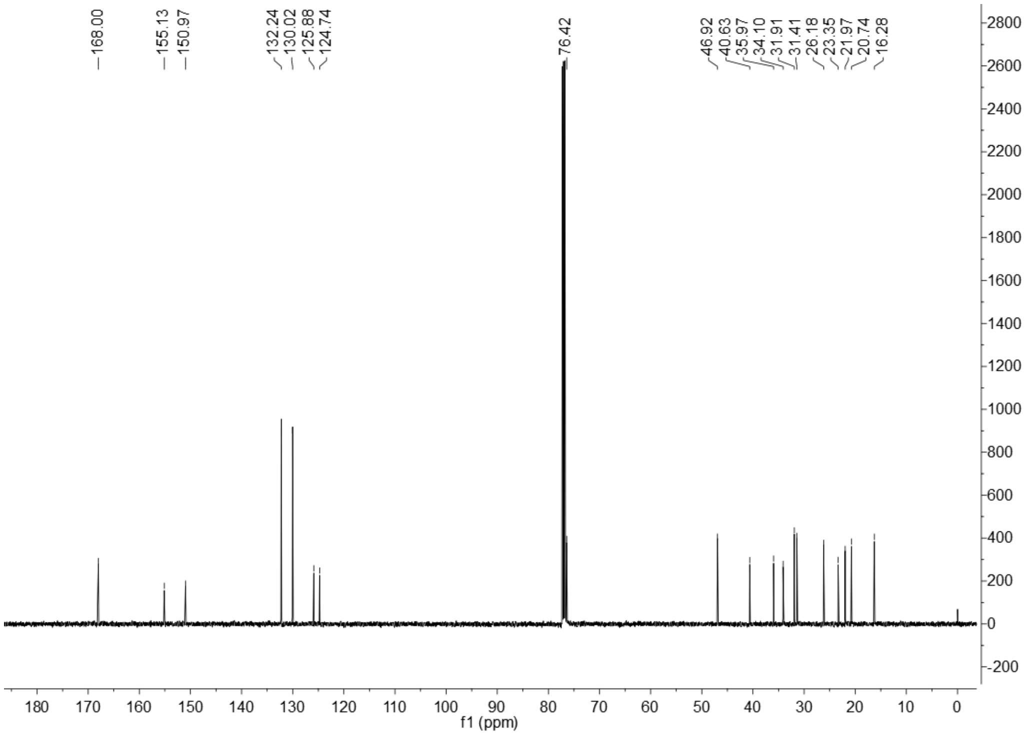

Figure S47 ^{13}C-NMR spectrum of Compound **5l**(R=*p*-Br Ph) in $CDCl_3$.

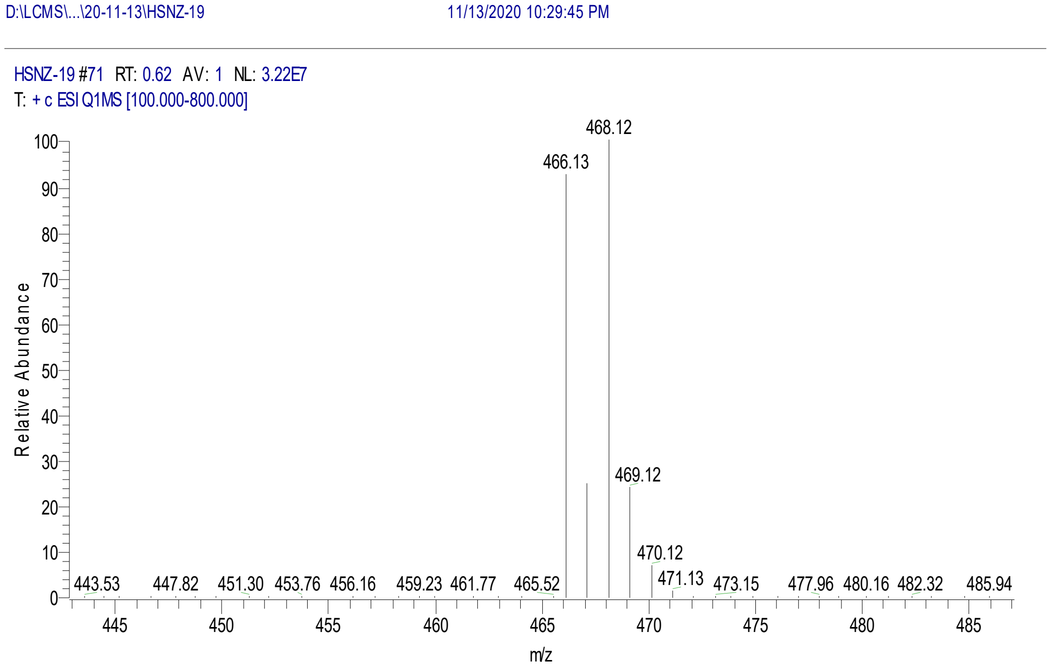

Figure S48. ESI-MS spectrum of Compound **5l**(R=*p*-Br Ph).

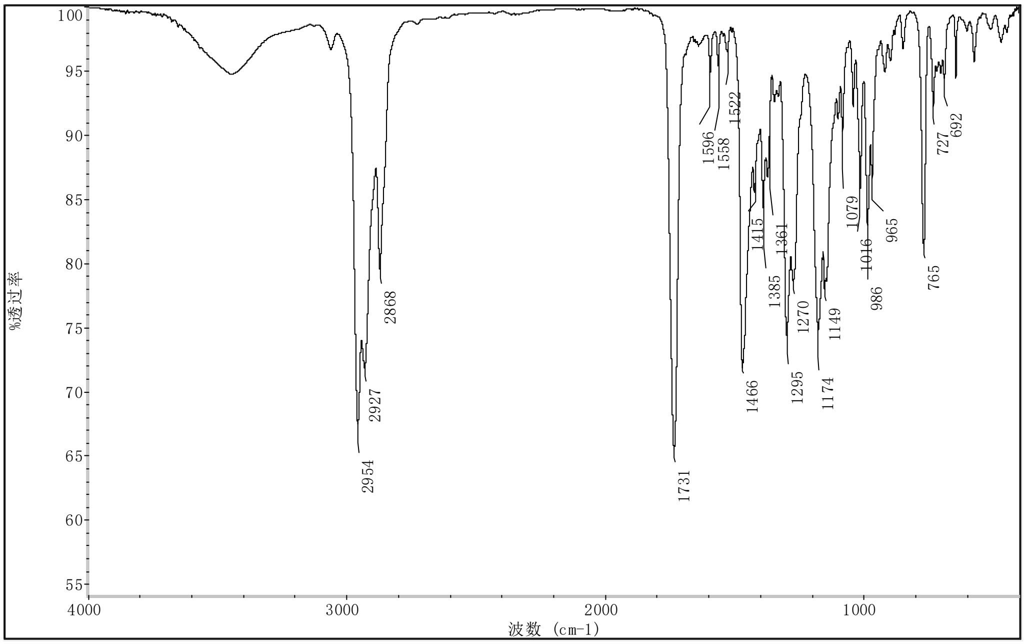

Figure S49. FT-IR spectrum of Compound **5m**(R=*o*-I Ph)

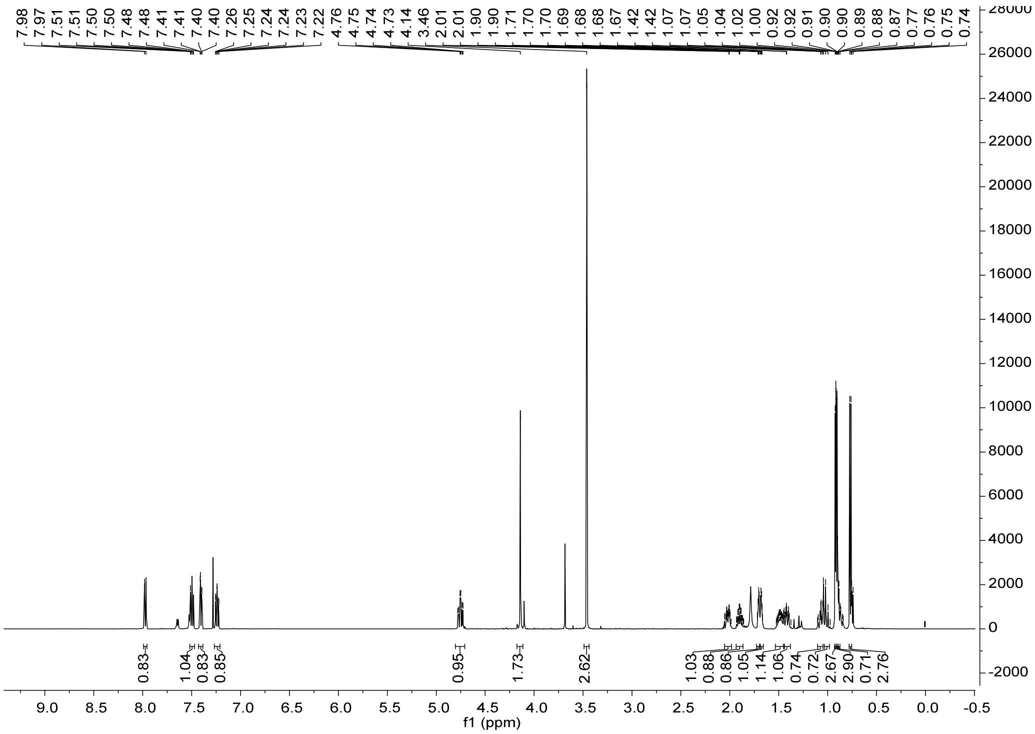

Figure S50. ^{1}H-NMR spectrum of Compound **5m**(R=*o*-I Ph) in $CDCl_3$

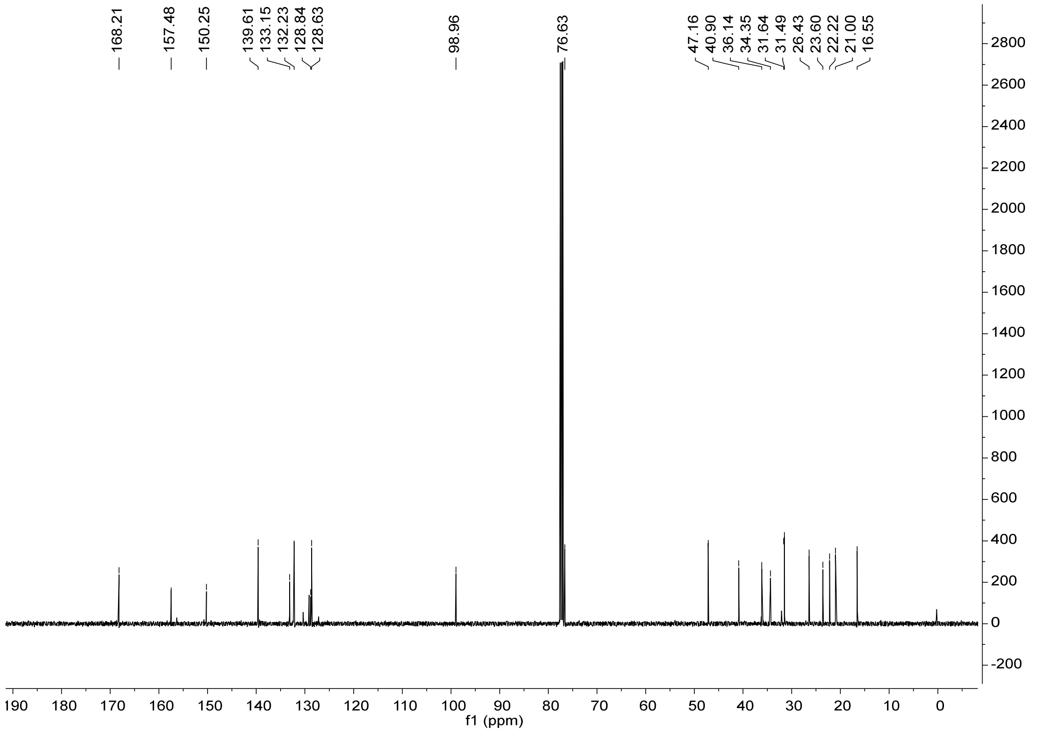

Figure S55 ^{13}C-NMR spectrum of Compound **5m**(R=*o*-I Ph) in $CDCl_3$.

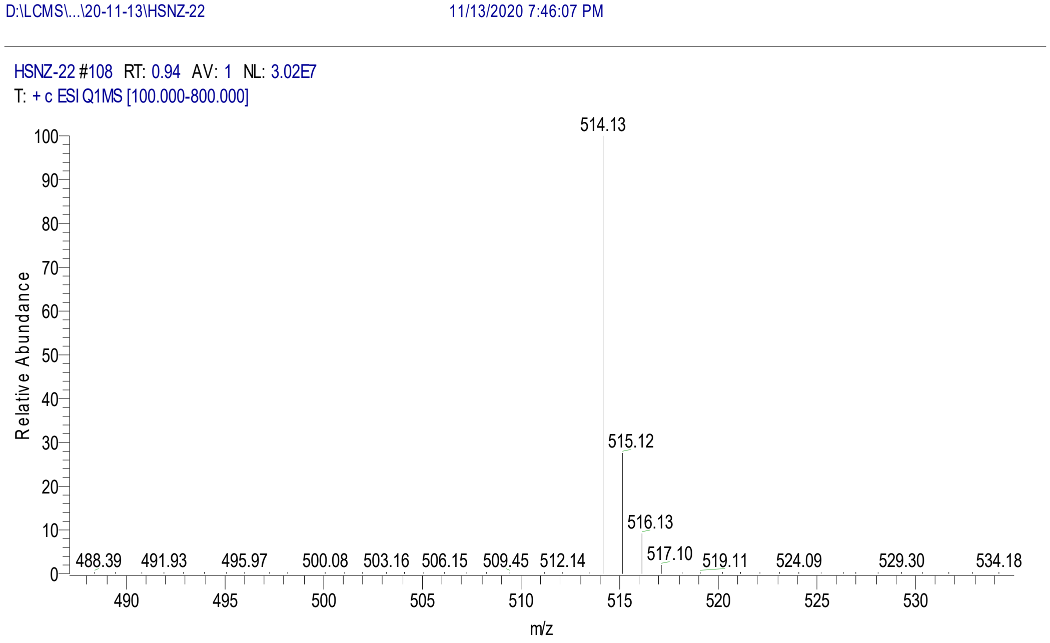

Figure S52. ESI-MS spectrum of Compound **5m**(R=*o*-I Ph).

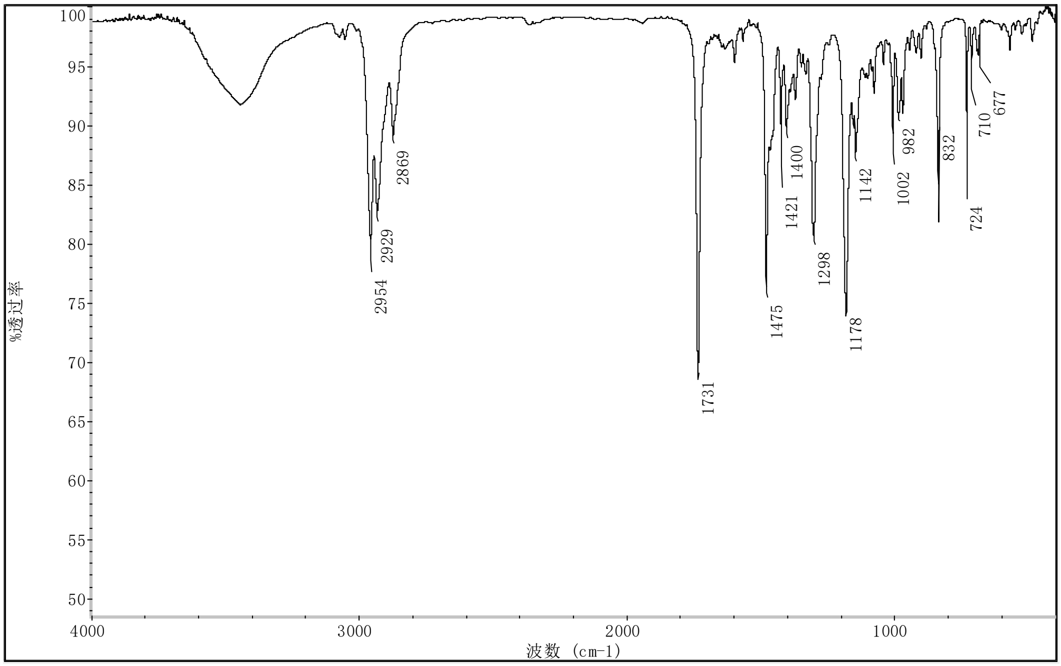

Figure S53. FT-IR spectrum of Compound **5n**(R=*p*-I Ph)

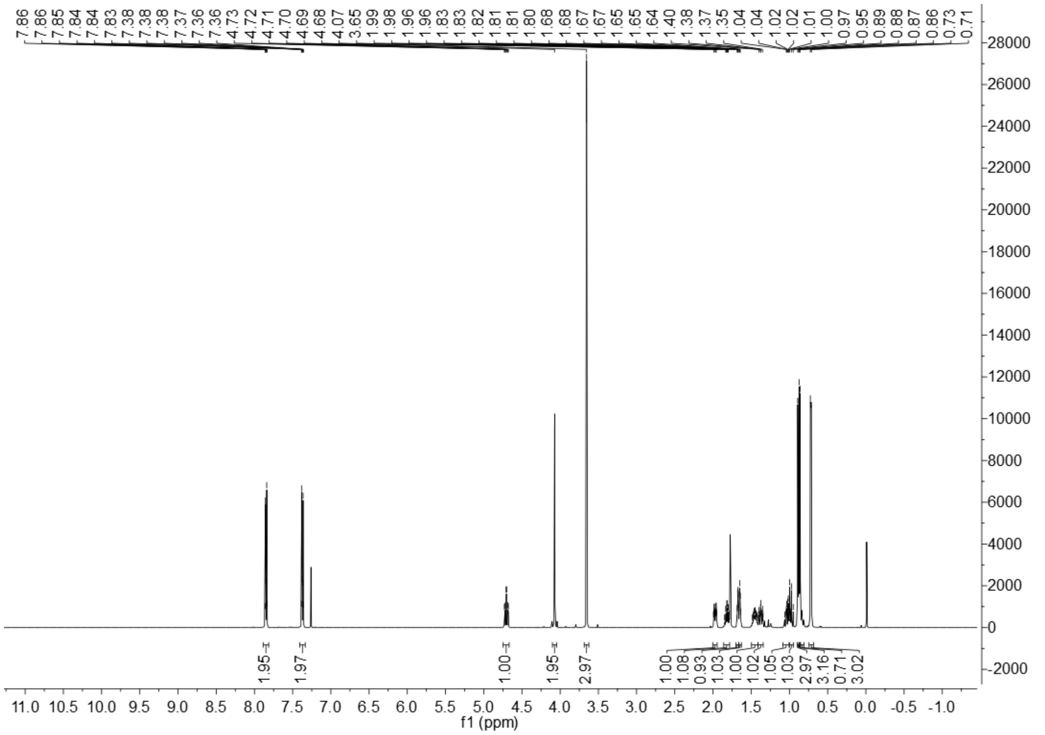

Figure S54. ^{1}H-NMR spectrum of Compound **5n**(R=*p*-I Ph) in $CDCl_3$

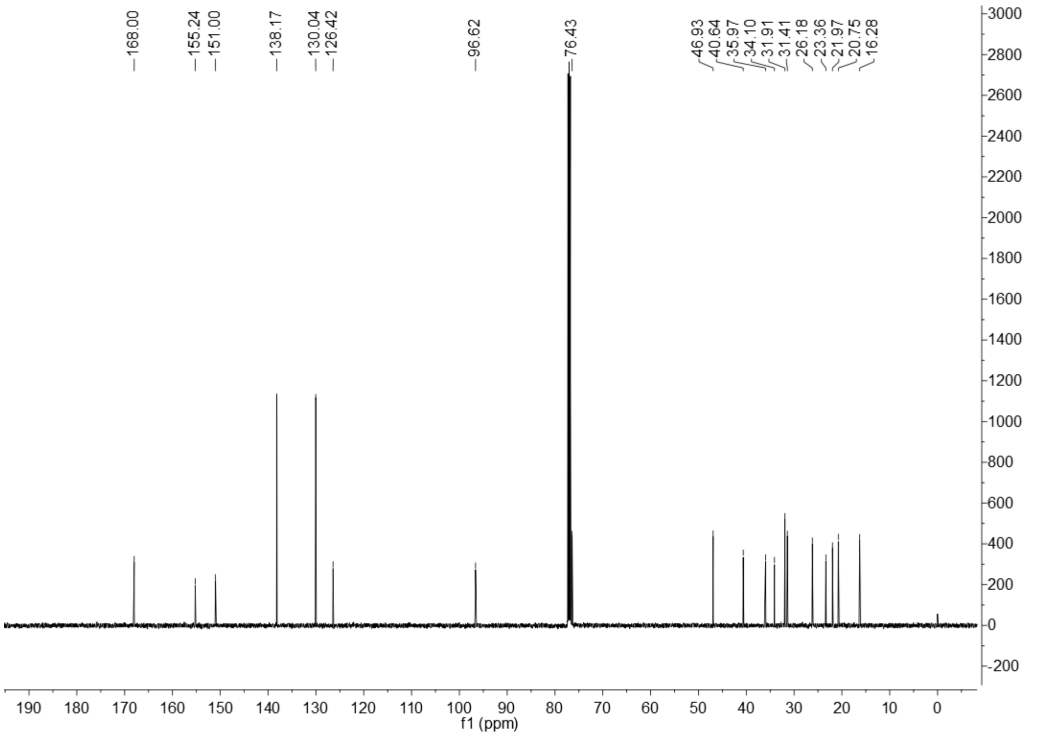

Figure S55 ^{13}C-NMR spectrum of Compound **5n**(R=*p*-I Ph) in $CDCl_3$.

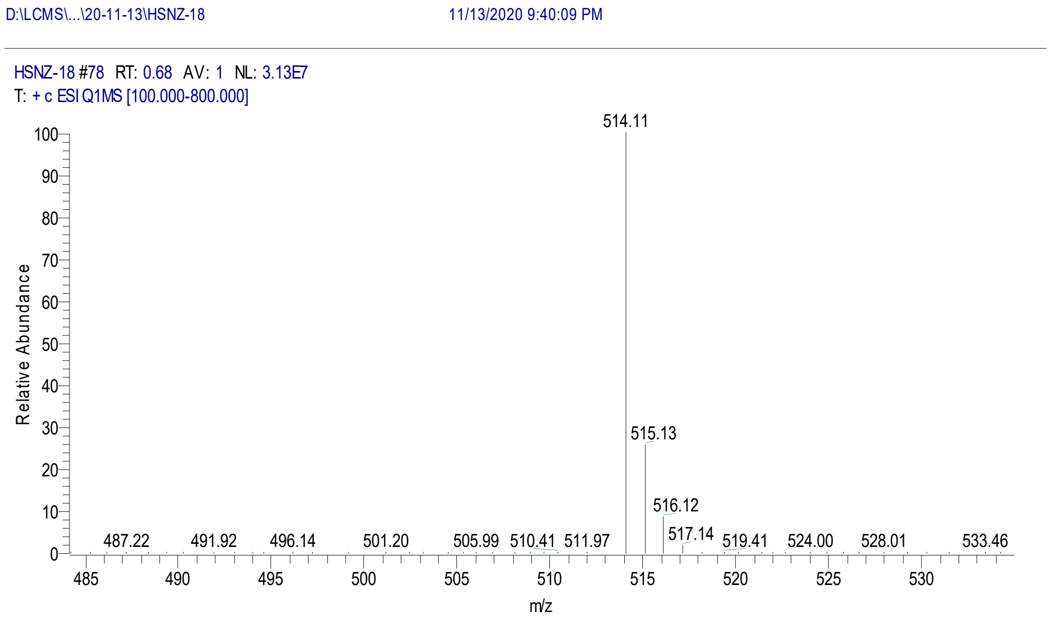

Figure S56. ESI-MS spectrum of Compound **5n**(R=*p*-I Ph).

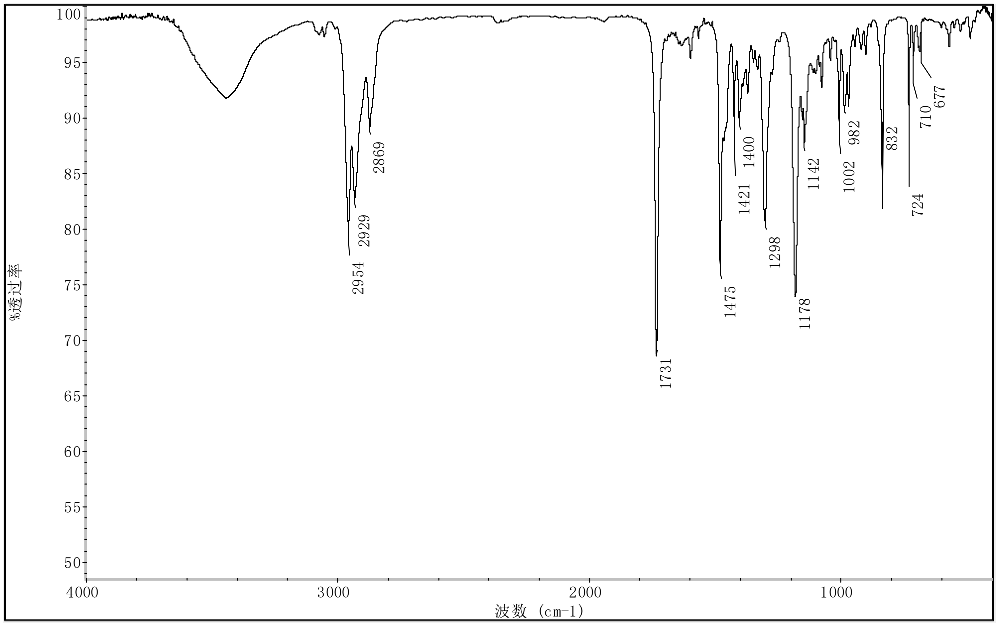

Figure S57. FT-IR spectrum of Compound **5o**(R=*o*-CF_3 Ph)

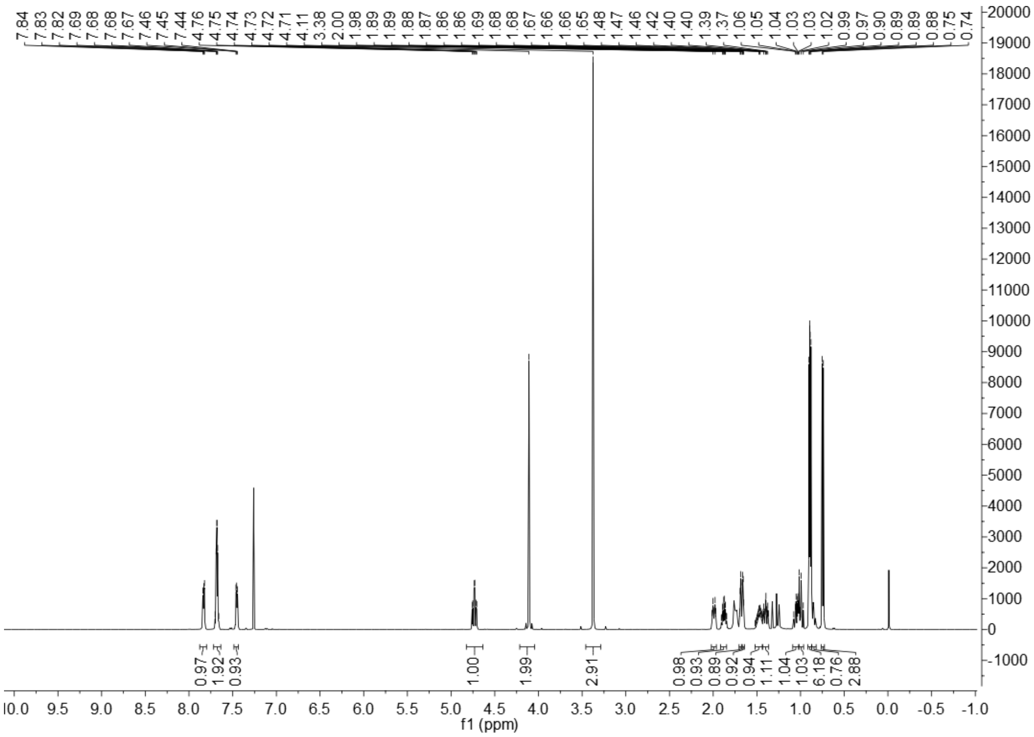

Figure S58. ^{1}H-NMR spectrum of Compound **5o**(R=*o*-CF_3 Ph) in $CDCl_3$

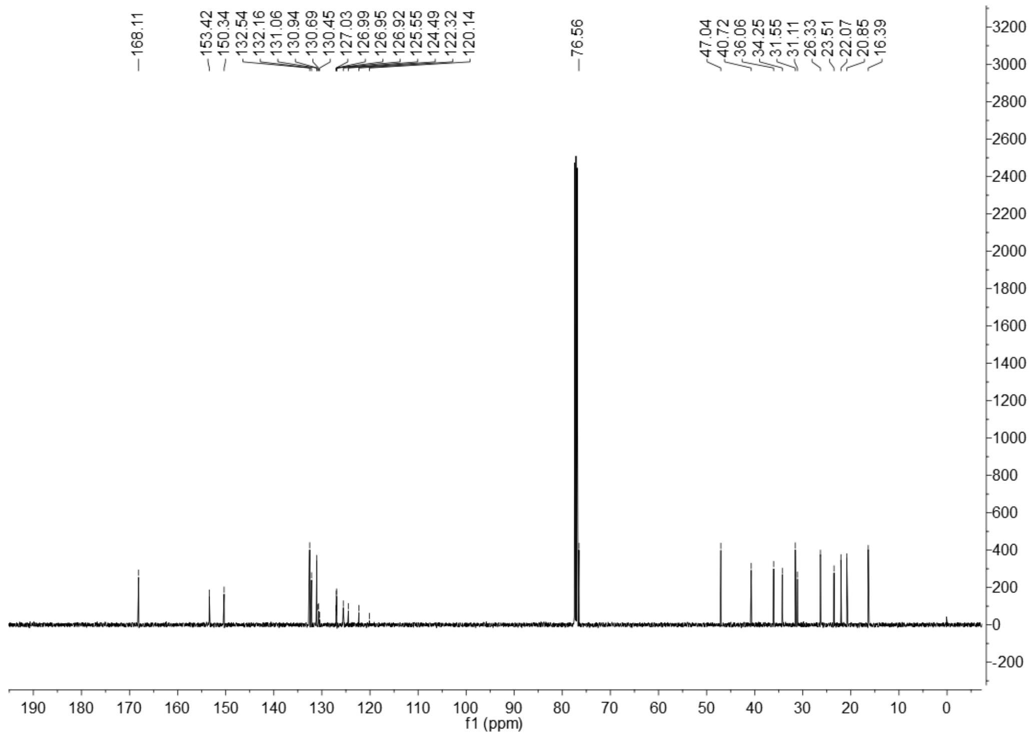

Figure S59 ^{13}C-NMR spectrum of Compound **5o**(R=*o*-CF_3 Ph) in $CDCl_3$.

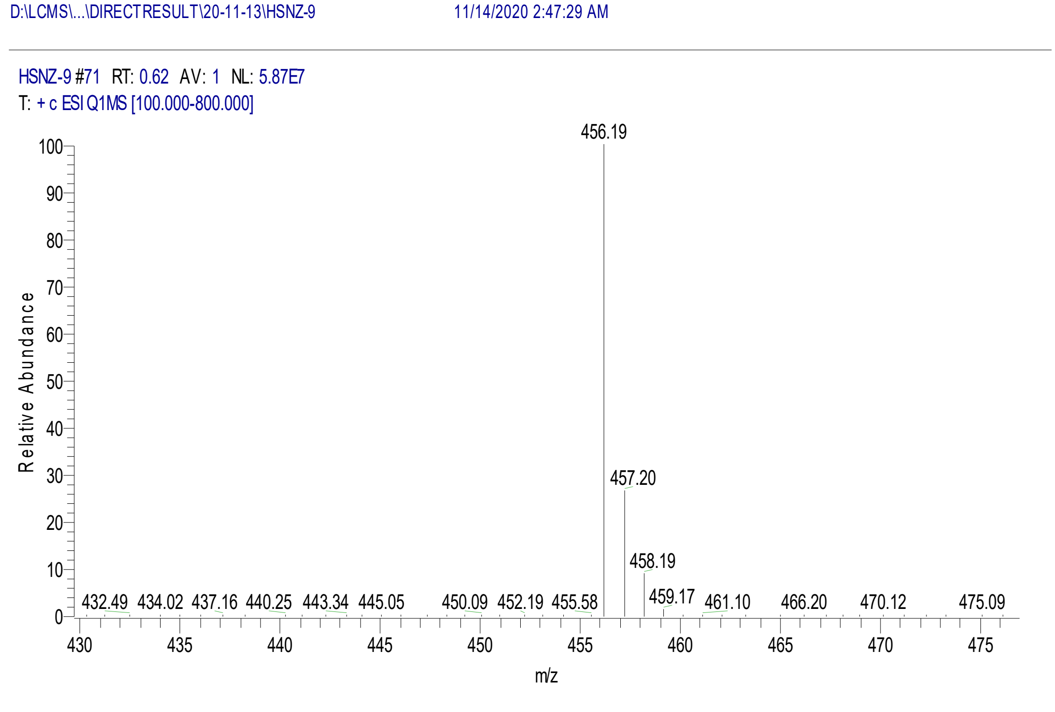

Figure S60. ESI-MS spectrum of Compound **5o**(R=*o*-CF_3 Ph).

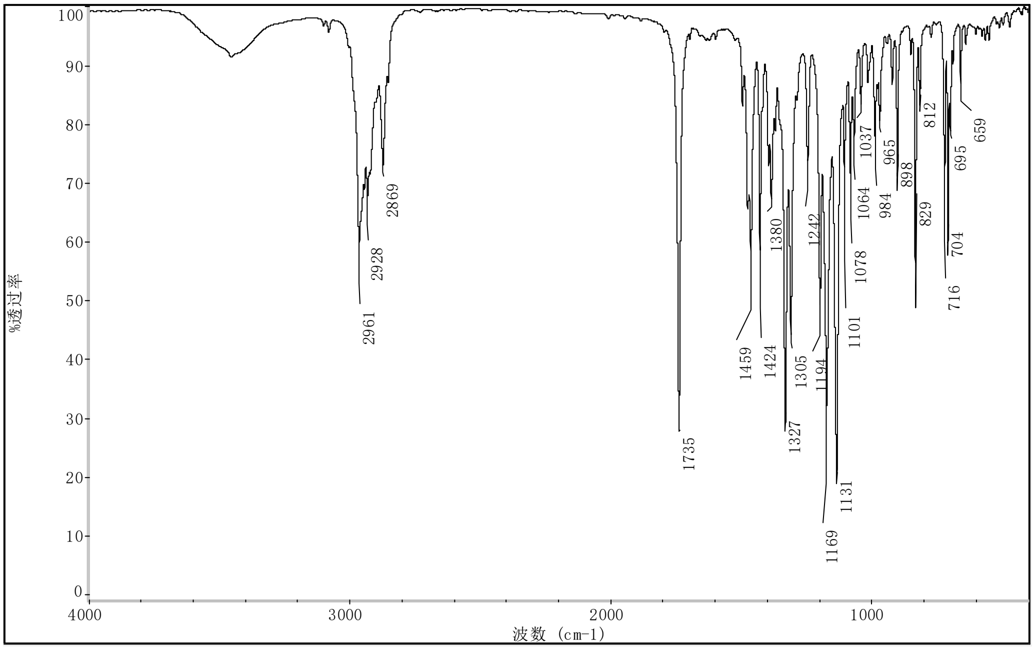

Figure S61. FT-IR spectrum of Compound **5p**(R=*m*-CF_3 Ph)

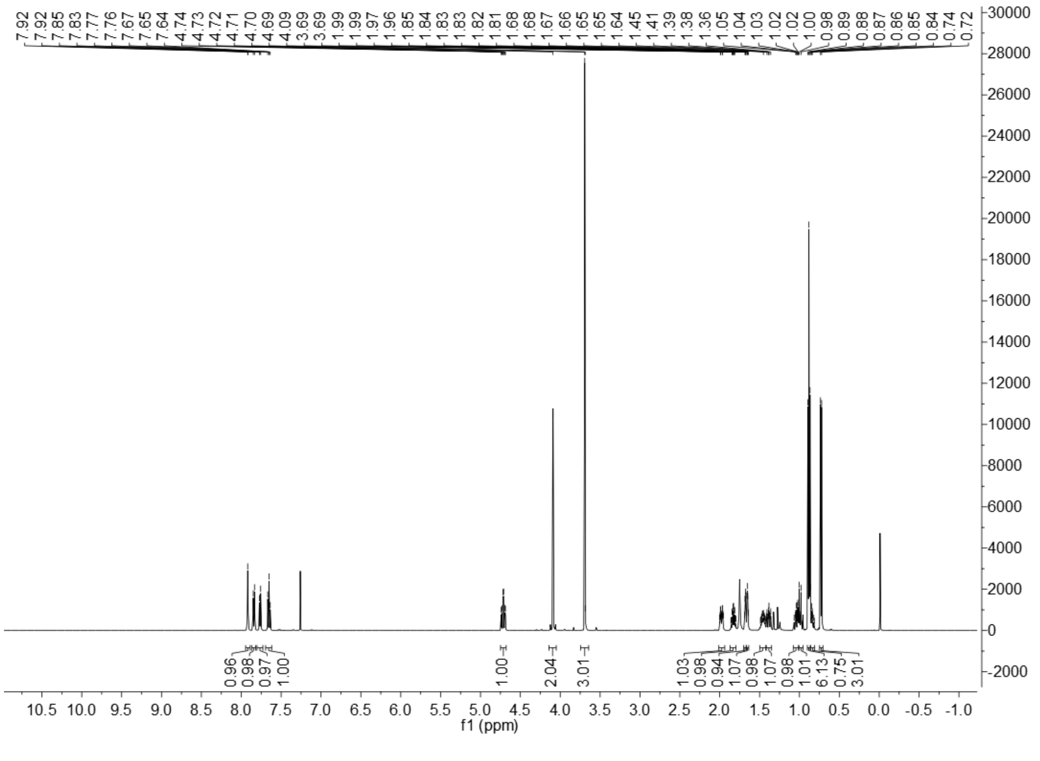

Figure S62. ^{1}H-NMR spectrum of Compound **5p**(R=*m*-CF_3 Ph) in $CDCl_3$

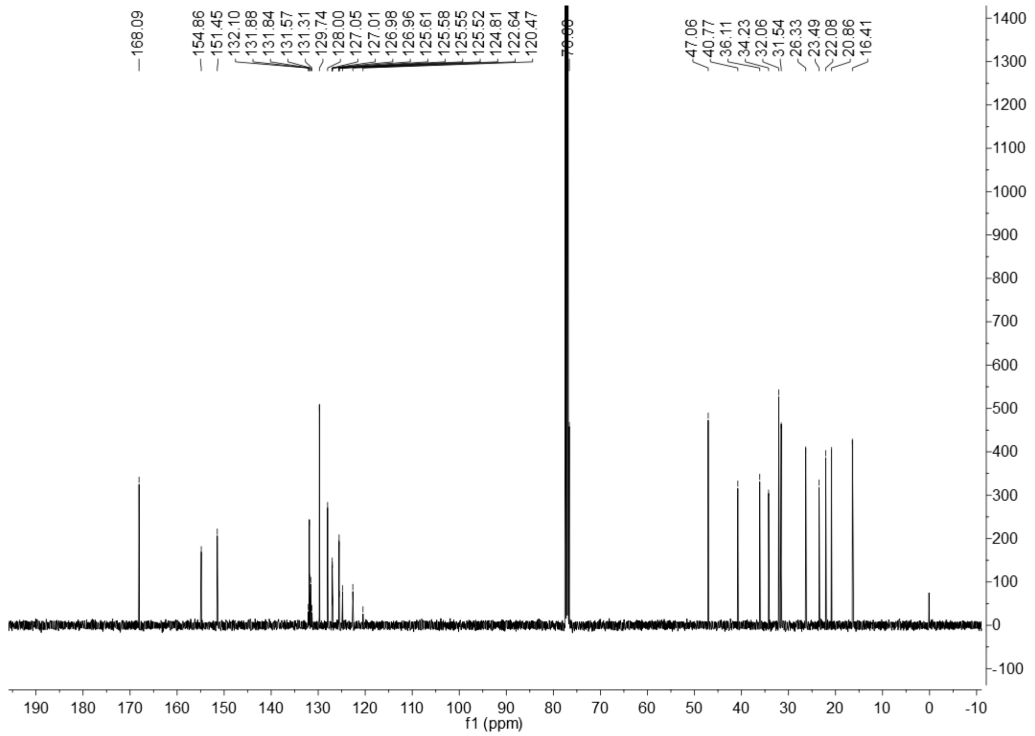

Figure S63 ^{13}C-NMR spectrum of Compound **5p**(R=*m*-CF_3 Ph) in $CDCl_3$.

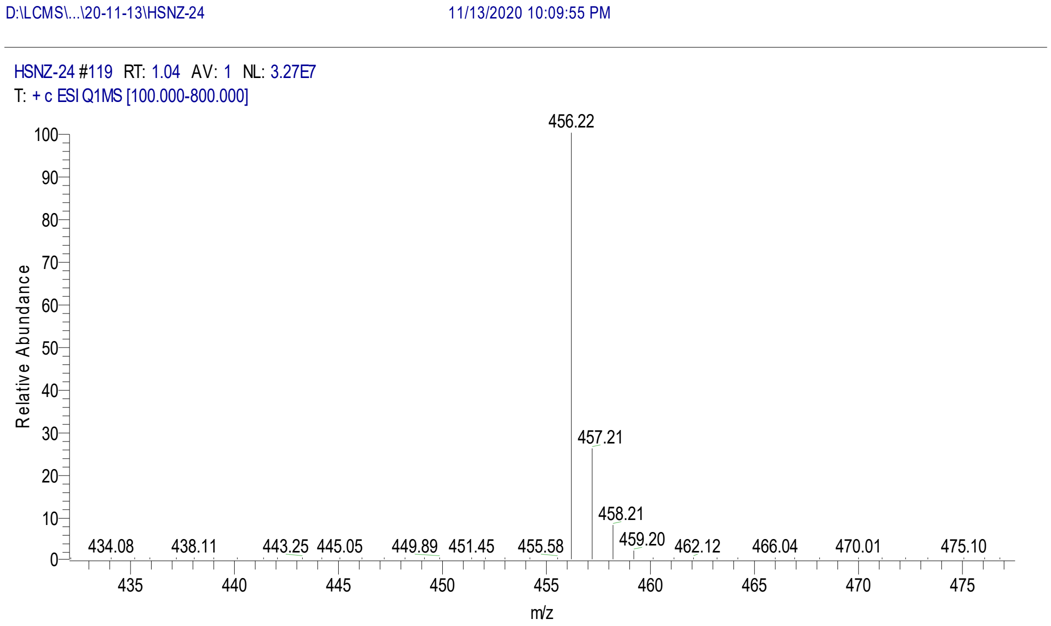

Figure S64. ESI-MS spectrum of Compound **5p**(R=*m*-CF_3 Ph).

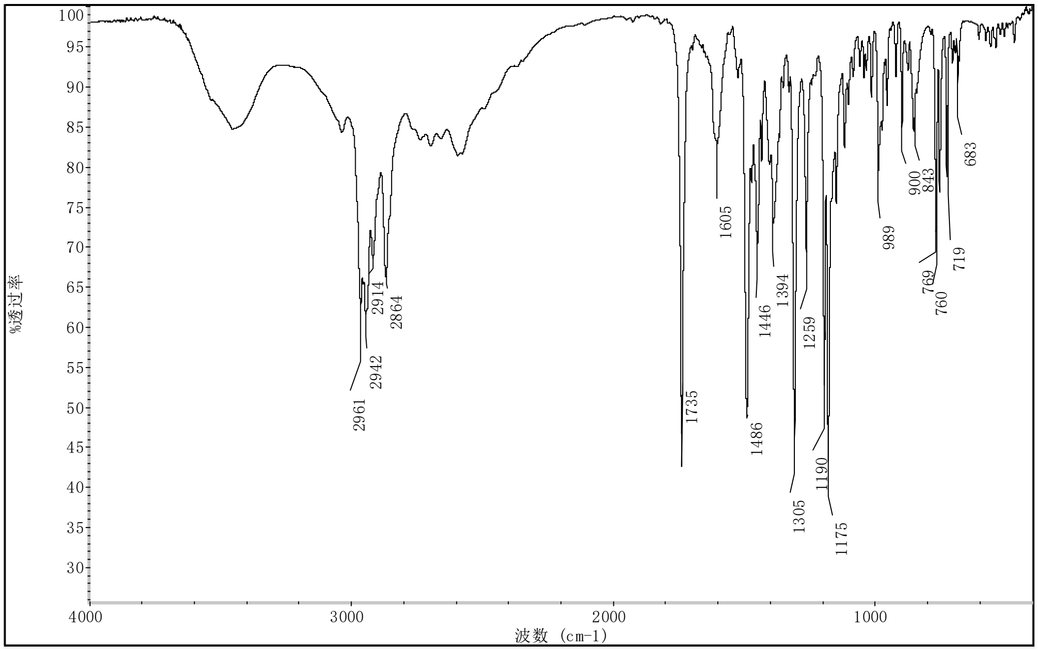

Figure S65. FT-IR spectrum of Compound **5q**(R=*o*-OH Ph)

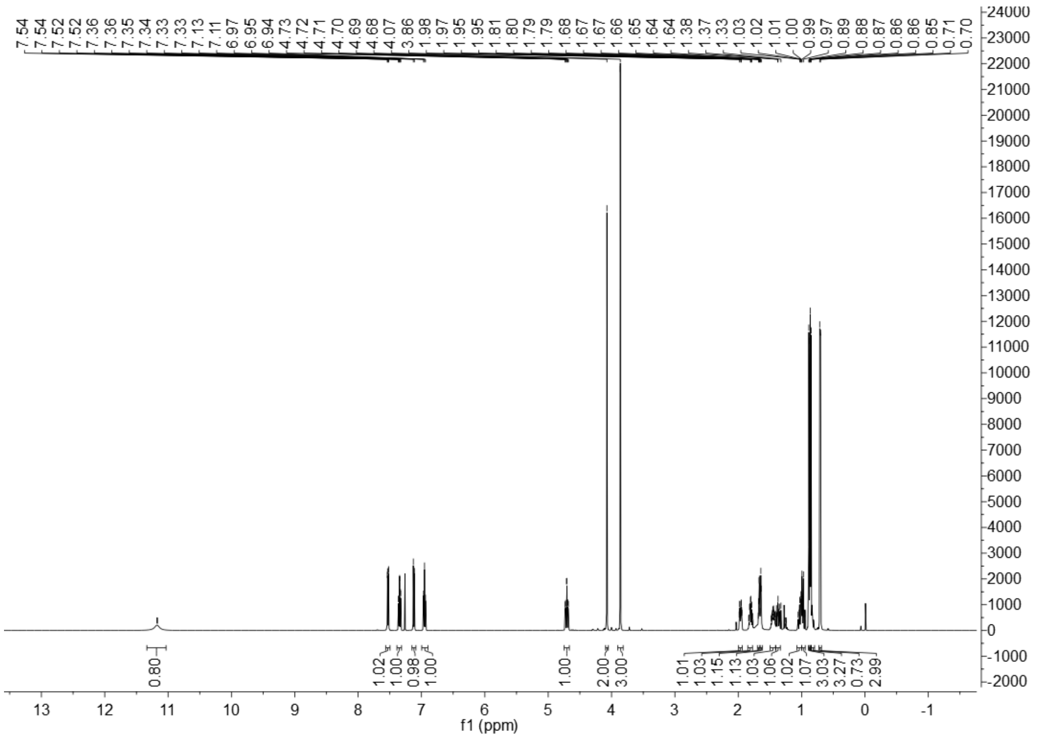

Figure S66. ^{1}H-NMR spectrum of Compound **5q**(R=*o*-OH Ph) in $CDCl_3$

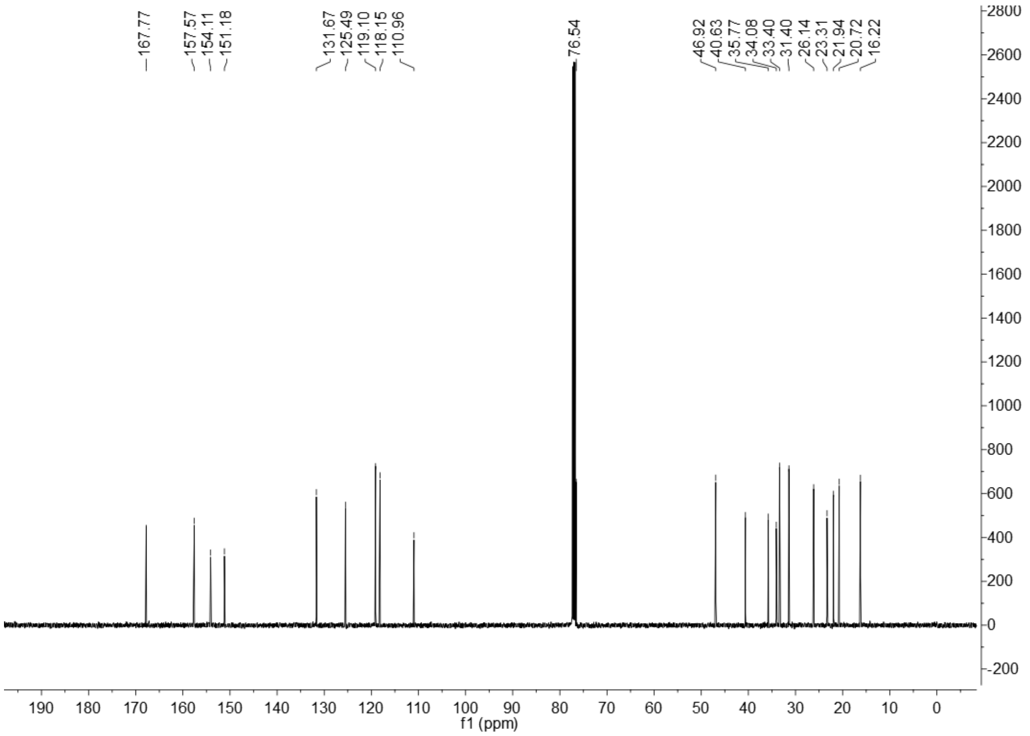

Figure S67 ^{13}C-NMR spectrum of Compound **5q**(R=*o*-OH Ph) in $CDCl_3$.

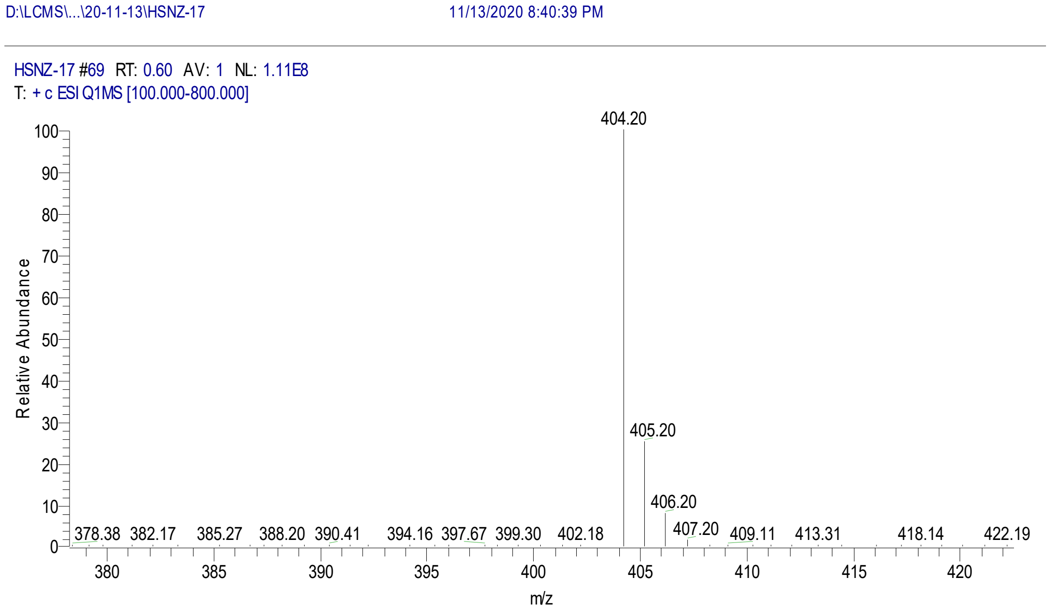

Figure S68. ESI-MS spectrum of Compound **5q**(R=*o*-OH Ph).

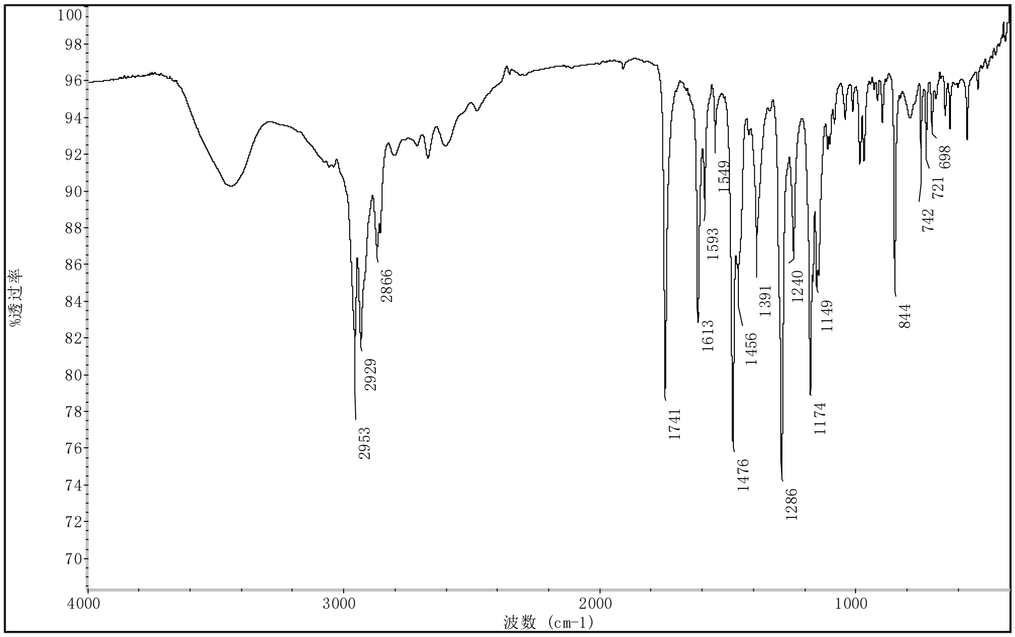

Figure S69. FT-IR spectrum of Compound **5r**(R=*p*-OH Ph)

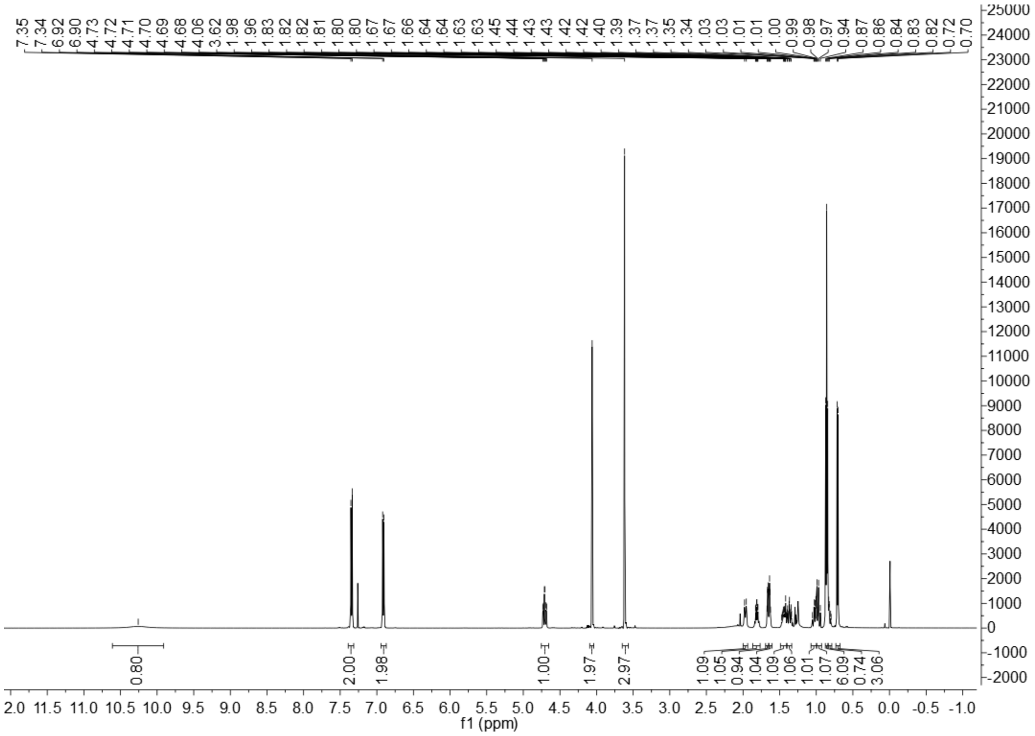

Figure S70. ^{1}H-NMR spectrum of Compound **5r**(R=*p*-OH Ph) in $CDCl_3$

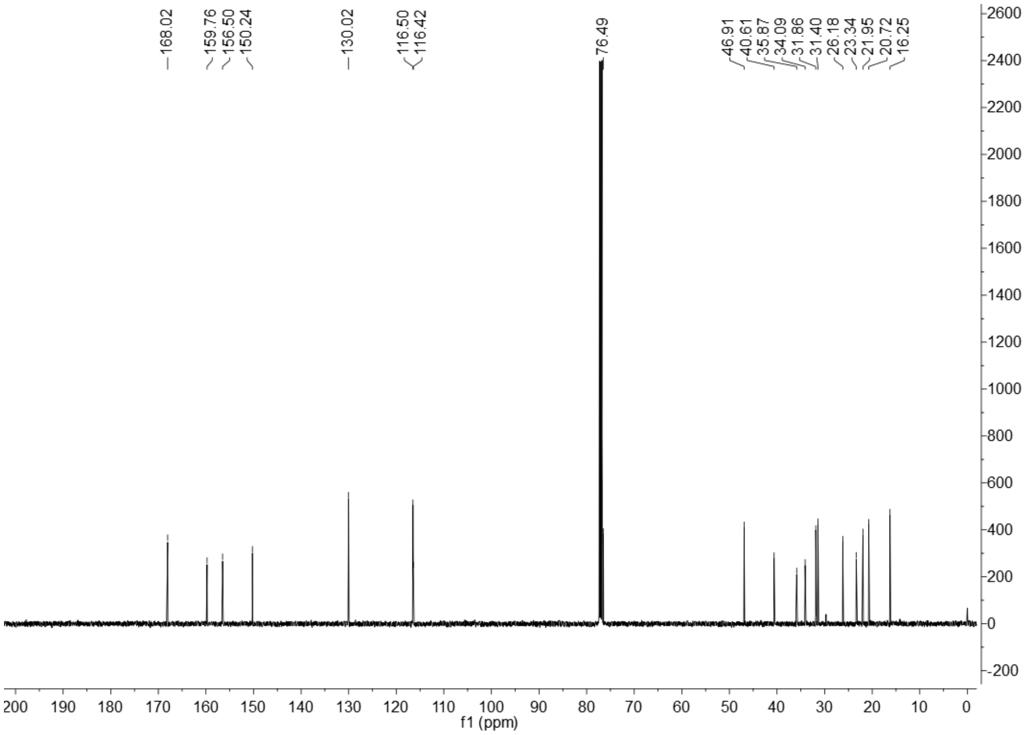

Figure S71 ^{13}C-NMR spectrum of Compound **5r**(R=*p*-OH Ph) in $CDCl_3$.

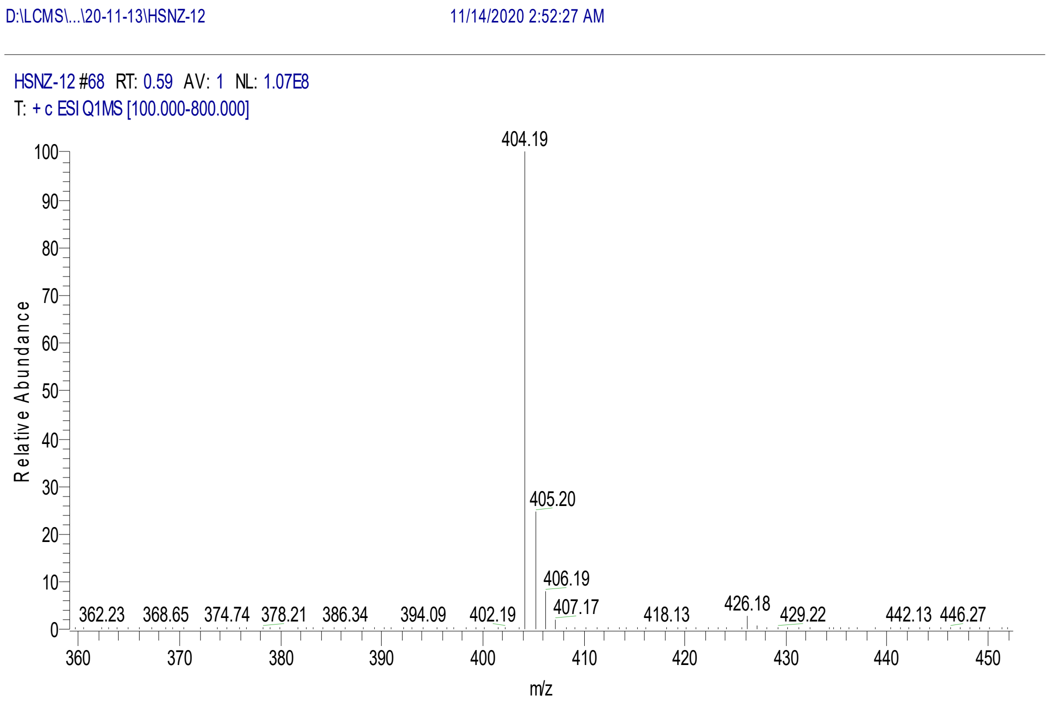

Figure S72. ESI-MS spectrum of Compound **5r**(R=*p*-OH Ph).

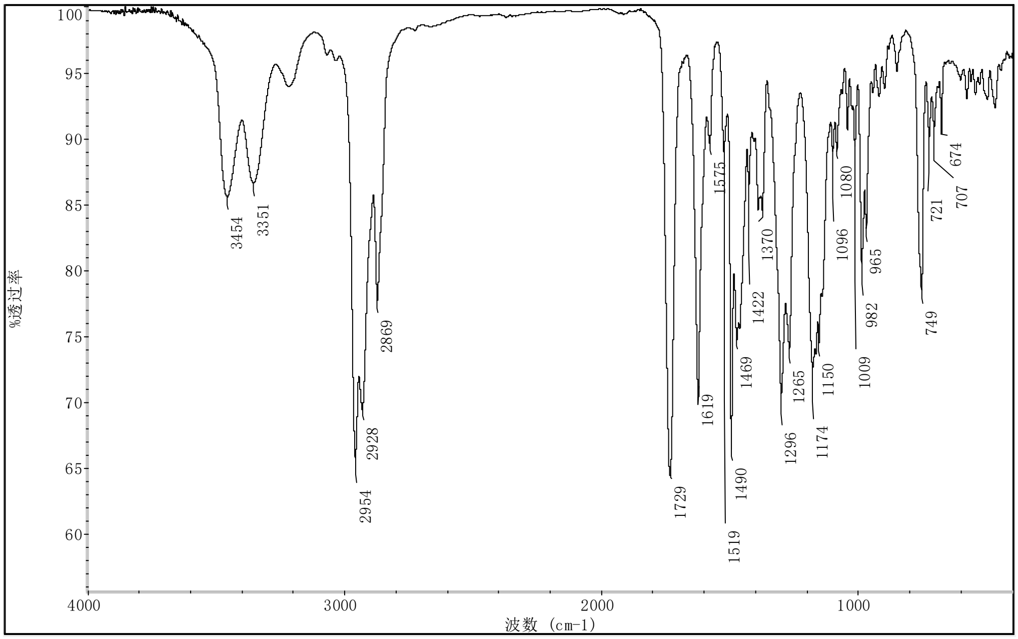

Figure S73. FT-IR spectrum of Compound **5s**(R=*o*-NH_2 Ph)

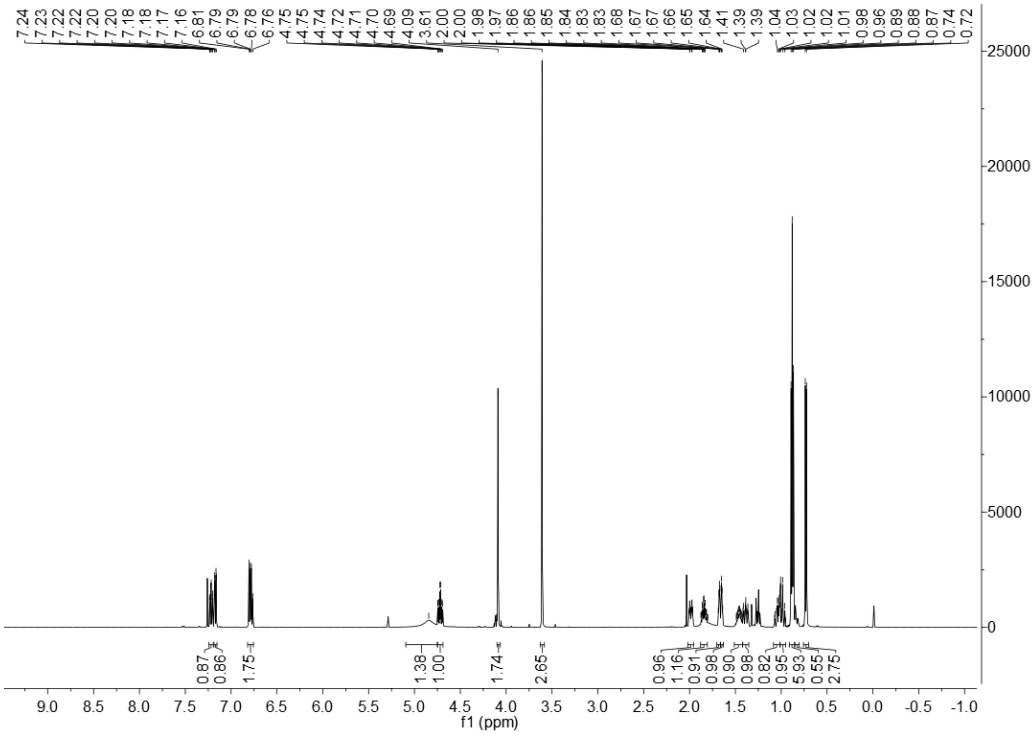

Figure S74. ^{1}H-NMR spectrum of Compound **5s**(R=*o*-NH_2 Ph) in $CDCl_3$

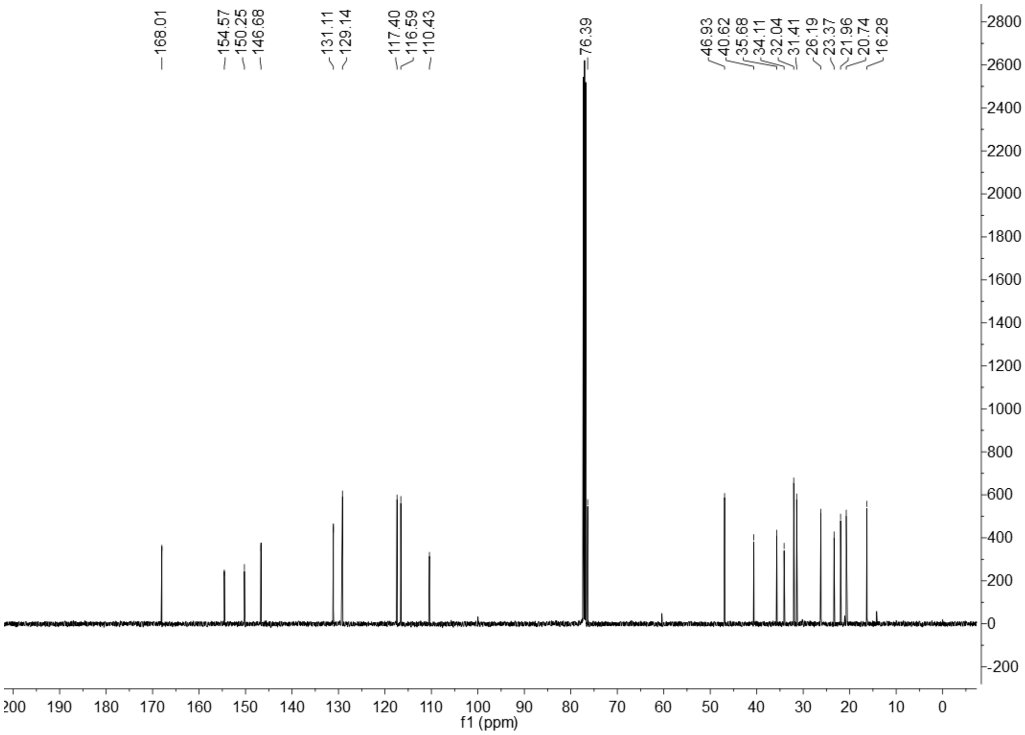

Figure S75 ^{13}C-NMR spectrum of Compound **5s**(R=*o*-NH_2 Ph) in $CDCl_3$.

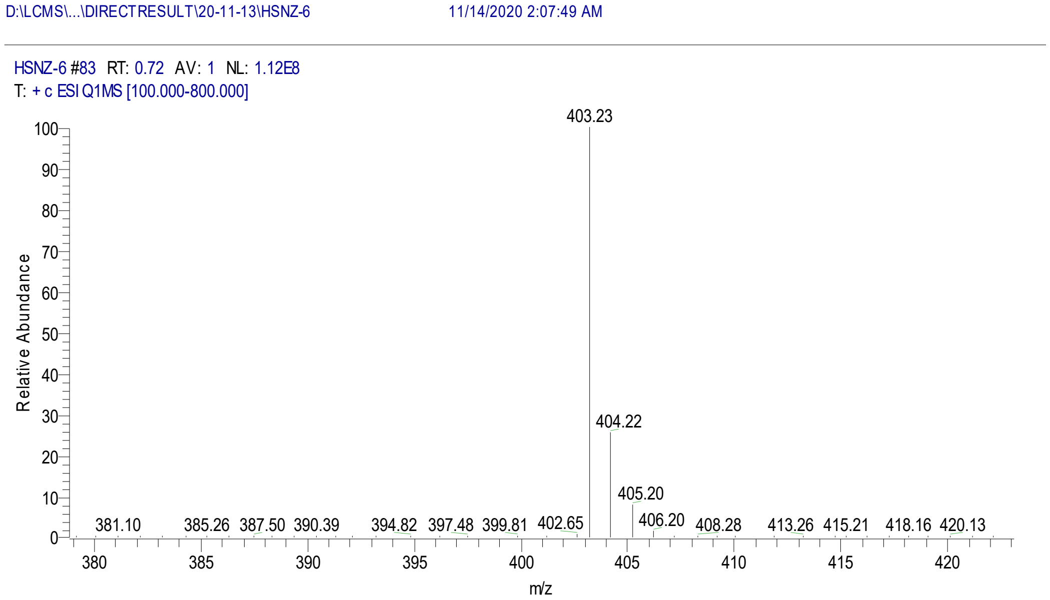

Figure S76. ESI-MS spectrum of Compound **5s**(R=*o*-NH_2 Ph).

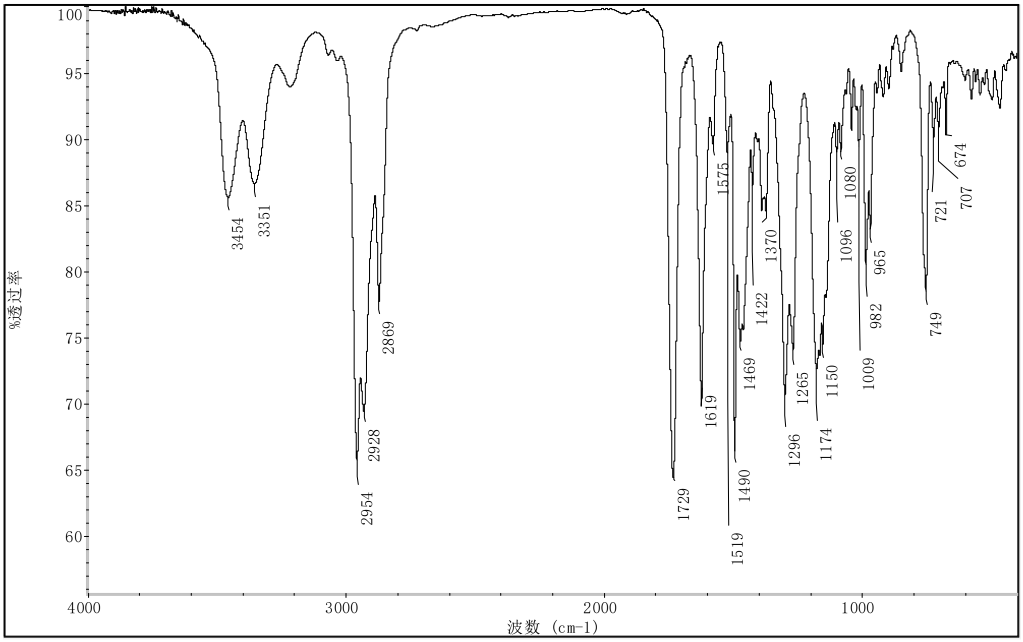

Figure S77. FT-IR spectrum of Compound **5t**(R=*p*-NH_2 Ph)

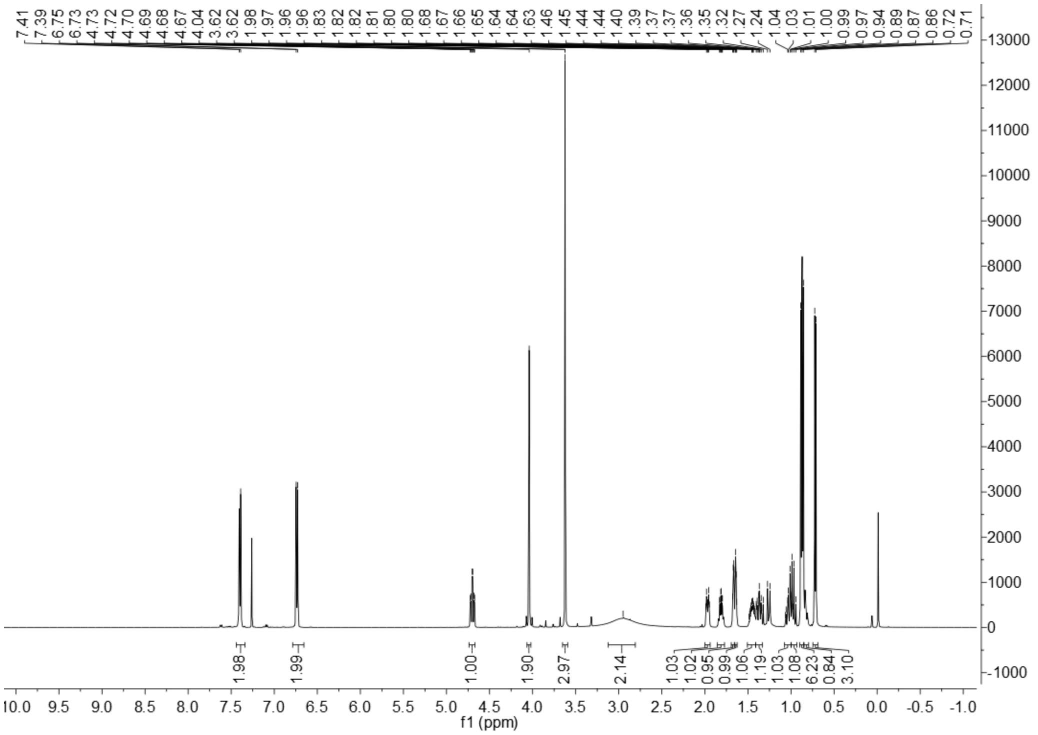

Figure S78. ^{1}H-NMR spectrum of Compound **5t**(R=*p*-NH_2 Ph) in $CDCl_3$

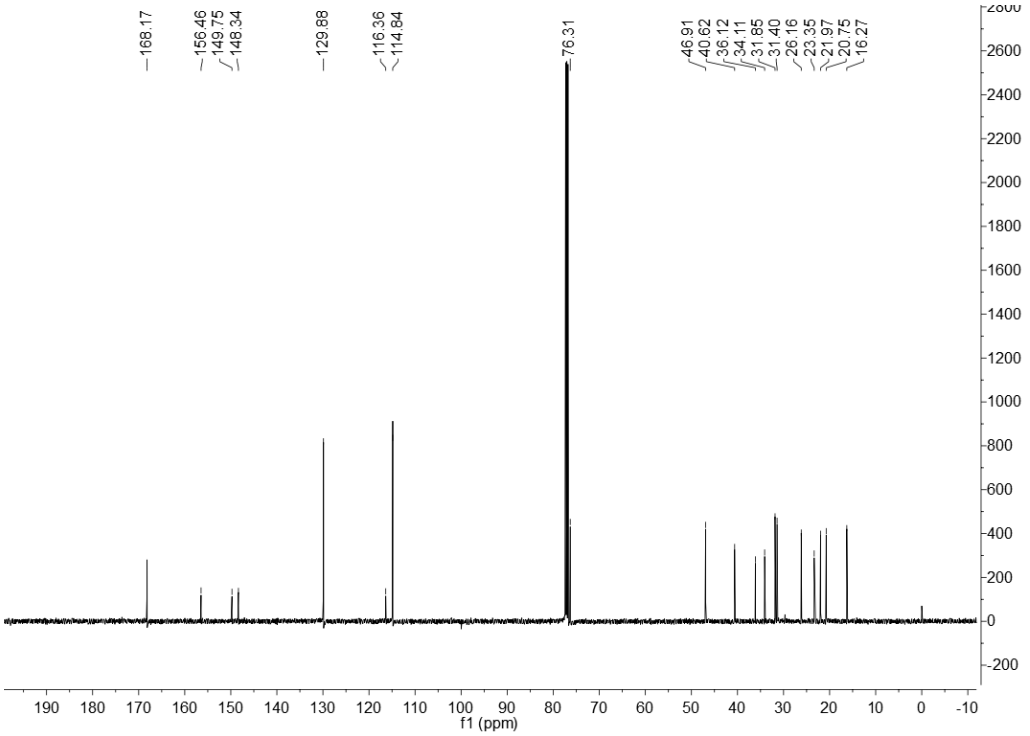

Figure S79 ^{13}C-NMR spectrum of Compound **5t**(R=*p*-NH_2 Ph) in $CDCl_3$.

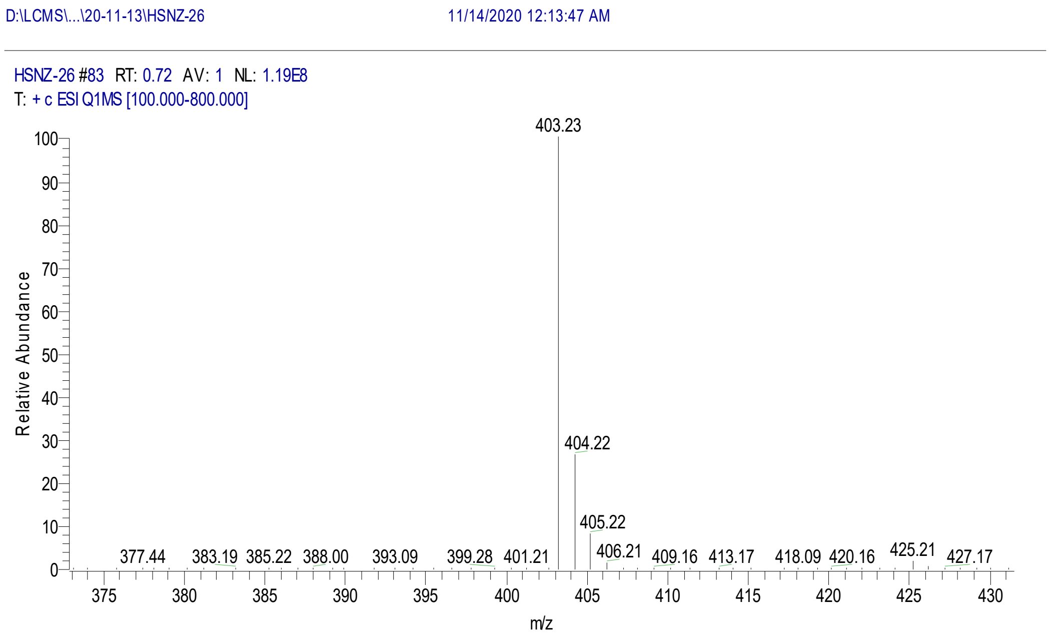

Figure S80. ESI-MS spectrum of Compound **5t**(R=*p*-NH_2 Ph).

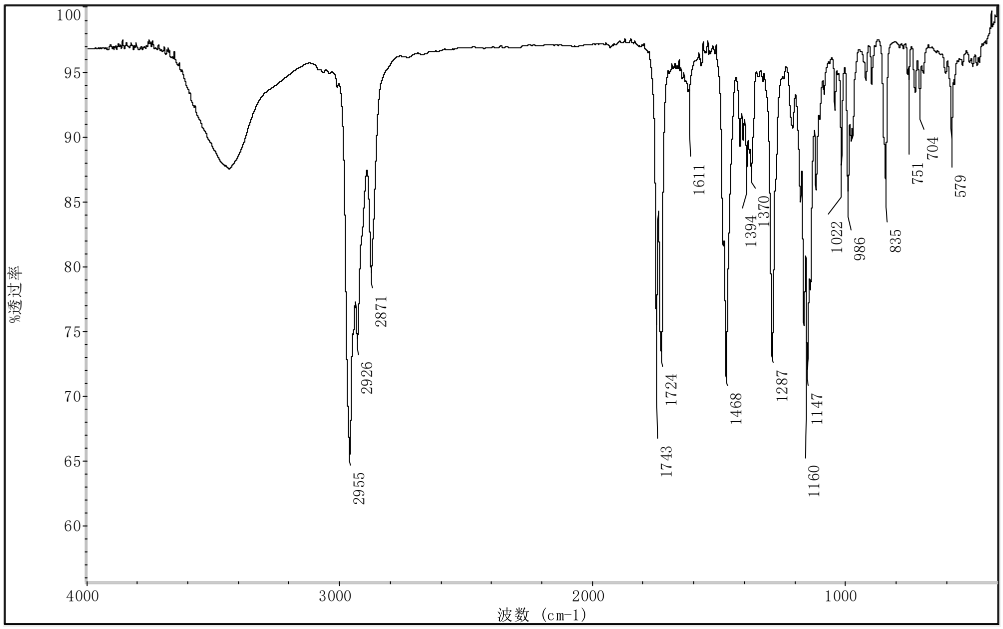

Figure S81. FT-IR spectrum of Compound **5u**(R=*p*-C(CH_3)$_3$ Ph)

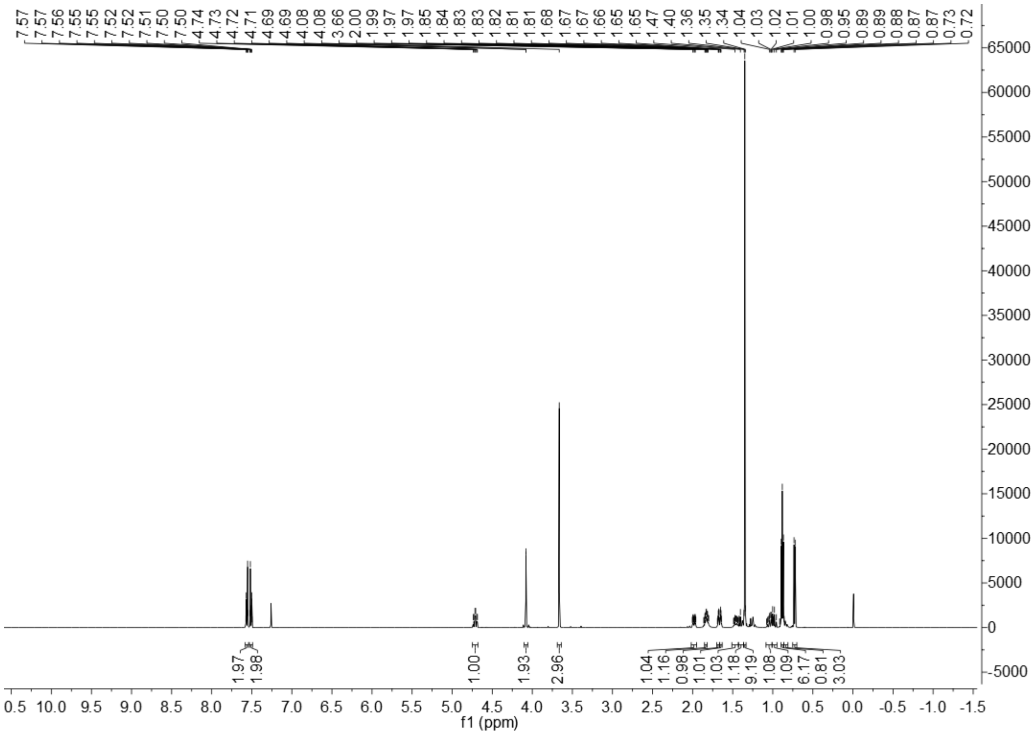

Figure S82. ^{1}H-NMR spectrum of Compound **5u**(R=*p*-C(CH_3)$_3$ Ph) in $CDCl_3$

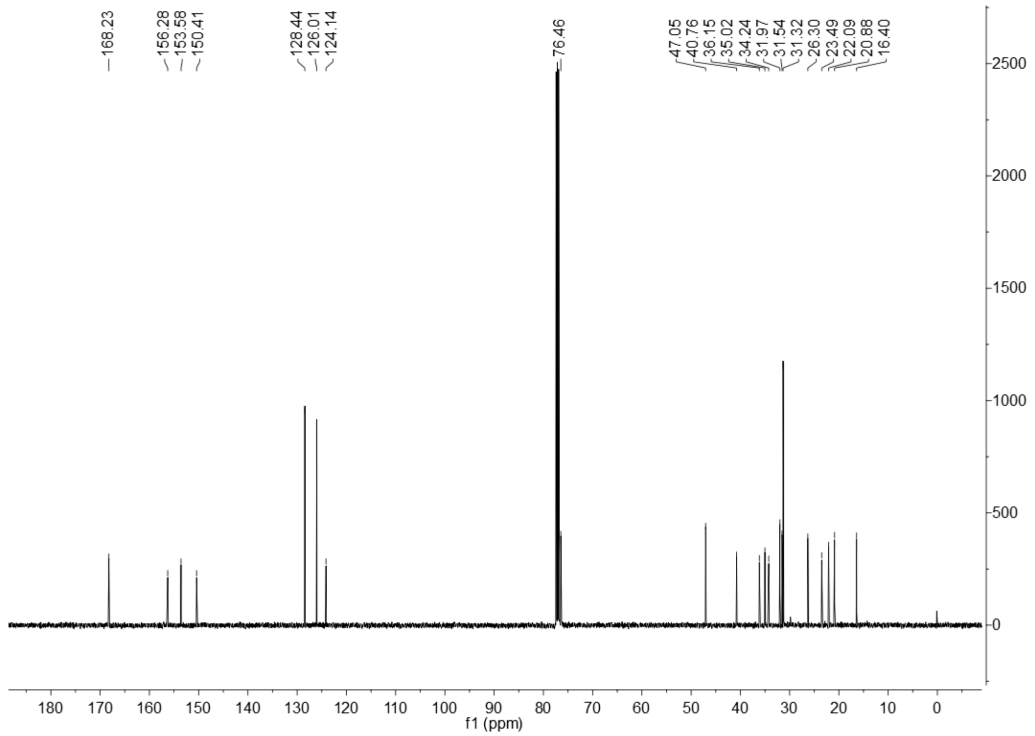

Figure S83 ^{13}C-NMR spectrum of Compound **5u**(R=*p*-C(CH_3)$_3$ Ph) in $CDCl_3$.

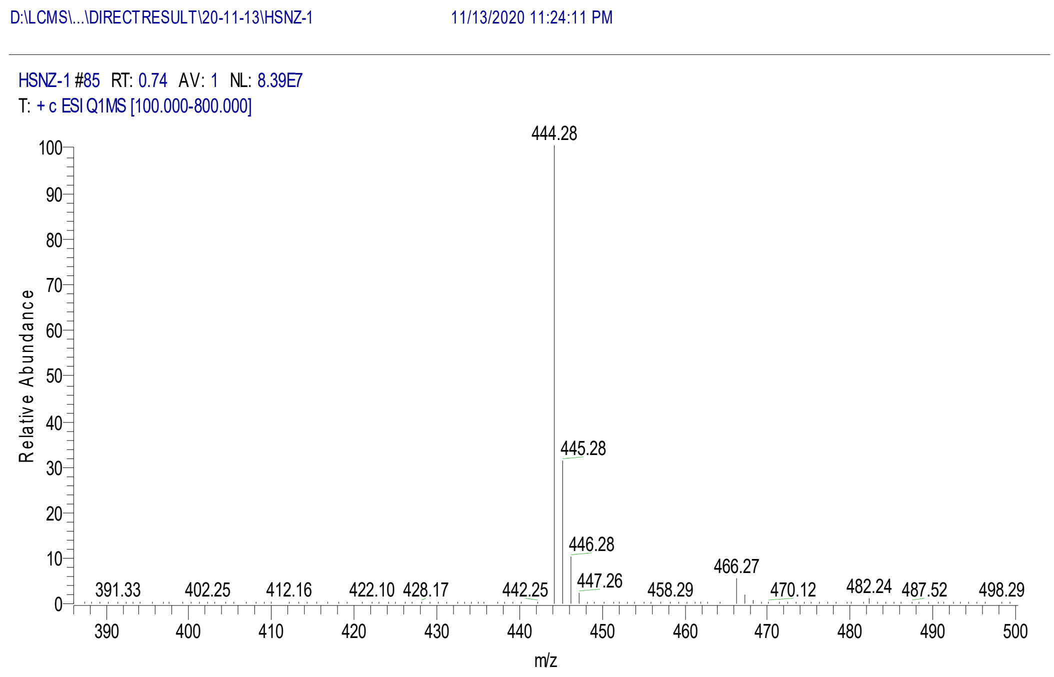

Figure S84. ESI-MS spectrum of Compound **5u**(R=*p*-C(CH_3)$_3$ Ph).

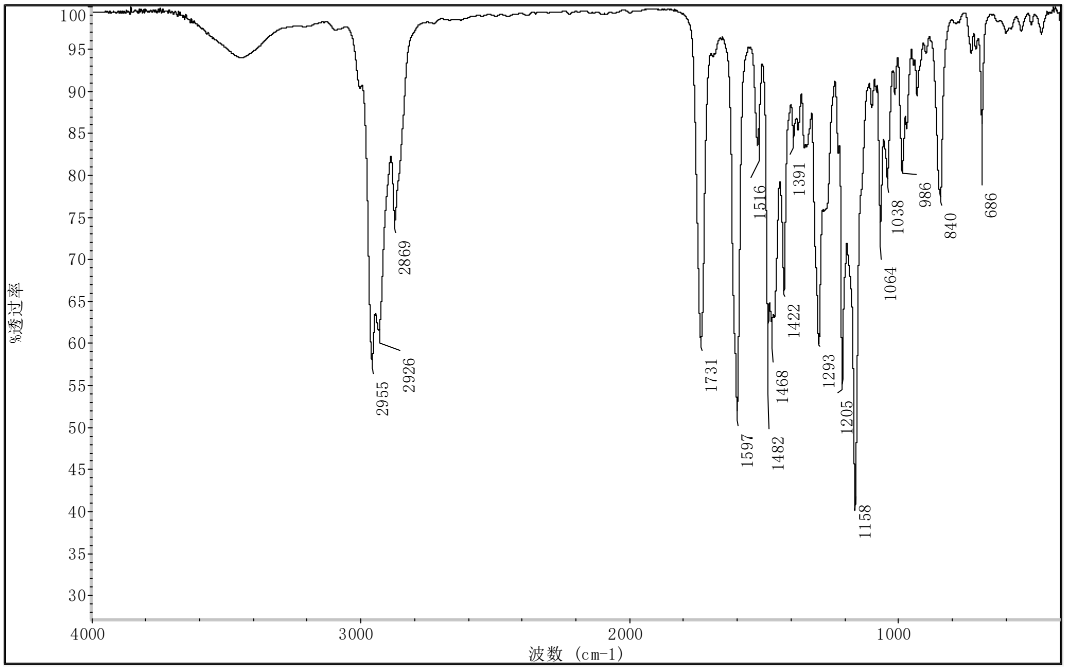

Figure S85. FT-IR spectrum of Compound **5v**(R=*m*,*p*-OCH_3 Ph)

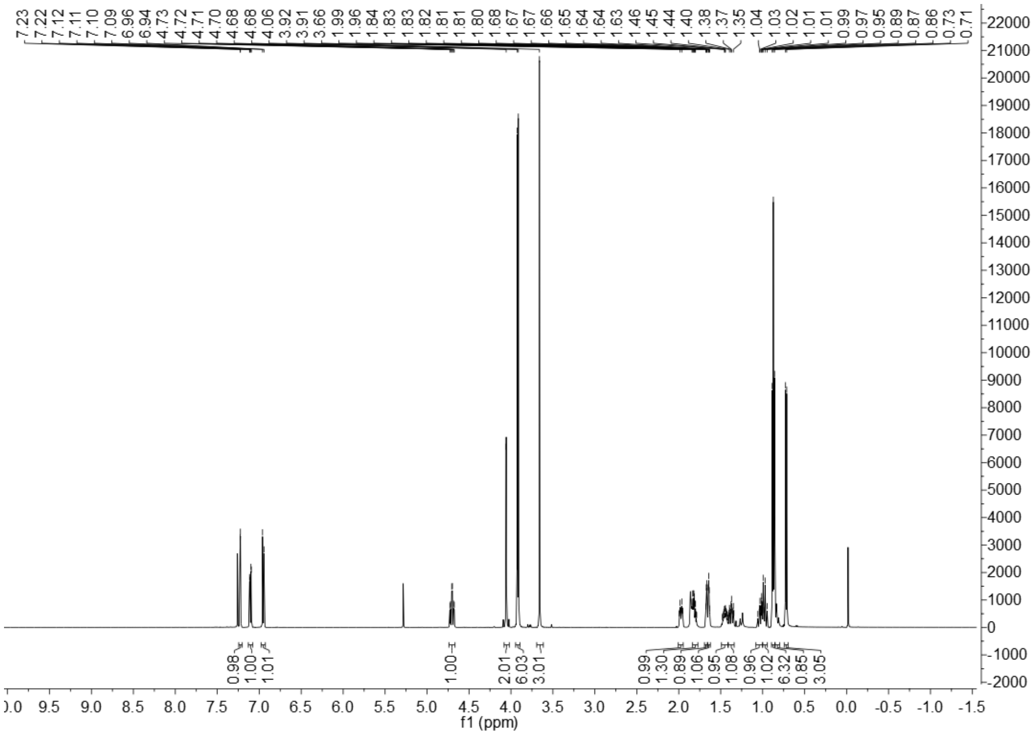

Figure S86. ^{1}H-NMR spectrum of Compound **5v**(R=*m*,*p*-OCH_3 Ph)

in $CDCl_3$

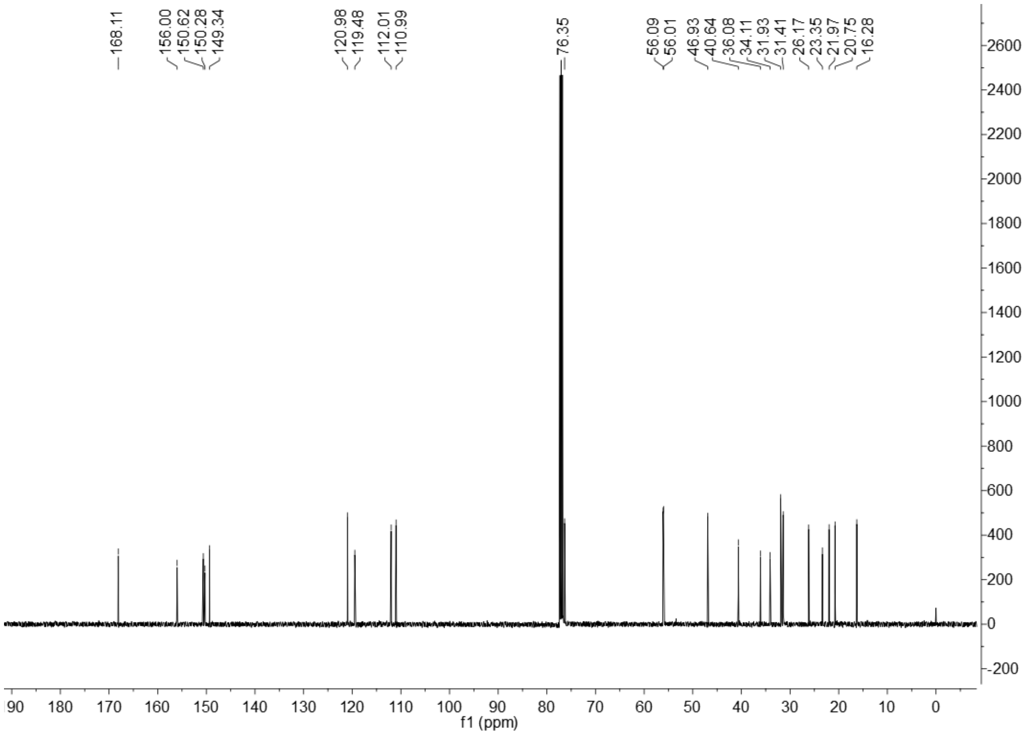

Figure S87 ^{13}C-NMR spectrum of Compound **5v**(R=*m*,*p*-OCH_3 Ph) in $CDCl_3$.

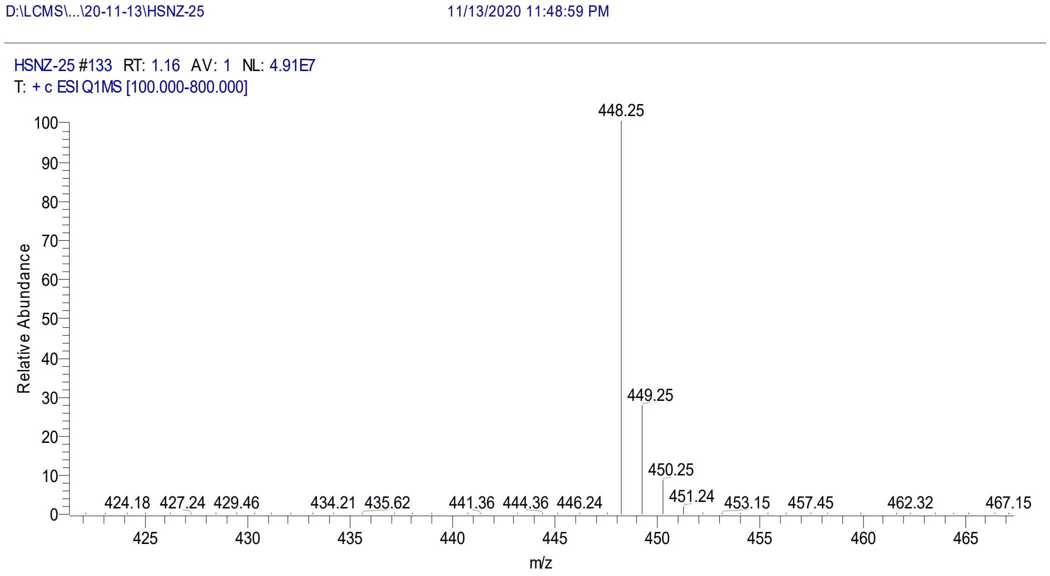

Figure S88. ESI-MS spectrum of Compound **5v**(R=*m*,*p*-OCH_3 Ph).

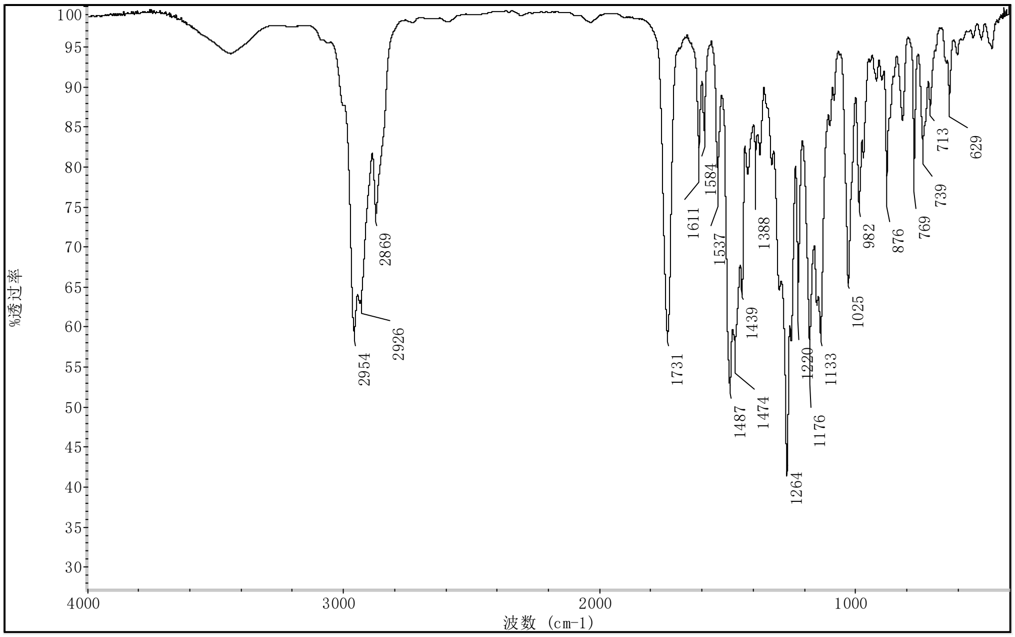

Figure S89. FT-IR spectrum of Compound **5w**(R=*m*,*m*-OCH_3 Ph)

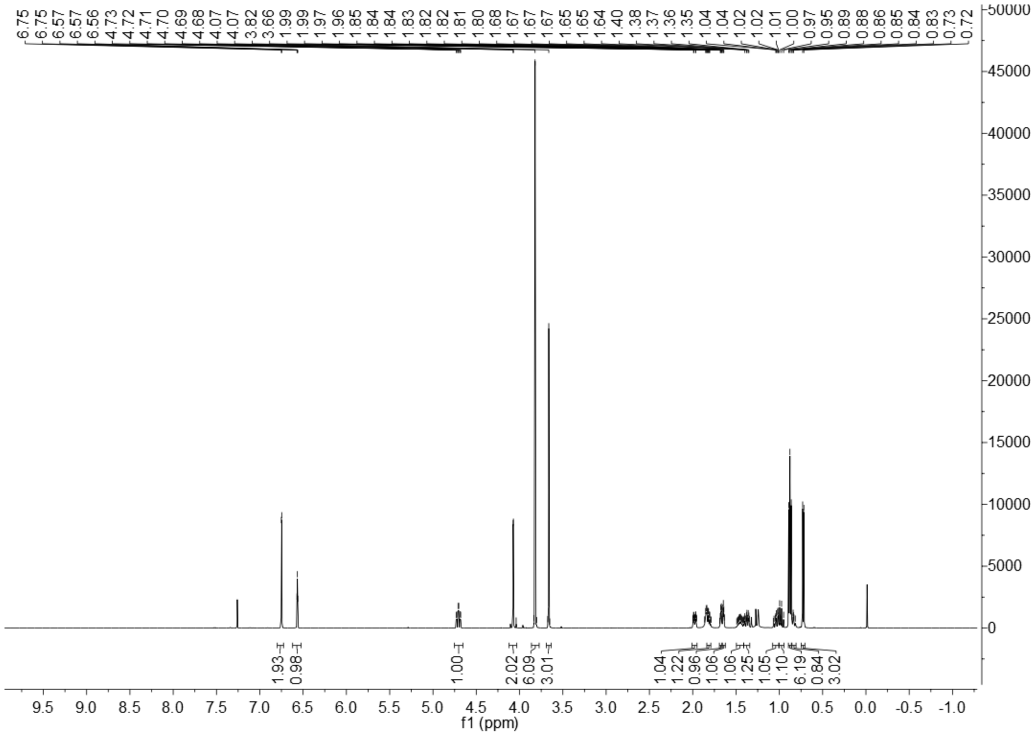

Figure S90. ^{1}H-NMR spectrum of Compound **5w**(R=*m*,*m*-OCH_3 Ph)

in $CDCl_3$

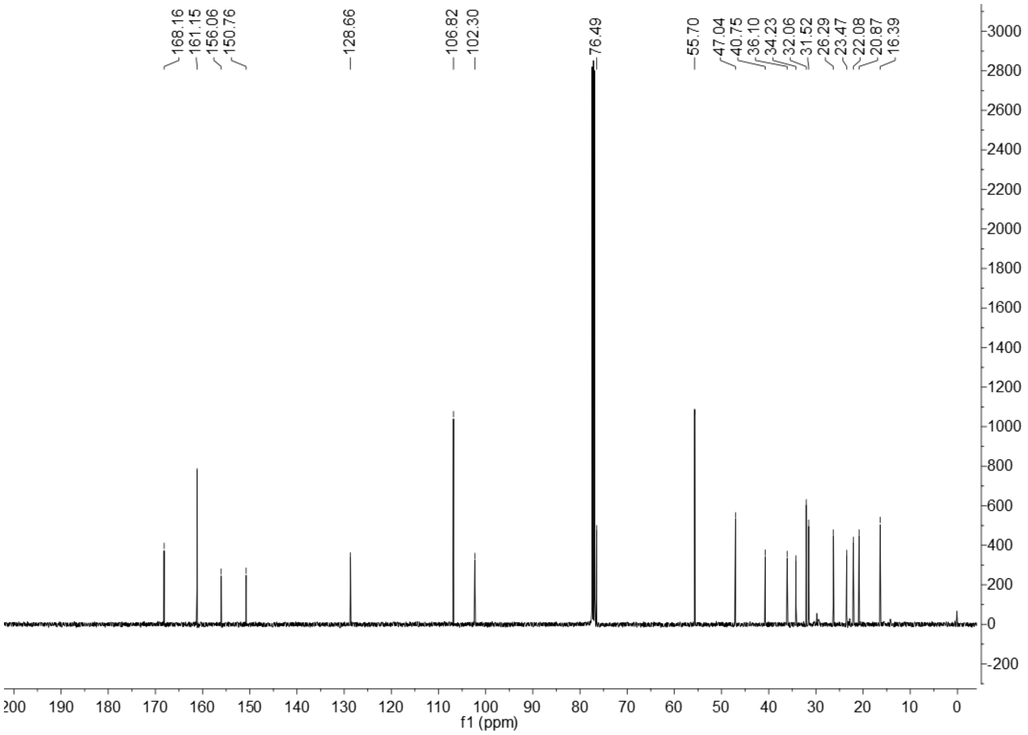

Figure S91 ^{13}C-NMR spectrum of Compound **5w**(R=*m*,*m*-OCH_3 Ph) in $CDCl_3$.

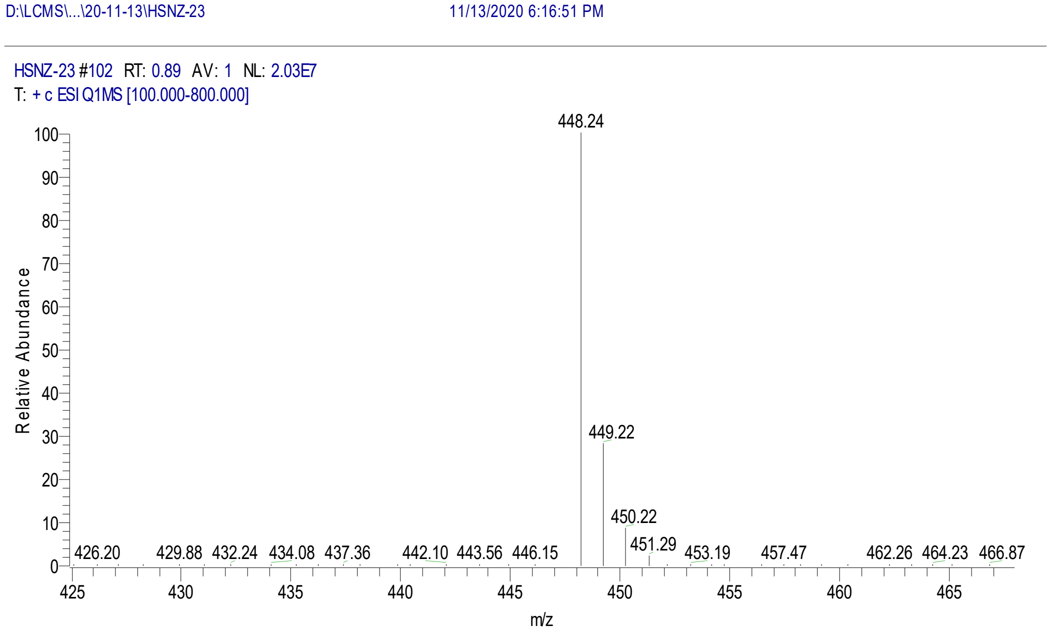

Figure S92. ESI-MS spectrum of Compound **5w**(R=*m*,*m*-OCH_3 Ph).

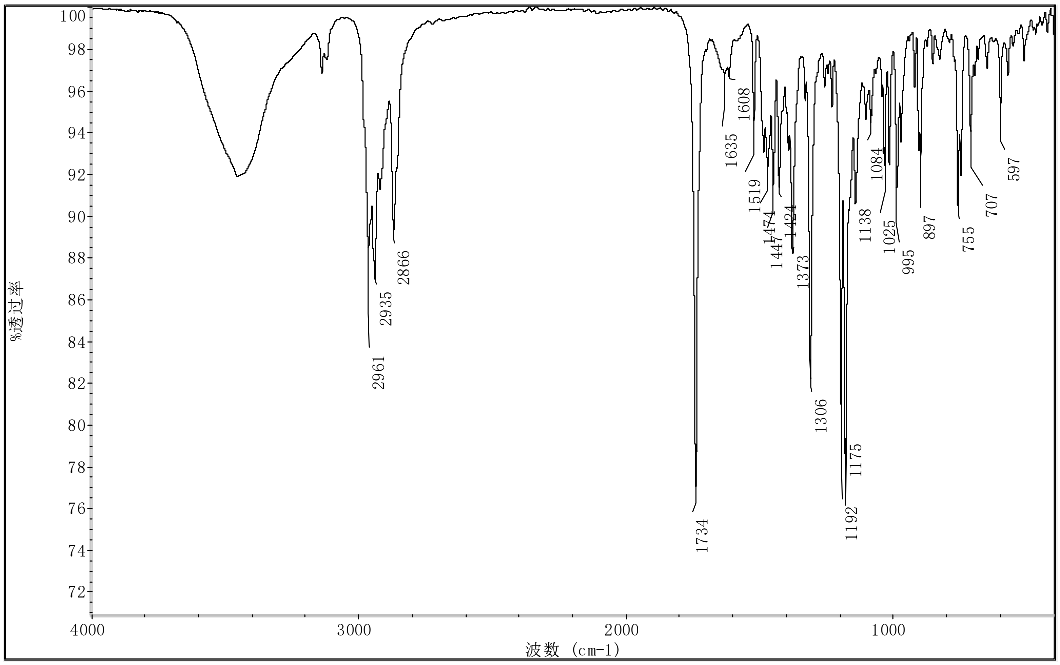

Figure S93. FT-IR spectrum of Compound **5x**(R= α -furyl)

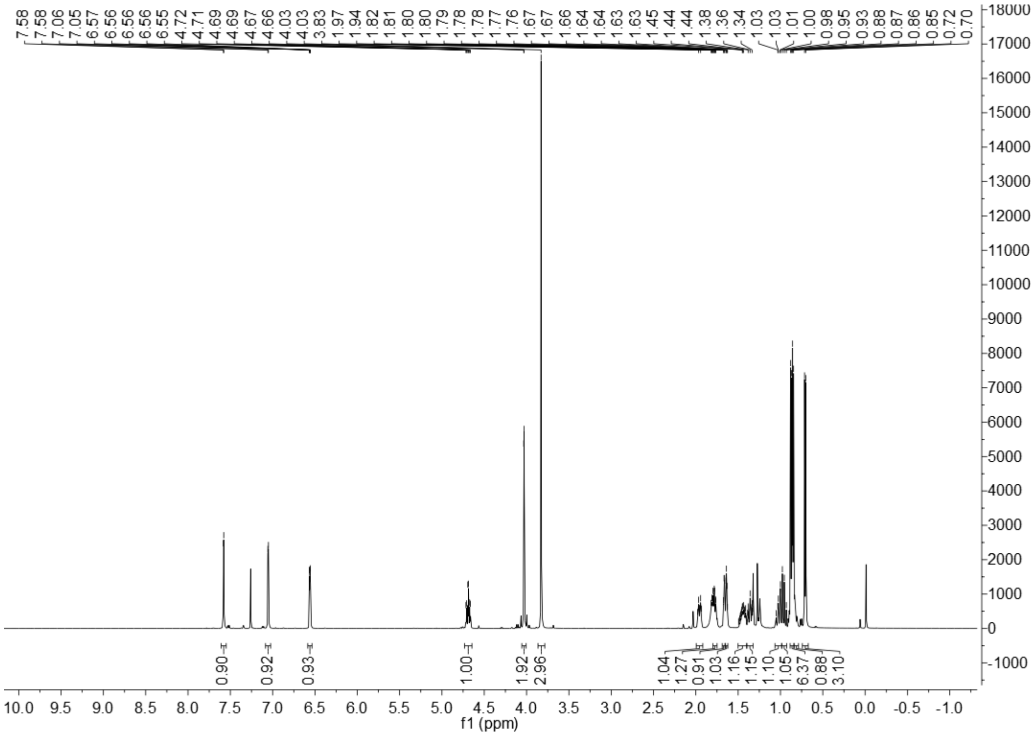

Figure S94. ^{1}H-NMR spectrum of Compound **5x**(R= α -furyl)

in $CDCl_3$

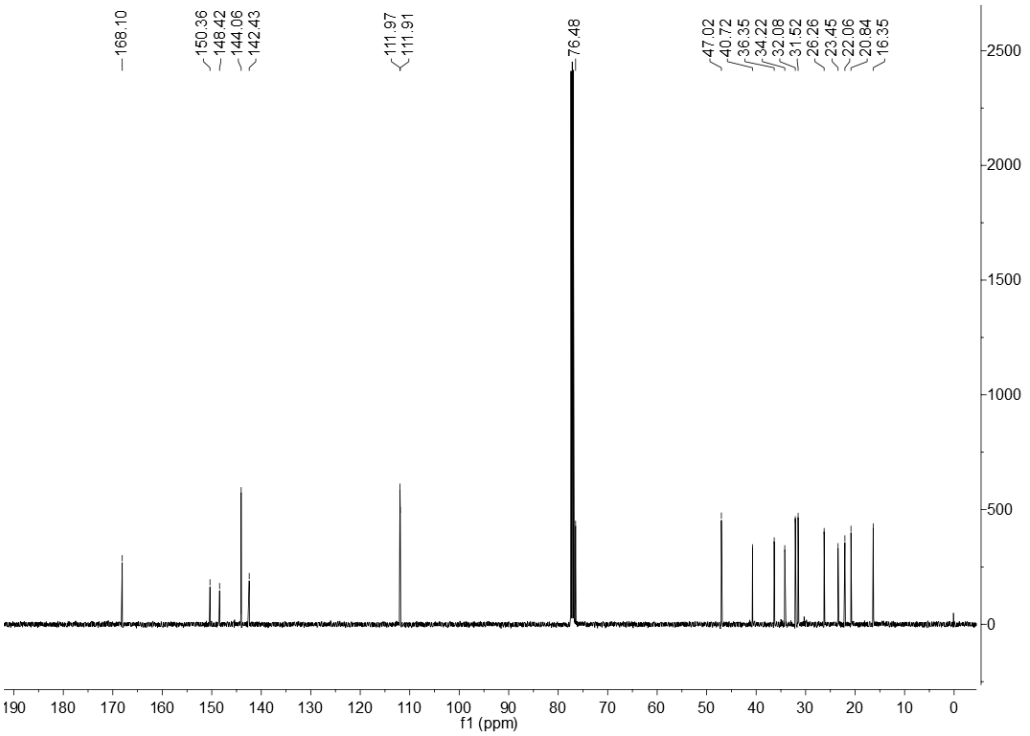

Figure S95 ^{13}C-NMR spectrum of Compound **5x**(R= α -furyl)

in $CDCl_3$.

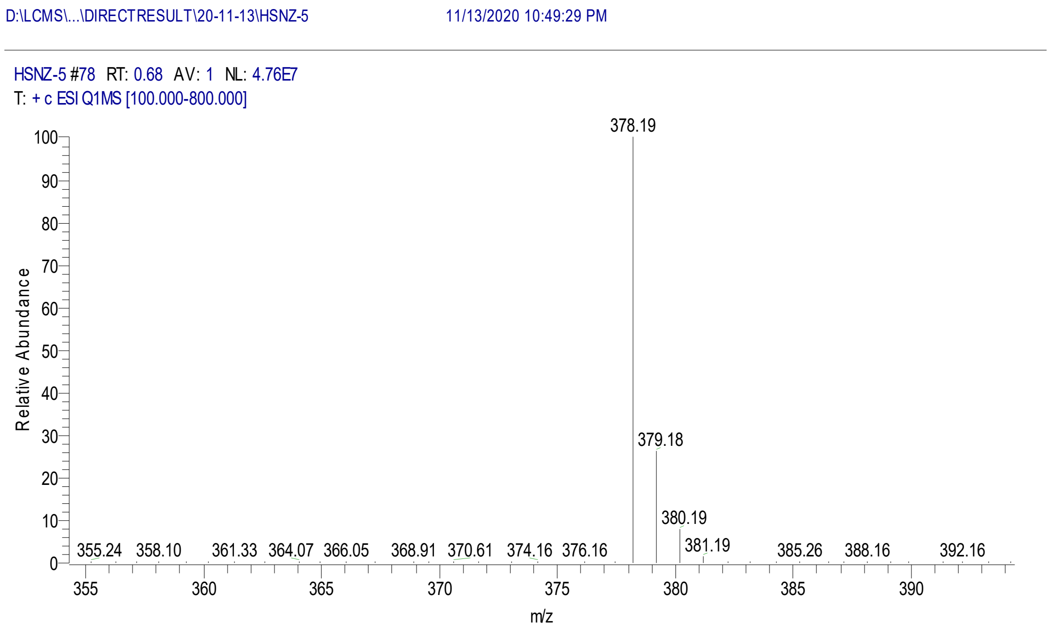

Figure S96. ESI-MS spectrum of Compound **5x**(R= α -furyl).

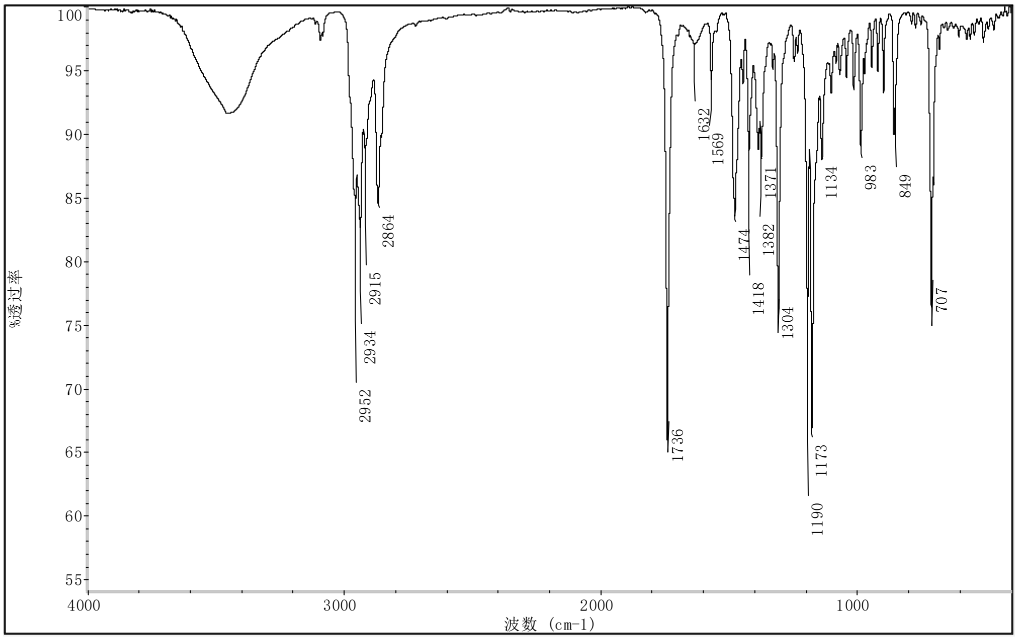

Figure S97. FT-IR spectrum of Compound **5y**(R= α -thienyl)

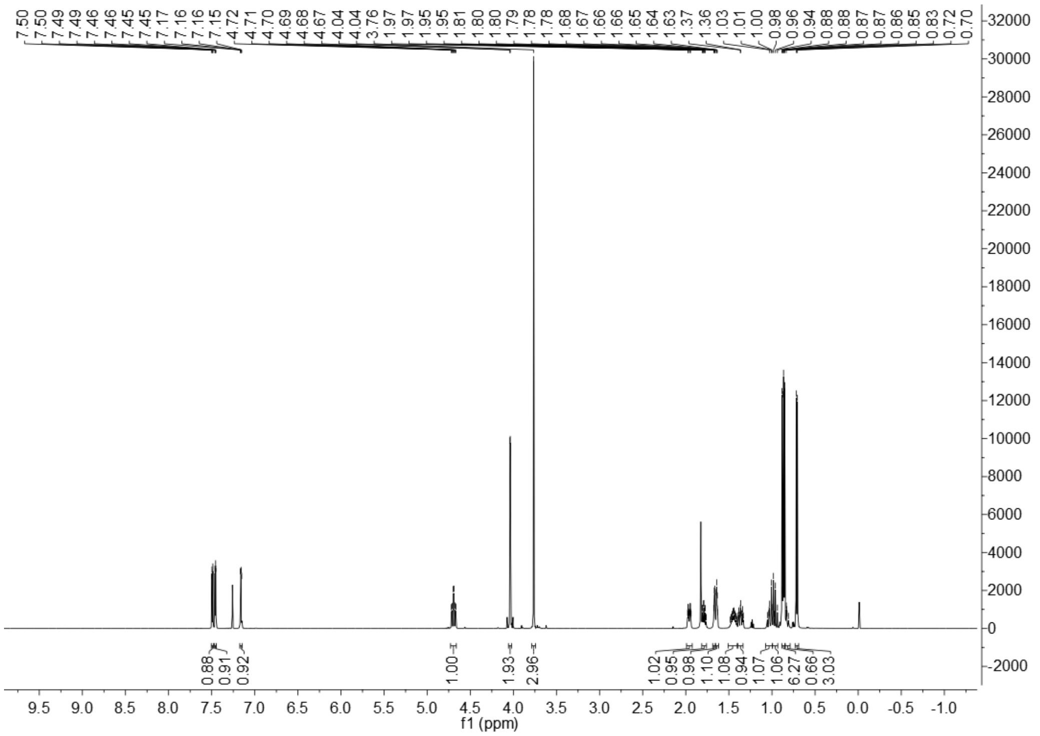

Figure S98. ^{1}H-NMR spectrum of Compound **5y**(R= α -thienyl)

in $CDCl_3$

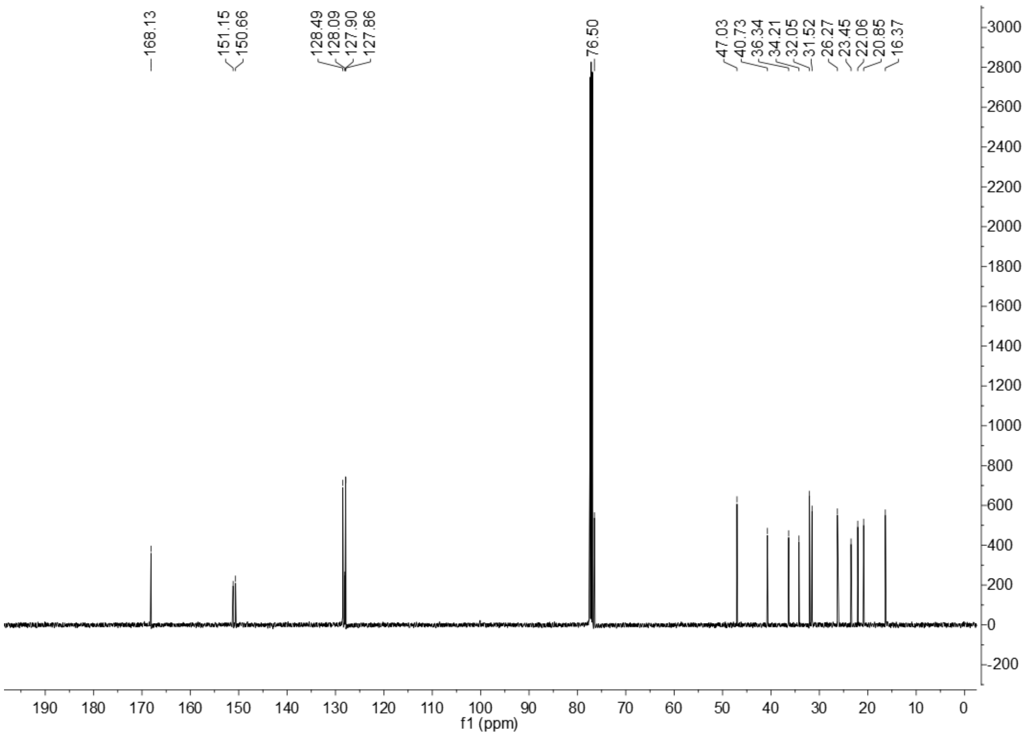

Figure S99 ^{13}C-NMR spectrum of Compound **5y**(R= α -thienyl)

in $CDCl_3$.

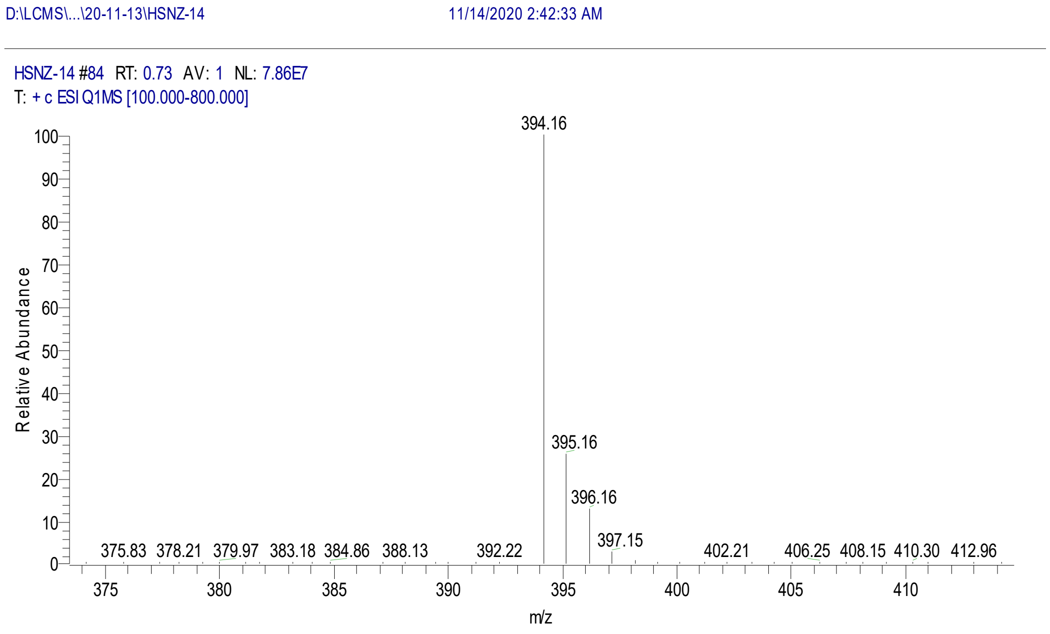

Figure S100. ESI-MS spectrum of Compound **5y**(R= α -thienyl).

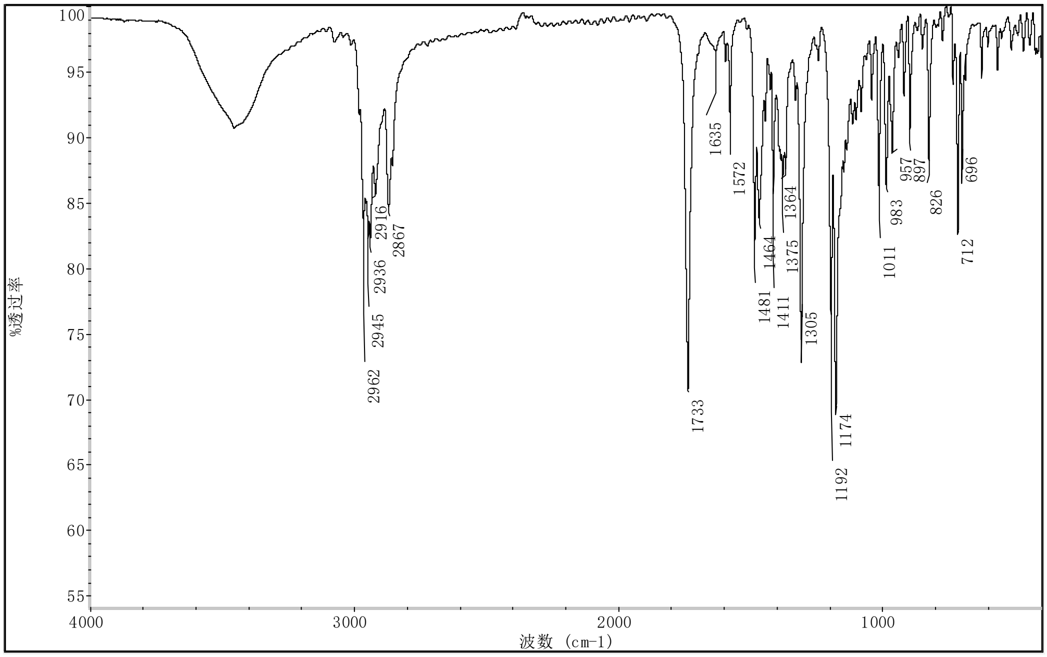

Figure S101. FT-IR spectrum of Compound **5z**(R=*β*-pyridyl)

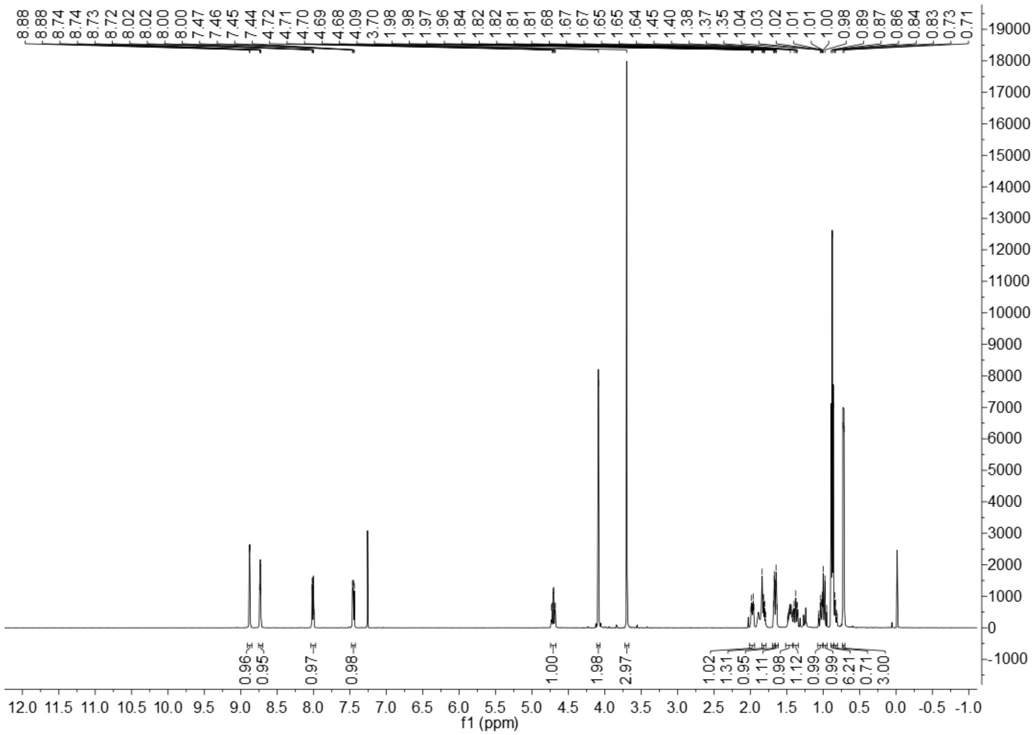

Figure S102. ^{1}H-NMR spectrum of Compound **5z**(R=*β*-pyridyl) in $CDCl_3$

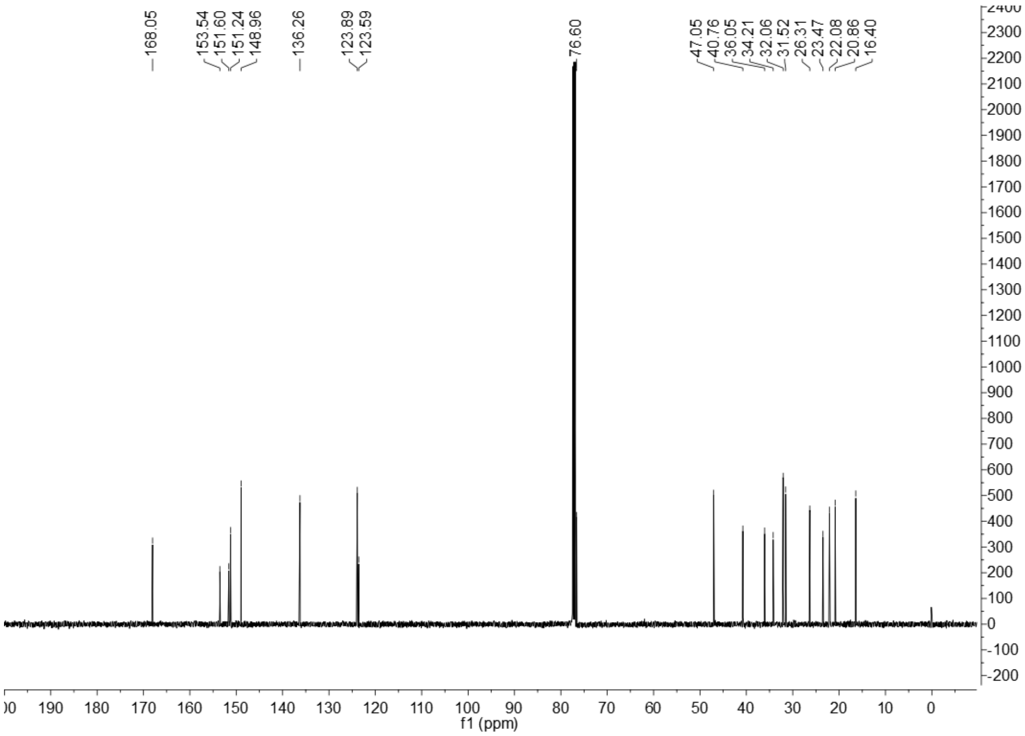

Figure S103 ^{13}C-NMR spectrum of Compound **5z**(R=*β*-pyridyl) in $CDCl_3$.

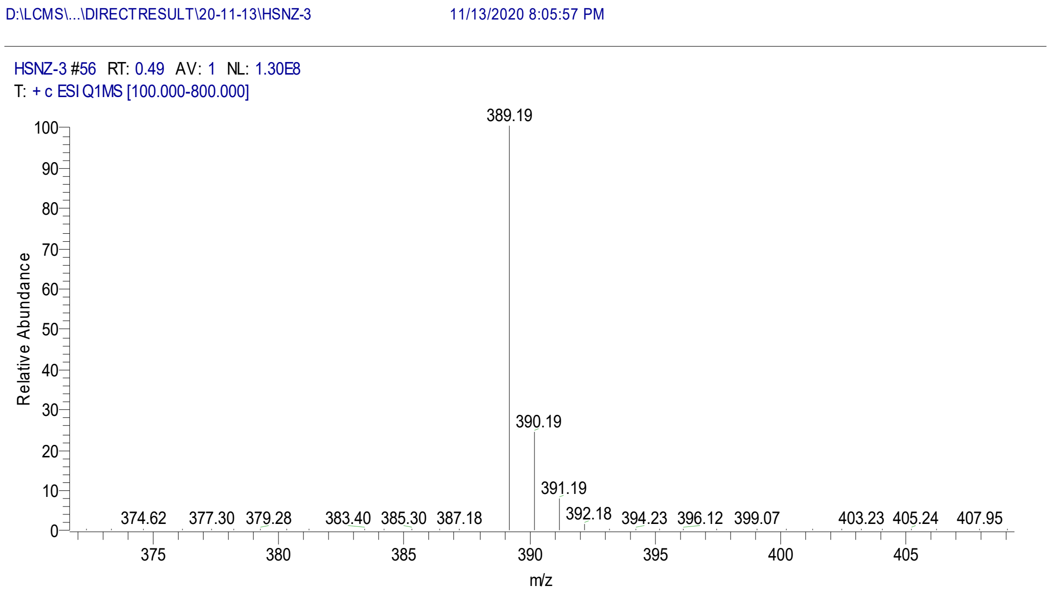

Figure S104. ESI-MS spectrum of Compound **5z**(R=*β*-pyridyl).

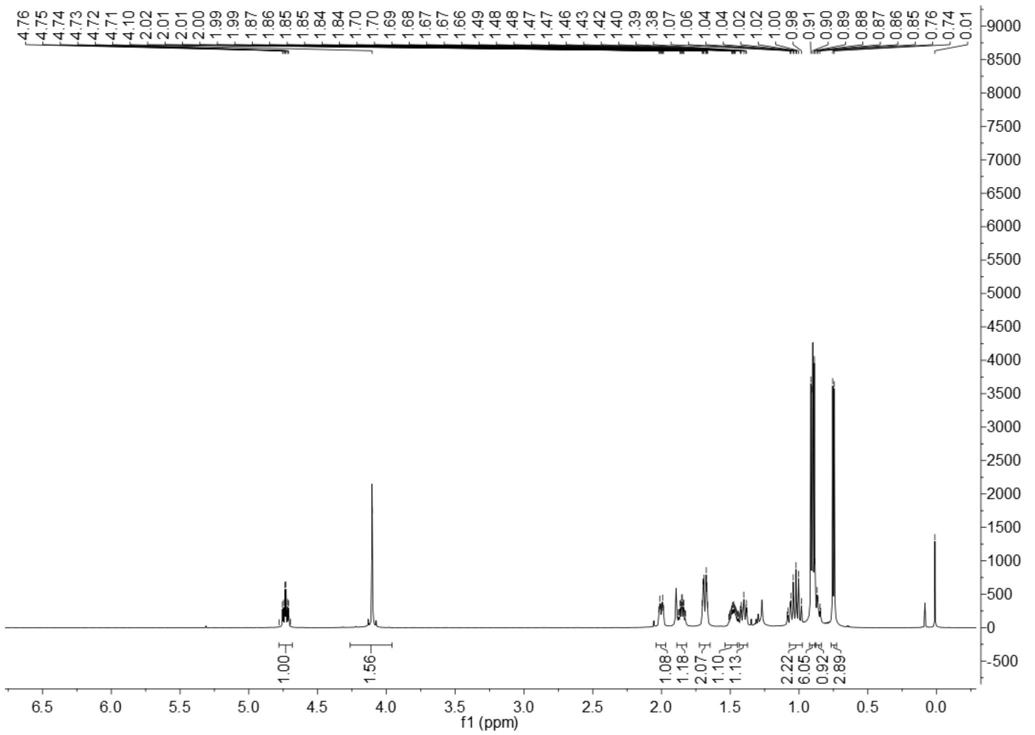

Figure S105. ^{1}H-NMR spectrum of Compound **2** in $CDCl_3$

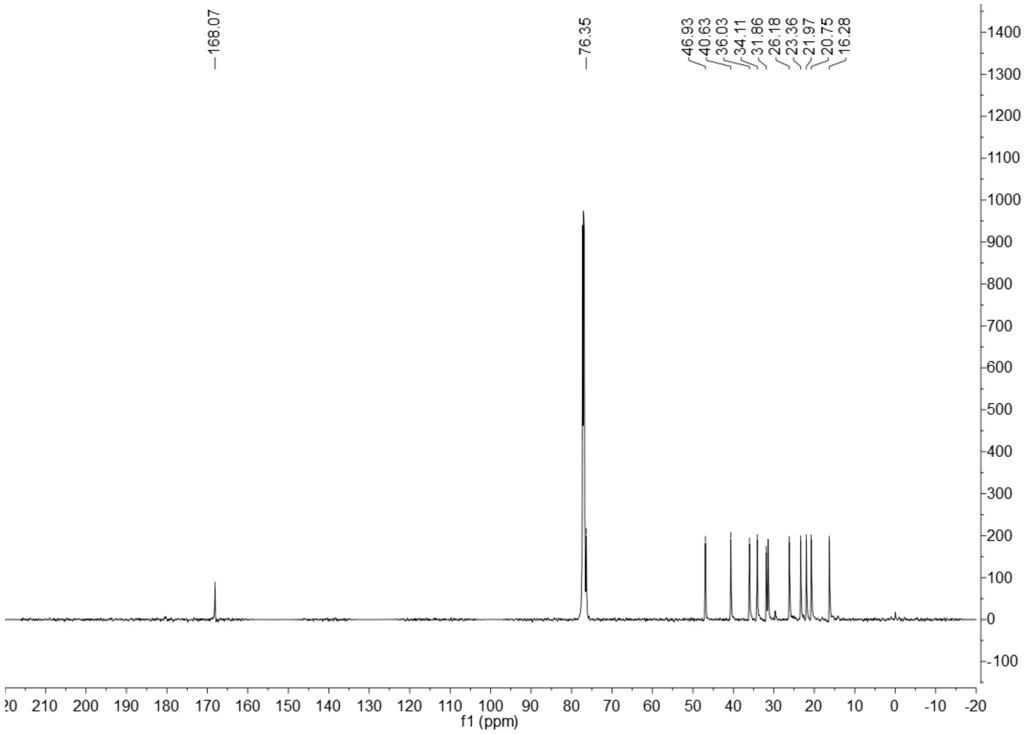

Figure S106 ^{13}C-NMR spectrum of Compound **2** in $CDCl_3$.
